# GPSM1 impairs metabolic homeostasis by controlling a pro-inflammatory pathway in macrophages

**DOI:** 10.1038/s41467-022-34998-9

**Published:** 2022-11-25

**Authors:** Jing Yan, Yuemei Zhang, Hairong Yu, Yicen Zong, Daixi Wang, Jiangfei Zheng, Li Jin, Xiangtian Yu, Caizhi Liu, Yi Zhang, Feng Jiang, Rong Zhang, Xiangnan Fang, Ting Xu, Mingyu Li, Jianzhong Di, Yan Lu, Xinran Ma, Jian Zhang, Weiping Jia, Cheng Hu

**Affiliations:** 1 Shanghai Diabetes Institute, Shanghai Key Laboratory of Diabetes Mellitus, Shanghai Clinical Centre for Diabetes, Shanghai Sixth People’s Hospital Affiliated to Shanghai Jiao Tong University School of Medicine, Shanghai, China; 2 Clinical Research Center, Shanghai Sixth People’s Hospital Affiliated to Shanghai Jiao Tong University School of Medicine, Shanghai, China; 3 Department of Bariatric and Metabolic Surgery, Shanghai Sixth People’s Hospital Affiliated to Shanghai Jiao Tong University School of Medicine, Shanghai, China; 4 Medicinal Chemistry and Bioinformatics Center, Shanghai Jiao Tong University School of Medicine, Shanghai, China; 5 Institute of Metabolism and Regenerative Medicine, Shanghai Sixth People’s Hospital Affiliated to Shanghai Jiao Tong University School of Medicine, Shanghai, China; 6 Shanghai Key Laboratory of Regulatory Biology, Institute of Biomedical Sciences and School of Life Sciences, East China Normal University, Shanghai, China; 7 Medicinal Chemistry and Bioinformatics Center & Shanghai Institute of Hematology, State Key Laboratory of Medical Genomics, National Research Center for Translational Medicine at Shanghai, Ruijin Hospital, Shanghai Jiao Tong University School of Medicine, Shanghai, China; 8 School of Pharmaceutical Sciences, Zhengzhou University, Zhengzhou, China; 9 Institute for Metabolic Disease, Fengxian Central Hospital Affiliated to Southern Medical University, Shanghai, China

**Keywords:** Type 2 diabetes, Obesity, Chronic inflammation

## Abstract

G-protein-signaling modulator 1 (*GPSM1*) exhibits strong genetic association with Type 2 diabetes (T2D) and Body Mass Index in population studies. However, how GPSM1 carries out such control and in which types of cells are poorly understood. Here, we demonstrate that myeloid GPSM1 promotes metabolic inflammation to accelerate T2D and obesity development. Mice with myeloid-specific *GPSM1* ablation are protected against high fat diet-induced insulin resistance, glucose dysregulation, and liver steatosis via repression of adipose tissue pro-inflammatory states. Mechanistically, GPSM1 deficiency mainly promotes TNFAIP3 transcription via the Gα_i3_/cAMP/PKA/CREB axis, thus inhibiting TLR4-induced NF-κB signaling in macrophages. In addition, we identify a small-molecule compound, AN-465/42243987, which suppresses the pro-inflammatory phenotype by inhibiting GPSM1 function, which could make it a candidate for metabolic therapy. Furthermore, *GPSM1* expression is upregulated in visceral fat of individuals with obesity and is correlated with clinical metabolic traits. Overall, our findings identify macrophage GPSM1 as a link between metabolic inflammation and systemic homeostasis.

## Abstract (web summary)

G-protein-signaling modulator 1 (GPSM1), an accessory protein which activates heterotrimeric G-protein signaling, exhibits a genetic association with type 2 diabetes. Here the authors show that myeloid GPSM1 ablation in mice inhibits inflammation and metabolic dysfunction upon high fat diet.

## Introduction

Activator of G protein signaling 3 (AGS3), encoded by G-protein-signaling modulator 1 (*GPSM1*), is an accessory protein which activates heterotrimeric G-protein signaling^1–3^. To date, a series of genome-wide association studies (GWASs) of East Asian and European populations have identified *GPSM1* as a susceptible gene of type 2 diabetes (T2D)^4–6^. Nevertheless, the molecular mechanism underlying the effect of *GPSM1* on T2D and such metabolic disorders remains unknown.

Recent evidence has showed that activation of GPSM1 is involved in immune responses^7–9^. For example, GPSM1 is required for efficient chemokine receptor signaling of lymphocytes, bone marrow-derived dendritic cells, and leukocytes during acute inflammation^8^. Furthermore, expression of GPSM1 is elevated in human monocyte-like cell line THP-1 following lipopolysaccharide (LPS) activation, suggesting that GPSM1 also acts as a pro-inflammatory effector in monocyte-macrophages^10^. However, it remains unclear whether GPSM1 in immune cells would exert any effects on chronic metabolic inflammation.

It is widely known that overnutrition-induced adipose tissue inflammation is a hallmark of obesity and can contribute to the development of insulin resistance and T2D^11,12^. In white adipose tissue (WAT) of obese mice and humans, various innate and adaptive immune cells infiltrate and subsequently produce pro-inflammatory cytokines and chemokines, such as tumor necrosis factor-α (TNF-α), Interleukin-6 (IL-6), IL-1β, and C-C motif ligand-2 (CCL2), that compromise insulin-glucose homeostasis^13,14^. As the major effector cells orchestrating chronic inflammatory responses, adipose tissue macrophages (ATMs) are present in distinct activation states based upon their inflammatory phenotype, thereby contributing to homeostasis in the adipose tissue micro-environment. In obesity, ATMs generally polarize to the predominantly pro-inflammatory phenotype, namely, M1-like macrophages, which have been found in the ‘crown-like’ structure (CLS) around dying adipocytes. M1 cells are proposed to promote insulin resistance and impair adipose health^13,15,16^. However, lean fat contains a high abundance of anti-inflammatory M2-like macrophages^13,17^. Therefore, alleviating the M1 macrophages infiltrating as well as inhibiting the pro-inflammatory program in M1 macrophages may reduce pathological remodeling of WAT, thus ameliorating the progression of T2D and metabolic diseases.

We hypothesized that obesity could similarly lead to GPSM1 activation in macrophages and GPSM1 may serve as a link between inflammation and metabolic homeostasis. Here, we delete *GPSM1* exclusively in myeloid lineage cells to investigate its role in metabolic homeostasis. We report an important function of the macrophage GPSM1 in the control of metabolic inflammation and systemic insulin resistance and metabolic disturbance in mouse models. We find that GPSM1, when activated by LPS in macrophages, suppresses *TNFAIP3* transcription via the Gα_i3_/cAMP/PKA/CREB axis, thereby stimulating NF-κB signaling. Moreover, GPSM1 expression is upregulated in WAT from individuals with obesity and correlates with clinical metabolic traits. Further high-throughput virtual screening (HTVS) with 270,000 compounds, combined with Biacore and High-content screen (HCS) analysis identifies a potential small-molecule compound that could inhibit GPSM1 function. The results of the present study suggest that part of the underlying molecular mechanism of the effect of a T2D susceptibility gene, *GPSM1*, on T2D and obesity progression is through macrophage inflammation, and suggest that targeting GPSM1 is could be an approach for the treatment of T2D and obesity.

## Results

### Macrophage GPSM1 expression is upregulated in murine obesity

**Fig. 1:**
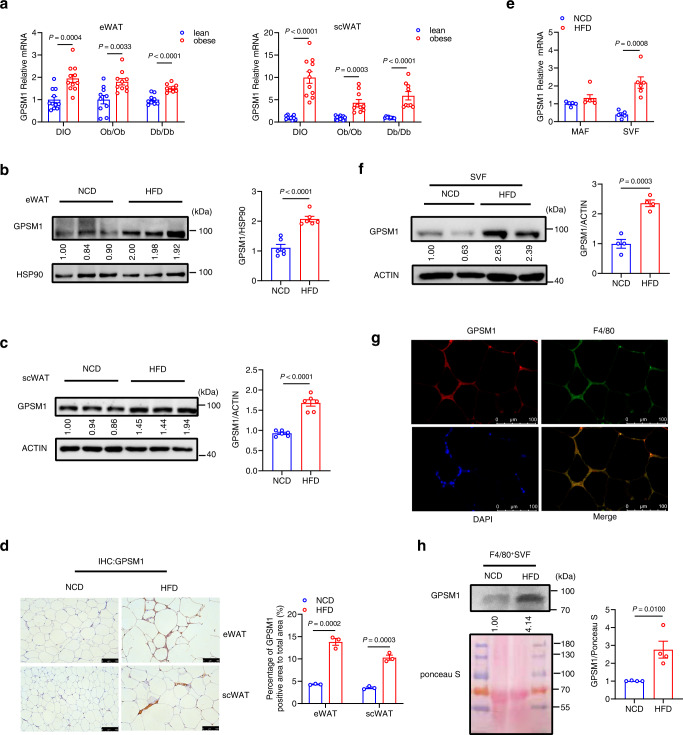
GPSM1 expression is upregulated in ATMs from obese mice. **a** RT-qPCR analysis of *GPSM1* mRNA expression in epididymal WAT (eWAT) and subcutaneous WAT (scWAT) from lean (blue) or obese (red) mice. For DIO, WT male mice were fed normal chow diet (NCD) (*n* = 11 biologically independent sample) or high-fat diet (HFD) (*n* = 11 biologically independent sample) for 12 weeks. For genetic obesity, a group of WT (*n* = 10 biologically independent mice) and *Ob/Ob* (*n* = 10 biologically independent mice) and a separate group of WT (*n* = 9 biologically independent mice) and *Db/Db* (*n* = 9 biologically independent mice) were analyzed. Representative western blot analysis and quantification of GPSM1 expression in eWAT (**b**) and scWAT (**c**) from NCD and HFD mice (n = 6 biologically independent sample per group). **d** IHC staining and quantitative analysis of GPSM1 in eWAT and scWAT from NCD and HFD mice (*n* = 3 biologically independent sample per group). Scale bars, 100 μm. **e** RT-qPCR analysis of *GPSM1* mRNA expression in stromal vascular fraction (SVF) and adipocyte fraction (MAF) isolated from eWAT from NCD (*n* = 5 biologically independent mice) or HFD (*n* = 6 biologically independent mice). **f** Representative western blot analysis and quantification of GPSM1 expression in SVFs isolated of eWAT (*n* = 4 biologically independent mice per group). **g** Immunofluorescence images of staining with antibodies against GPSM1 (red) and F4/80 (green) in eWAT of HFD-fed mice. Nuclei were stained with DAPI (blue). Scale bars, 100 μm. **h** Representative western blot analysis and quantification of GPSM1 expression in sorted F4/80^+^ macrophages isolated from eWAT SVFs (*n* = 4 biologically independent mice per group). Ponceau S was used as a loading control. Independent experiments were repeated three times with similar results (**g**). All data are presented as means ± SEM (**a**–**f**, **h**). *P* values are determined by two-tailed Student’s *t-*test (**a**–**f**, **h**).

To understand the role of GPSM1 in metabolic diseases, we first explored whether obesity impacts GPSM1 expression in mouse models. Compared to lean controls, mRNA levels of *GPSM1* in both epididymal WAT (eWAT) and subcutaneous WAT (scWAT) were significantly higher in mice with high-fat diet (HFD)-induced obesity (DIO) or genetic obesity caused by leptin (Ob/Ob) or leptin receptor (Db/Db) deficiency (Fig. 1a). A similar expression trend was reproduced by Western blot analysis and immunohistochemistry (IHC) staining (Fig. 1b–d). Furthermore, fractionation studies indicated that obesity enhanced *GPSM1* exclusively in the stromal vascular fraction (SVF), where ATMs were enriched, but not in the adipocytes of eWAT (Fig. 1e, f). Leptin (Lep) and CD45 were included as controls for mature adipocytes and stromal vascular cells, respectively (Supplementary Fig. 1a, b). As SVFs consists of various cell types, we found that GPSM1 co-localized mainly with F4/80^+^ macrophages in the adipose tissue based on immunofluorescence staining (Fig. 1g). Thus, we further isolated F4/80^+^ macrophages from SVFs using F4/80^+^ microbeads, and we confirmed that obesity could induce GPSM1 overexpression in macrophages (Fig. 1h). In summary, obesity upregulates GPSM1 expression in ATMs, suggesting that GPSM1 may be involved in obesity-associated inflammation.

### Myeloid GPSM1 deficiency protects against diet-induced obesity and systemic metabolic deterioration

To elucidate the role of macrophage GPSM1 in the pathogenesis of metabolic disorders, we intercrossed mice bearing a conditional loxP-flanked (‘floxed’) allele of *GPSM1* (*GPSM1*^f/f^, used as control mice) with the Lysozyme 2-Cre (*Lyz2*-Cre) line^18^ to create myeloid-specific *GPSM1*-knockout (*GPSM1*^f/f^; *Lyz2*-Cre) mice. As expected, *GPSM1* was efficiently ablated in bone-marrow-derived macrophages (BMDMs), elicited-peritoneal macrophages (PMs), and blood monocytes, but not in eWAT and scWAT, as well as other tissues examined (Supplementary Fig. 2a), from *GPSM1*^f/f^; *Lyz2*-Cre mice. *GPSM1*^f/f^; *Lyz2*-Cre mice were born in a Mendelian ratio, and no defective developmental phenotypes were observed between genotypes (Supplementary Fig. 2b).

**Fig. 2:**
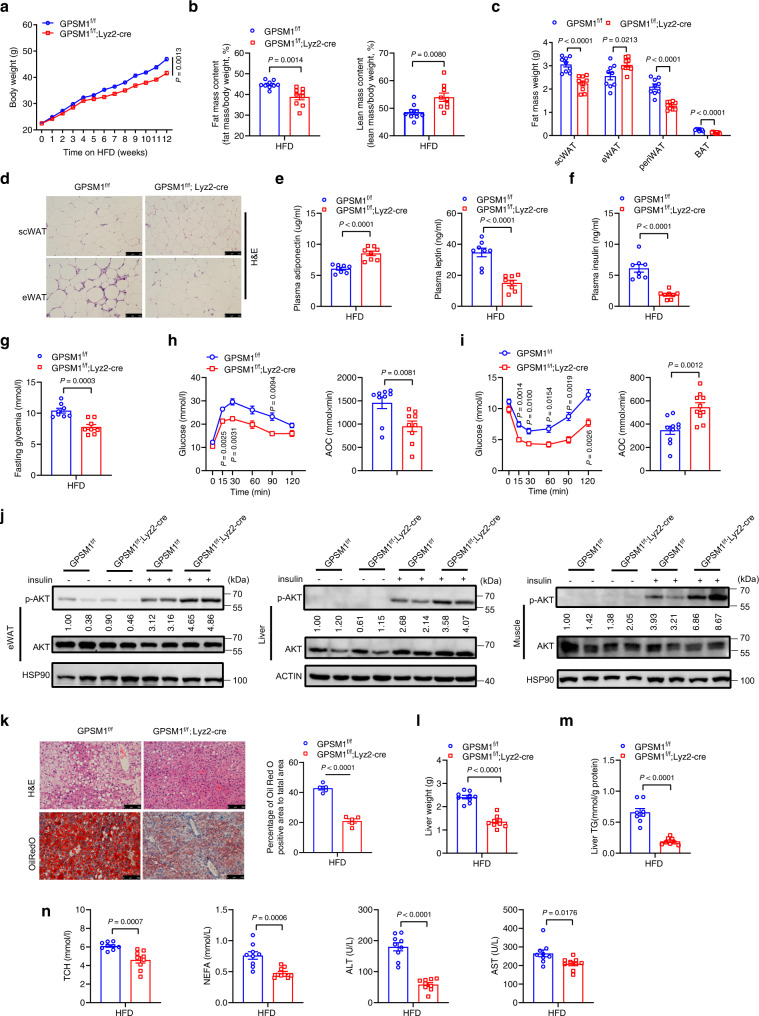
Myeloid GPSM1 abrogation protects mice from diet-induced obesity and metabolic dysfunction. Male *GPSM1*^f/f^; *Lyz2*-cre mice and age-matched *GPSM1*^f/f^ littermates were fed a HFD for 12 weeks. HFD feeding started at 7 weeks of age. **a** Body weight (*n* = 12 biologically independent for *GPSM1*^f/f^ and *n* = 10 biologically independent for *GPSM1*^f/f^; *Lyz2*-cre mice). **b** Percent of fat (left) and lean (right) body mass (*n* = 9 biologically independent mice per group). **c** fat-pad weights (n = 9 biologically independent mice per group). **d** Hematoxylin and eosin (H&E) staining of eWAT and scWAT sections. Scale bars, 100 μm. Independent experiments were repeated three times with similar results. **e** Serum concentration of adiponectin and leptin (*n* = 8 biologically independent mice per group). **f** Serum insulin levels (*n* = 8 biologically independent mice per group). **g** Fasting glucose levels (*n* = 8 biologically independent mice per group). **h** Glucose tolerance test and AOC (area over the curve), *n* = 9 biologically independent mice per group. **i** Insulin tolerance test and AOC (area over the curve), *n* = 10 biologically independent mice per group. **j** Immunoblots of AKT phosphorylation in murine eWAT, liver and muscle after insulin administration (1.5 U/kg) or PBS in vivo. **k** Representative images of H&E staining (top) and Oil Red O (bottom) staining of liver sections and quantification (*n* = 5 biologically independent mice per group). Scale bars, 100 μm. **l** Liver weight (*n* = 9 biologically independent mice per group). **m** Quantification of hepatic triglycerides (*n* = 8 biologically independent mice per group). **n** Serum levels of total cholesterol (TCH), non-esterified fatty acid (NEFA), ALT, and AST (*n* = 9 biologically independent mice per group). All data are shown as means ± SEM. *P* values are determined by two-way analysis of variance (ANOVA) with Sidak’s multiple-comparisons test (**a**, **h**, and **i**) or unpaired two-tailed Student’s *t*-test (**b**, **c**, **e** to **i**, and **k**–**n**).

In the setting of Normal Chow Diet (NCD) feeding for 22 weeks, no significant differences were detected in murine body weight and adiposity between the genotypes (Supplementary Fig. 2c, d). *GPSM1* deficient mice exhibited mildly improved insulin sensitivity (Supplementary Fig. 2e) and diminished lipid accumulation in the liver (Supplementary Fig. 2f–h). At 12 weeks of HFD challenge, the mice lacking *GPSM1* exclusively in myeloid lineage cells were partially resistant to obesity, exhibiting much lower body weights and fat mass without significant changes in their lean mass (Fig. 2a, b and Supplementary Fig. 3a, b), which were demonstrated by dramatic decreases in fat-pad weight, including scWAT, perirenal WAT (periWAT), and brown adipose tissue (BAT) (Fig. 2c and Supplementary Fig. 3c). *GPSM1*^f/f^; *Lyz2*-Cre mice had smaller scWAT adipocyte sizes and similar eWAT adipocyte sizes compared with those of their littermates (Fig. 2d), and the adipocyte numbers of eWAT were much more than those of the control mice (Supplementary Fig. 3d, e), indicating hyperplastic eWAT expansion. All the characteristics above led to a more beneficial metabolic phenotype. HFD *GPSM1* deficiency mice had a 1.4-fold elevation of serum adiponectin level and a 56.6% decrease in hyperleptinemia (Fig. 2e), as well as a 69.7 and a 25.2% decrease in fasting hyperinsulinemia and glucose (Fig. 2f, g), respectively, when compared with the levels in the control mice. Consistently, HFD *GPSM1*^f/f^; *Lyz2*-Cre mice showed greatly improved glucose tolerance and insulin sensitivity (Fig. 2h, i) in addition to increased AKT phosphorylation, which indicated improved intracellular insulin signaling, in eWAT, liver, and skeletal muscle (Fig. 2j and Supplementary Fig. 3f), relative to in HFD *GPSM1*^f/f^ mice.

In addition, we examined the impact of GPSM1 deficiency on lipid metabolism. We observed that *GPSM1*^f/f^; *Lyz2*-Cre mice were obviously protected from HFD-induced hepatic steatosis (Fig. 2k and Supplementary Fig. 3g), with lower liver weights (Fig. 2l) and intra-hepatic triglyceride (TG) contents (Fig. 2m). Consistently, *GPSM1*^f/f^; *Lyz2*-Cre mice indeed had lower serum cholesterol, free fatty acid (FFA), AST, and ALT levels (Fig. 2n). The anti-steatosis effects of myeloid *GPSM1* knockout were presumably attributed to downregulation of fatty acid uptake genes (*CD36*, *FABP4*) and upregulation of fatty acid β-oxidation genes (*LCAD*) in the liver (Supplementary Fig. 3h). In addition, HFD-fed female *GPSM1*^f/f^; *Lyz2*-Cre mice exhibited phenotypic changes similar to those of male mice (Supplementary Fig. 4). Overall, the data demonstrate that GPSM1 in myeloid cells plays an important role in the development of metabolic dysfunction.

### Absence of GPSM1 in myeloid lineage cells alleviates metabolic inflammation

Next, to explore the underlying mechanisms of the antagonizing effects of *GPSM1* ablation on metabolic disorders, we considered whether myeloid GPSM1 might be coupled to metabolic inflammation. As leukocytosis and monocytosis promote metabolic inflammation^19–21^, we first examined whether myeloid GPSM1 loss could affect the proliferation of the Lin^−^Sca1^−^cKit^+^ myeloid progenitors (MPCs) in the bone marrow or blood leukocyte counts and monocyte subset distribution. No significant differences were observed between *GPSM1*^f/f^ and *GPSM1*^f/f^; *Lyz2*-Cre mice both under the NCD and HFD condition (Supplementary Fig. 5a–c).

**Fig. 3:**
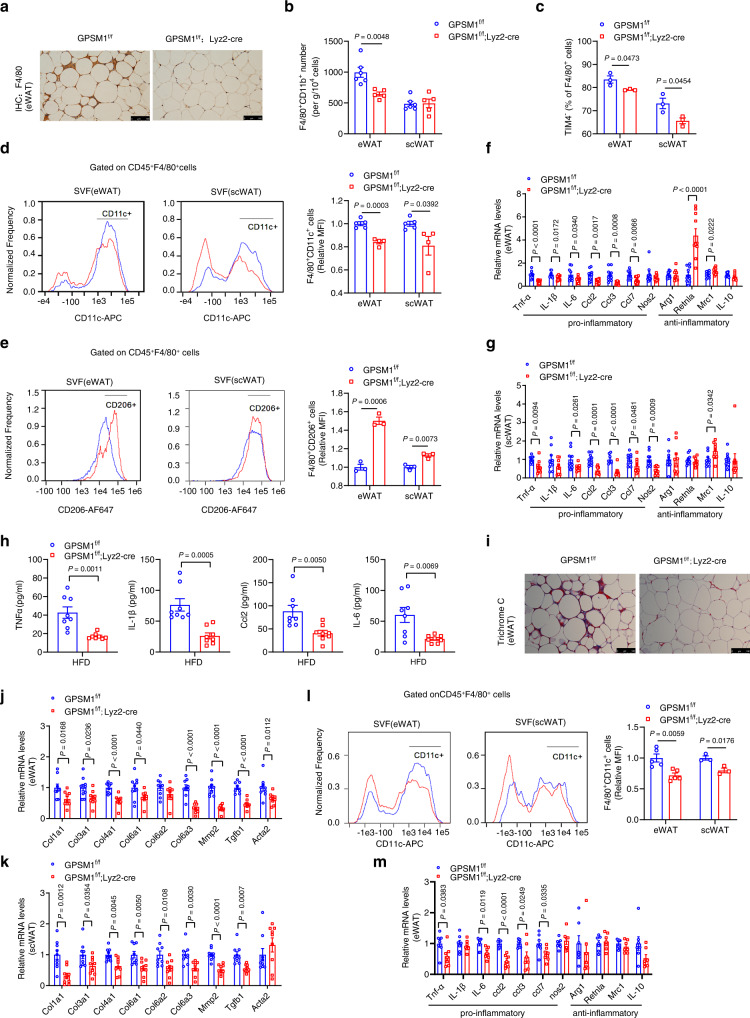
GPSM1 deficiency alleviates metabolic inflammation in HFD-fed mice. **a**–**k** Male *GPSM1*^*f/f*^; *Lyz2*-cre and age-matched *GPSM1*^f/f^ mice were fed a HFD for 12 weeks. **a** Representative F4/80^+^ staining of eWAT sections. Scale bars, 100 μm. **b** Flow cytometry quantification of eWAT and scWAT F4/80^+^CD11b^+^ macrophages from *GPSM1*^f/f^ and *GPSM1*^f/f^; *Lyz2*-cre mice (*n* = 6 biologically independent mice for *GPSM1*^f/f^ and *n* = 5 biologically independent mice for *GPSM1*^f/f^; *Lyz2*-cre). **c** Flow cytometry quantification of TIM4^−^ subset of total macrophages (*n* = 3 biologically independent mice per group). **d** Representative flow cytometry analysis and quantification of the expression of CD11c from CD45^+^F4/80^+^ cells in SVFs from eWAT and scWAT (*n* = 5 biologically independent mice for *GPSM1*^f/f^ and *n* = 4 biologically independent mice for *GPSM1*^f/f^; *Lyz2*-cre). **e** Representative flow cytometry analysis and quantification of the expression of CD206 from CD45^+^F4/80^+^ cells in SVFs from eWAT and scWAT (*n* = 3 biologically independent mice per group). RT-qPCR analysis indicating mRNA abundance of pro-inflammatory and anti-inflammatory genes in eWAT (**f**, *n* = 10 biologically independent mice per group) and scWAT (**g**, *n* = 9 biologically independent mice per group). **h** Serum concentrations of TNF-α, IL-1β, IL-6, and CCL2 (*n* = 8 biologically independent mice per group). **i** Representative Masson’s trichrome staining of eWAT sections. Scale bars, 100 μm. RT-qPCR analysis indicating mRNA abundance of collagen synthesis genes in eWAT (**j**) and scWAT (**k**), *n* = 9 biologically independent mice per group. **l**, **m** Male *GPSM1*^f/f^; *Lyz2*-cre and age-matched *GPSM1*^f/f^ mice were fed a HFD for 5 weeks. **l** Representative flow cytometry analysis and quantification of the expression of CD11c from CD45^+^F4/80^+^ cells in SVFs from eWAT (*n* = 5 biologically independent mice per group) and scWAT (*n* = 3 biologically independent mice per group). **m** RT-PCR analysis indicating mRNA abundance of pro-inflammatory and anti-inflammatory genes in eWAT (*n* = 7 biologically independent mice per group). The experiments were repeated five times with similar results (**a**, **i**). Throughout, data are presented as means ± SEM. *P* values are determined by unpaired two-tailed Student’s *t*-test (**b**–**h**, **j** to **m**).

Then we considered whether myeloid GPSM1 influenced adipose tissue inflammation by mediating a shift in M1–M2 like ATMs. HFD significantly induced F4/80^+^ crown-like structures (CLS) of eWAT in *GPSM1*^f/f^ mice, based on the results of immunohistochemistry staining, the hallmarks of leukocyte and macrophage infiltration, whereas the structures were less observed in *GPSM1*^f/f^; *Lyz2*-Cre mice (Fig. 3a and Supplementary Fig. 5d). Consistently, the F4/80^+^CD11b^+^ cell numbers in eWAT, as measured by flow cytometry, were 35.3% lower, in HFD-fed *GPSM1*^f/f^; *Lyz2*-Cre mice (Fig. 3b and Supplementary Fig. 5e). Further sorting of F4/80^+^TIM4^−^ monocyte-derived ATMs and F4/80^+^TIM4^+^ resident ATMs revealed decreases in the proportions of F4/80^+^TIM4^−^ ATMs, both in the eWAT and scWAT of *GPSM1*^f/f^; *Lyz2*-Cre mice, when compared with in *GPSM1*^f/f^ mice (Fig. 3c and Supplementary Fig. 5f), indicating myeloid *GPSM1* deficiency mainly reduces monocyte-derived macrophage recruitment in WAT. Consistent with the notion that F4/80^+^TIM4^−^ ATMs highly express pro-inflammatory genes and contribute to the expansion of the ATM pool in obesity, ultimately promoting diet-associated inflammation, here, *GPSM1*^f/f^; *Lyz2*-Cre mice actually had fewer F4/80^+^CD11c^+^ pro-inflammatory M1-like ATMs but more F4/80^+^CD206^+^ anti-inflammatory M2-like ATMs, both in the eWAT and scWAT (Fig. 3d, e), confirming a diminished inflammatory environment in *GPSM1*^f/f^; *Lyz2*-Cre mice. Moreover, gene expression profiling also showed robust decreases in the mRNA levels of pro-inflammatory cytokines and chemokines, and slight increases in the mRNA levels of anti-inflammatory markers in WAT of HFD-fed *GPSM1*^f/f^; *Lyz2*-Cre mice, when compared with the levels in their littermates (Fig. 3f, g). Besides diminished adipose tissue inflammation, HFD-fed *GPSM1*^f/f^; *Lyz2*-Cre mice exhibited 59.9, 65.8, 64.6, and 53.7% reductions in the serum levels of pro-inflammatory cytokines Tnf-α, IL-1β, IL-6, and Ccl2, respectively, when compared with the levels in their *GPSM1*^f/f^ counterparts (Fig. 3h), also demonstrating decreased systemic inflammation. Additionally, as accumulative macrophage infiltration causes hypoxia, leading to adipocyte death and eventual fibrosis^22,23^, myeloid *GPSM1* depletion reduced HFD-induced extracellular matrix (ECM) accumulation (Fig. 3i and Supplementary Fig. 5g) and down-regulated the expression of collagen synthesis genes of WAT (Fig. 3j, k), in turn diminishing pathological adipose tissue remodeling.

Subsequently, we assessed whether diminished local and systemic inflammation was causatively related with myeloid *GPSM1* loss or whether it was merely a result of improved glucose and insulin tolerance in the HFD setting for 8–12 weeks. We characterized HFD-fed *GPSM1*^f/f^ and *GPSM1*^f/f^; *Lyz2*-cre mice at 5 weeks, when there was no difference in glucose tolerance and insulin sensitivity (Supplementary Fig. 5h). At the time, *GPSM1*^f/f^; *Lyz2*-cre mice showed significant reductions in F4/80^+^ macrophage infiltration (Supplementary Fig. 5i) and F4/80^+^CD11c^+^ M1-like ATMs proportions (Fig. 3l), in addition to pro-inflammatory marker expression in eWAT (Fig. 3m), when compared with *GPSM1*^f/f^ littermates. The data imply that the effects observed after myeloid *GPSM1* deficiency were of primary cause and not secondary to the improved insulin tolerance, which is consistent with the hypothesis that inflammation can directly cause insulin resistance^24,25^. The finding is also in agreement with the study reported in a Nature Immunology paper that proposed the view that the WAT inflammation precedes the induction of insulin resistance in the HFD-feeding myeloid *SUCNR1* deficiency mice^26^.

Since Lyz2^cre^ has been reported to be expressed both in macrophages and neutrophils^27,28^, we assessed whether the metabolic effects in *GPSM1*;^f/f^ *Lyz2*-cre mice were partially attributed to neutrophils in addition to macrophages. We observed that HFD-fed *GPSM1*^f/f^ and *GPSM1*^f/f^; *Lyz2*-cre mice had similar activity of neutrophil activation marker myeloperoxidase (MPO)^29,30^ in eWAT (Supplementary Fig. 6a) and comparable amounts of neutrophil distinctive inflammatory markers, including S100a8, IL-6, Mmp9, Catalase, and Cxcl1^29^, following sorting of eWAT neutrophils by flow cytometry (Supplementary Fig. 6b). In addition, we blocked CSF1R in *GPSM1*^f/f^ and *GPSM1*^f/f^; *Lyz2*-cre mice, which had already been HFD-feeding for five weeks, to test the possibility that GPSM1 regulation is restricted to macrophages. CSF1R antibody injection reduced the proportion of F4/80^+^CD11b^+^ ATMs by 60% when compared with IgG injection (Supplementary Fig. 6c). As expected, *GPSM1*^f/f^; *Lyz2*-cre mice treated with CSF1R antibody had greater body weights (Supplementary Fig. 6d), and much more impaired glucose and insulin tolerance, when compared with *GPSM1*^f/f^; *Lyz2*-cre mice treated with the IgG isotype (Supplementary Fig. 6e, f). Conversely, they exhibited an obese phenotype comparable with that of *GPSM1*^f/f^ mice injected with CSF1R, suggesting that macrophage depletion could eliminate the improved metabolic characteristics of *GPSM1* deficiency in myeloid cells. Collectively, the results demonstrate that *GPSM1* deletion in macrophages, but not neutrophils, account for the improvements in metabolic inflammation and homeostasis.

### GPSM1 deficiency in myeloid cells increases energy expenditure

**Fig. 4:**
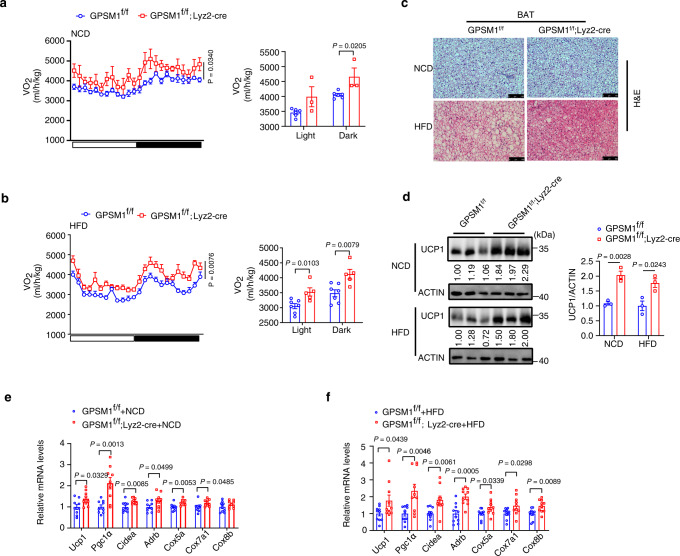
GPSM1 deficiency in macrophages increases energy expenditure. **a**, **b** Oxygen consumption (VO_2_) monitored over a 24-h period and shown as averaged values in NCD-fed (**a**, *n* = 6 biologically independent mice for *GPSM1*^f/f^ and *n* = 3 biologically independent mice for *GPSM1*^f/f^; *Lyz2*-cre) and HFD-fed (**b**, *n* = 7 biologically independent mice for *GPSM1*^f/f^ and *n* = 5 biologically independent mice for *GPSM1*^f/f^; *Lyz2*-cre). **c** Representative images of H&E staining of BAT sections. Scale bars, 100 μm. **d** Immunoblots of UCP1 in BAT and quantification (right), *n* = 3 biologically independent mice per condition. Quantitative RT-PCR analysis of the mRNA abundance of the thermogenic markers in BAT of NCD-fed (**e**, *n* = 9 biologically independent mice per group) and HFD-fed conditions (**f**, *n* = 10 biologically independent mice per group). The experiments were repeated three times with similar results (**c**). Throughout, all data are presented as means ± SEM. *P* values are determined by two-way ANOVA with Sidak’s multiple-comparisons test (**a**, **b**) or unpaired two-tailed Student’s *t*-test (**a**, **b**, **d**–**f**).

Researches have ruled out the critical role of pro-inflammatory or anti-inflammatory ATMs during thermogenesis by influencing adrenergic signaling in adipocytes. We investigated whether myeloid GPSM1 deficiency affects energy balance. Metabolic cage studies showed that *GPSM1*^f/f^; *Lyz2*-Cre mice and their *GPSM1*^f/f^ controls had similar daily food consumption and comparable physical activity during NCD or HFD-feeding (Supplementary Fig. 7a, b); however, oxygen consumption, when normalized to the body weight, was higher in *GPSM1*^f/f^; *Lyz2*-Cre mice than in their *GPSM1*^f/f^ counterparts fed either an NCD or an HFD (Fig. 4a, b). Furthermore, the relative increase in BAT metabolic activity, as determined based on much lower lipid-droplet content (Fig. 4c) and higher UCP1 expression, revealed by Western blot (Fig. 4d), were observed in the BAT of *GPSM1*^f/f^; *Lyz2*-Cre mice, relative to in the control littermates. Consistently, quantitative PCR analysis confirmed that *GPSM1*^f/f^; *Lyz2*-Cre mice had considerably increased levels of expression of key thermogenic markers in BAT (Fig. 4e, f), further suggesting that protection of BAT by myeloid-specific deletion of *GPSM1* is partially responsible for enhanced energy expenditure.

### GPSM1 deficiency inhibits TLR4-induced NF-κB inflammatory signaling in macrophages

**Fig. 5:**
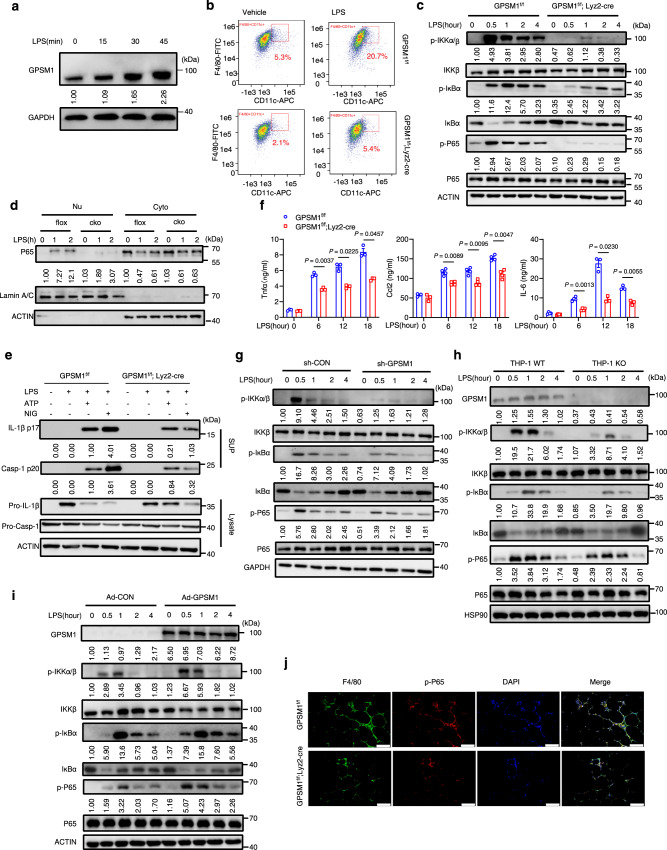
GPSM1 deficiency inhibits TLR4-induced NF-κB inflammatory signaling of macrophages. **a**–**f** Bone marrow-derived macrophages (BMDMs) from *GPSM1*^f/f^; *Lyz2*-cre mice and *GPSM1*^f/f^ littermates were treated with 200 ng/ml LPS or vehicle control for indicated times. **a** Immunoblot analysis of GPSM1 protein in cell lysates of LPS-treated BMDMs. **b** BMDMs were stimulated with LPS for 24 h and levels of F4/80 and CD11c were assessed using flow cytometry. **c** Immunoblot analysis of p-IKKα/β, IKKβ, p-IκBα, IκBα, p-P65, and P65 in BMDMs treated with LPS for indicated times. **d** Immunoblot analysis of nuclear and cytoplasmic extracts and analyzed for P65. **e** LPS-primed BMDMs were treated with ATP or Nigericin. Immunoblotting was used to detect IL-1β, p17, casp-1, and p20 in supernatants (Sup), and pro-IL-1β and pro-casp-1 in cell extracts (lysates). **f** TNF-α, IL-6, and CCL2 levels in culture media were determined using ELISA (*n* = 3 or 4 independent samples per group). **g** *GPSM1*^f/f^ BMDMs were infected with Lv-shCON or Lv-shGPSM1 for 72 h and treated with LPS for additional indicated times. Immunoblot analysis of p-IKKα/β, IKKβ, p-IκBα, IκBα, p-P65, and P65 is shown. **h** Immunoblot analysis of GPSM1, p-IKKα/β, IKKβ, p-IκBα, IκBα, p-P65, and P65 in THP-1 cells treated with LPS (500 ng/ml) for indicated times. **i** Immunoblot analysis of GPSM1, p-IKKα/β, IKKβ, p-IκBα, IκBα, p-P65, and P65 in BMDMs transfected with Ad-CON or Ad-GPSM1 for 48 h and treated with LPS for indicated times. **j** Representative immunofluorescence images of macrophage p-P65 staining with antibodies against p-P65 (red) and F4/80 (green) in eWAT. Nuclei were stained with DAPI (blue). Scale bars, 100 μm. Throughout, all independent experiments were performed three times with similar results (**a**–**e**, **g**–**j**). Data are presented as means ± SEM. *P* values are determined by two-way ANOVA with Sidak’s multiple-comparisons test (**f**).

Having established a critical function of GPSM1 in metabolic deterioration and inducing metabolic inflammation, we investigated the underlying molecular mechanisms. Low doses of lipopolysaccharide (LPS), a well-known pathogen-associated molecule, has been recognized to be involved in obesity pro-inflammatory responses^31^. Therefore, we stimulated mouse BMDMs with LPS to mimic pro-inflammatory status in in vitro models. We found that LPS stimulation could obviously enhance GPSM1 protein level in a time-dependent manner (Fig. 5a), indicating that GPSM1 may affect pro-inflammatory activation in a cell-autonomous fashion. Consistently, flow cytometry analysis showed that LPS-treated *GPSM1*^f/f^; *Lyz2*-Cre BMDMs had a 73.9% decrease in F4/80^+^CD11c^+^ cells, compared with the control *GPSM1*^f/f^ BMDMs (Fig. 5b).

To understand how GPSM1 modulates TLR4-induced pro-inflammatory responses in macrophages under LPS-induced stress, we explored whether GPSM1 influenced the nuclear factor kappa-light-chain-enhancer of activated B cells (NF-κB) signaling pathway, as NF-κB is a key transcription factor inducing pro-inflammatory mediators^32,33^. As expected, loss of *GPSM1* strongly inhibited IKKα-IKKβ phosphorylation and IκBα degradation after LPS stimulation, decreasing P65 phosphorylation, which translocated into the nucleus to initiate the inflammatory cascade (Fig. 5c). We detected reduced nuclear translocation of P65 in *GPSM1*^f/f^; Lyz2-Cre macrophages when compared with *GPSM1*^f/f^ macrophages (Fig. 5d). Furthermore, we compared pro-inflammatory cytokine generation between *GPSM1*^f/f^ and *GPSM1*^f/f^; Lyz2-Cre macrophages. We subjected cell supernatants to immunoblotting and found that *GPSM1*^f/f^; *Lyz2*-Cre BMDMs were less responsive to LPS/ATP or LPS/Nigericin-induced processing of pro-IL-1β into its mature p17 IL-1β form, and pro-caspase-1 into its active p20 form, when compared with the control BMDMs (Fig. 5e). Additionally, Tnf-α, IL-6, and Ccl2 production in response to LPS were all robustly diminished in BMDMs from *GPSM1* deficient mice when compared with in the control mice (Fig. 5f). The above NF-κB signaling pathway was also demonstrated by knockdown experiments using lentiviral short hairpin RNA (shRNA) specific for *GPSM1* (sh*GPSM1*) (Fig. 5g and Supplementary Fig. 8a), as well as THP-1 knockout cells, a widely used human monocyte-like cell line (Fig. 5h). In line with these, adenovirus-mediated *GPSM1-*overexpression in BMDMs obviously promoted the pro-inflammatory responses (Fig. 5i). As obesity progression is associated with high serum FFA levels, and saturated FFAs such as palmitic acid (PA) have greater lipotoxicity^34,35^, we also investigated whether GPSM1 could mediate NF-κB signaling under PA-induced metabolic stress. We found that *GPSM1* deficiency BMDMs had decreased pP65 protein abundance following treatment with PA, demonstrating that the pro-inflammatory effect of GPSM1 on macrophages worked similarly following PA stimulation (Supplementary Fig. 8b).

Finally, we also tested whether GPSM1-mediated NF-κB signaling was involved in the in vivo effects. Immunofluorescence staining results demonstrated that *GPSM1* deficiency also decreased macrophage p-P65 in the mouse eWAT (Fig. 5j and Supplementary Fig. 8c). Overproduction of IL-1β and TNFα, were obviously alleviated in the sorted F4/80^+^ macrophages of eWAT from *GPSM1*^f/f^; *Lyz2*-cre mice (Supplementary Fig. 8d), demonstrating that TLR4-mediated NF-κB signaling was involved in the in vivo effects of myeloid *GPSM1* deficiency. Overall, the results indicate that GPSM1 deficiency or insufficient expression ameliorate pro-inflammatory response by inhibiting the NF-κB signaling pathway in macrophages.

### GPSM1 controls TNFAIP3 and governs pro-inflammatory signaling

**Fig. 6:**
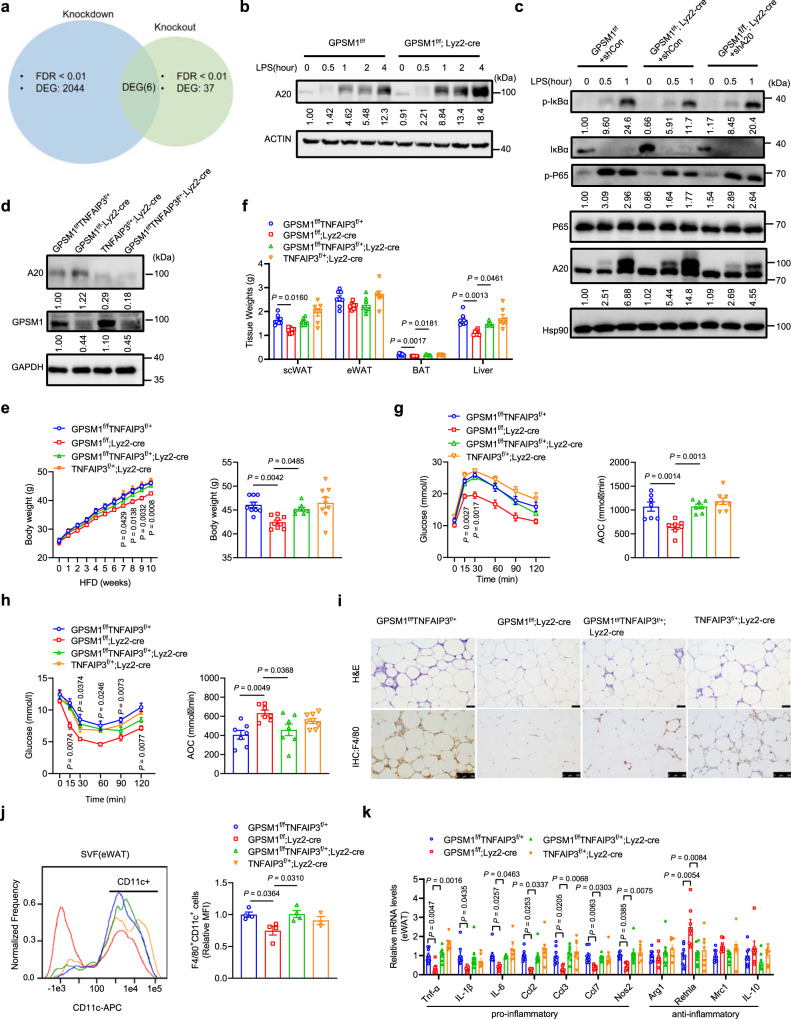
GPSM1 loss inhibits the pro-inflammatory signaling pathway via TNFAIP3. **a** Venn diagram showing overlap of differentially expressed genes from two RNA-sequencing datasets. **b** Immunoblot analysis of A20 in BMDMs isolated from *GPSM1*^f/f^; *Lyz2*-cre and littermates following 200 ng/ml LPS treatment for indicated times. **c** Immunoblot analysis of p-IκBα, IκBα, p-P65, P65, and A20 in *GPSM1*^f/f^ and *GPSM1*^f/f^; *Lyz2*-cre BMDMs infected with indicated lentivirus for 72 h and treated with LPS for additional indicated times. **d**–**k** Metabolic and inflammatory characterization of four genotypes mice fed a HFD for 10 weeks. **d** A20 and GPSM1 protein levels in BMDMs. **e** Body weight curve (left) and the body weight at 10-week HFD (right), *n* = 8 biologically independent mice for *GPSM1*^f/f^*TNFAIP3*^f/+^, *Lyz2*-cre; and *n* = 9 biologically independent mice for other three genotypes, respectively. **f** Tissue weights, including scWAT, eWAT, BAT, and Liver; *n* = 8 biologically independent mice for *GPSM1*^f/f^*TNFAIP3*;^f/+^ *n* = 7 biologically independent mice for other three genotypes, respectively. **g** GTT and AOC; *n* = 8 biologically independent mice for *TNFAIP3*^f/+^, *Lyz2*-cre; and *n* = 7 biologically independent mice for other three genotypes, respectively. **h** ITT and AOC; *n* = 8 biologically independent mice for *TNFAIP3*^f/+^, *Lyz2*-cre; *n* = 7 biologically independent mice for other three genotypes, respectively. **i** H&E (Scale bars, 50 μm) and F4/80^+^ IHC (Scale bars, 100 μm) staining of eWAT. **j** Representative flow cytometry analysis and quantification of the expression of CD11c from CD45^+^F4/80^+^ cells in SVFs from eWAT (*n* = 4 biologically independent mice per group). **k** RT-PCR analysis indicating mRNA abundance of pro-inflammatory and anti-inflammatory genes in eWAT, *n* = 8 biologically independent mice for *GPSM1*^f/f^*TNFAIP3*^f/+^ and *GPSM1*^f/f^*TNFAIP3*^f/+^, *Lyz2*-cre; *n* = 7 biologically independent mice for *GPSM1*^f/f^; *Lyz2*-cre and *TNFAIP3*^f/+^, *Lyz2*-cre. Throughout, all independent experiments were performed three times with similar results (**b**–**d**, **i**). Data are presented as means ± SEM. *P* values are determined by two-way ANOVA with Sidak’s multiple-comparisons test (**e**, **g**, and **h**) or one-way ANOVA with Tukey’s multiple-comparisons test (**e**–**h**, **j**, **k**). For **e**, **g**, and **h**, *P* values are compared with *GPSM1*^f/f^*TNFAIP3*^f/+^ group.

Next, we sought to understand the molecular mechanisms by which GPSM1 modulates the NF-κB signaling pathway in macrophages. To address this issue, we performed RNA-Seq transcriptomic analyses on *GPSM1*^f/f^ versus *GPSM1*^f/f^; *Lyz2*-Cre BMDMs (named as Seq-1) as well as the wild-type BMDMs infected with Lv-shNC versus that infected with Lv-sh*GPSM1* (named as Seq-2) after LPS stimulation, to assess the target genes of GPSM1. Detailed analysis (adjusted *P* < 0.01) showed that Seq-1 identified 37 differentially expressed genes (13 upregulated, 24 downregulated), while Seq-2 identified a total of 2044 transcripts (1102 upregulated, 942 downregulated). Subsequent overlapping of the two datasets revealed six genes (*TNFAIP3*, *CCL22*, *PIK2*, *MAPRE2*, *CH25H*, and *GM43302*) that are commonly regulated by GPSM1, which emphasized a potential critical regulation by GPSM1 on these genes. Among them, *Tnfaip3* was the only gene with the same trend between the two datasets, suggesting it may be a down-stream target of GPSM1 in LPS-induced TLR signaling (Fig. 6a). Subsequently, we validated that TNFAIP3 (namely, A20) protein levels were significantly upregulated in response to LPS in *GPSM1* deficient BMDMs, compared to wild type cells (Fig. 6b). Consistently, *GPSM1*^f/f^; *Lyz2*-cre mice also had a ~90% increase in the mRNA abundance of A20 in the sorted F4/80^+^ ATMs of eWAT in the HFD setting (Supplementary Fig. 9a).

Thus, we first explored whether A20 functions as a potential GPSM1 target gene for mediating the NF-κB pathway in macrophages. KD of *A20* reversed the alleviated pro-inflammatory signaling in the *GPSM1*-KO cells partially, as shown by increased IκBα and p65 protein phosphorylation (Fig. 6c). RT-PCR also revealed that the decreased expression of genes related to pro-inflammatory state in the *GPSM1* KO cells were reversed by *A20* KD (Supplementary Fig. 9b). To further confirm GPSM1 regulation of adipose tissue inflammation via A20 in vivo, we generated *GPSM1*/*TNFAIP3* myeloid-specific double KO mice by crossing *GPSM1*-flox/*TNFAIP3*-flox/+ mice with *Lyz2*-cre mice. Western blot analysis revealed that *A20* was specifically downregulated in macrophages (Fig. 6d and Supplementary Fig. 9c). The body weight (Fig. 6e), fat-pads weights, and liver weight (Fig. 6f) of *GPSM1*^f/f^/*TNFAIP3*^f/+^; *Lyz2*-cre mice were comparable with those of the *GPSM1*^f/f^/*TNFAIP3*^f/+^ mice, but significantly higher than those of *GPSM1*^f/f^; *Lyz2*-cre mice upon HFD feeding for 8 weeks. Consistently, heterozygous *TNFAIP3* depletion reversed the improved glucose metabolic abnormalities and insulin resistance resulting from *GPSM1* deficiency, as revealed by glucose-tolerance tests and insulin-tolerance tests (Fig. 6g, h). Remarkably, the numbers of infiltrated ATMs (Fig. 6i and Supplementary Fig. 9d, e), the frequency of F4/80^+^CD11c^+^ pro-inflammatory M1-like ATMs (Fig. 6j) and the expression of pro-inflammatory cytokines of the *GPSM1*^f/f^/*TNFAIP3*^f/+^; Lyz2-cre mice (Fig. 6k) were also similar to those of *GPSM1*^f/f^/*TNFAIP3*^f/+^ mice, demonstrating that *TNFAIP3* downregulation could abolish the inhibitory effect of *GPSM1* deficiency in myeloid cells on adipose tissue inflammation. Collectively, our data suggest that the anti-inflammatory effects of *GPSM1* loss in myeloid cells are mainly due to the influence of A20.

### GPSM1 deficiency-induced TNFAIP3 upregulation is via the Gα_i3_/cAMP/PKA/CREB axis

**Fig. 7:**
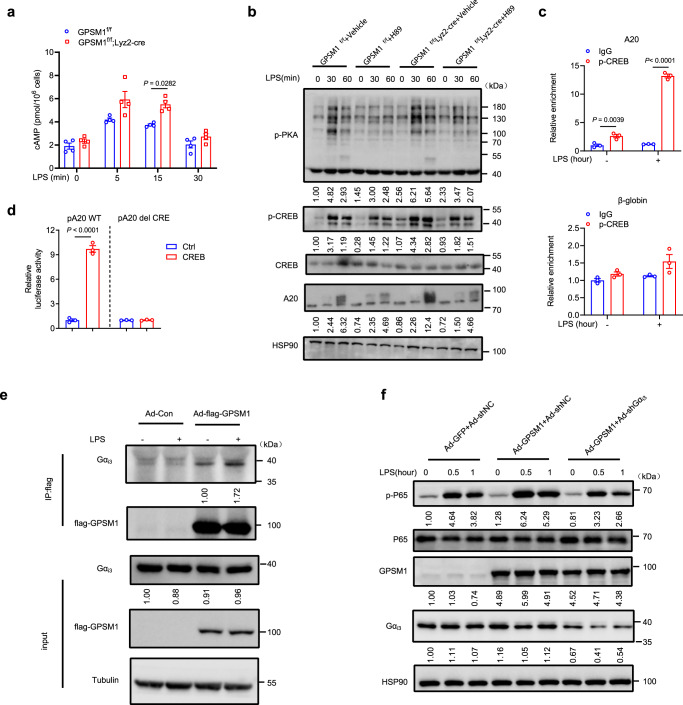
GPSM1 deficiency-induced TNFAIP3 upregulation is via the Gαi3/cAMP/PKA/CREB axis. BMDMs from *GPSM1*^f/f^; *Lyz2*-cre and *GPSM1*^f/f^ mice were treated with 200 ng/ml LPS or vehicle control for indicated times. **a** cAMP levels in BMDMs treated with LPS (*n* = 4 independent samples per group). **b** *GPSM1*^f/f^ and *GPSM1*^f/f^; *Lyz2*-cre BMDMs were pre-treated with H-89 (50 μM) or vehicle for 30 min and then stimulated with LPS for additional indicated times. Immunoblots for p-PKA substrate, p-CREB, CREB and A20 in BMDMs. **c** ChIP-qPCR of A20 in BMDMs with or without LPS stimulation (*n* = 3 independent samples per condition). β-globin was as a control. **d** Luciferase activity of a WT TNFAIP3 promoter reporter (TNFAIP3-Luc) or of a mutant-TNFAIP3 reporter (TNFAIP3-Mut-Luc) containing a deletion in the CREB-responsive element in HEK293T cells transiently expressing either vector (pRL-TK) or CREB (*n* = 3 independent samples per condition). **e** IP of flag from BMDMs transfected with Ad-flag-GPSM1 or Ad-Con, followed by immunoblot analysis of the interaction of GPSM1 with Gα_i3_ under LPS stimulation or not. **f** Immunoblot analysis of p-P65, P65, GPSM1, and Gα_i3_ in BMDMs infected with Ad-GPSM1 or Ad-GFP, and Ad-shGα_i3_ or Ad-shNC for 72 h and treated with LPS for indicated times. Independent experiments were performed three times with similar results (**b**, **e**, and **f**). All data are presented as means ± SEM. *P* values are determined by two-way ANOVA with Sidak’s multiple-comparisons test (**a**), or two-tailed Student’s *t*-test (**c**, **d**).

To understand how GPSM1 exerts its effects on TNFAIP3 transcription, we further investigated the potential molecular link between GPSM1 and TNFAIP3. As GPSM1 was implicated in regulating cyclic AMP (cAMP)—protein kinase A (PKA) activity in the addiction model of the brain^36,37^, we, therefore, explored whether GPSM1 modulated a pro-inflammatory pathway through cAMP/PKA signaling. First, we found that *GPSM1*abrogation indeed increased cAMP levels post-LPS stimulation for 5 and 15 min, compared to *GPSM1*^f/f^ BMDMs (Fig. 7a). In line with this, short-term exposure of *GPSM1*^f/f^ BMDMs to LPS induced the phosphorylation of PKA and PKA-dependent downstream cyclic AMP response element-binding protein (CREB)^38^, an effect that was further enhanced in *GPSM1*^f/f^; Lyz2-Cre BMDMs, but abolished by the selective PKA inhibitor H-89 treatment (Fig. 7b). These results suggested that GPSM1 acted synergistically with LPS to regulate cAMP/PKA/CREB signaling in primary macrophages. We further performed chromatin immunoprecipitation (ChIP) assays and confirmed the direct binding of p-CREB to the *TNFAIP3* promoter, especially upon LPS stimulation (Fig. 7c). The luciferase assay also demonstrated that CREB induced *TNFAIP3* transcription, whereas mutation in the putative CREB binding site abolished transcription activation, supporting the idea that *TNFAIP3* is a transcription target of CREB (Fig. 7d). The addition of H-89 completely reversed the upregulation of A20 in *GPSM1*^f/f^; Lyz2-Cre cells compared to control cells (Fig. 7b), suggesting that the effect of GPSM1 on A20 was PKA dependent. GPSM1 is thought to regulate G protein signaling in the brain nuclei^37^. We explored whether GPSM1 coupled with a specific G protein to facilitate cAMP inhibition and subsequent NF-κB signaling activation following LPS stimulation in BMDMs. We found that Gα_i3_ indeed coimmunoprecipitated and colocalized predominantly with GPSM1 in primary macrophages, and this binding ability was further strengthened upon LPS stimulation (Fig. 7e), which was confirmed by immunoblot analysis, where Gα_i3_ KD blocked GPSM1 overexpression-induced P65 phosphorylation (Fig. 7f). Consequently, we identify the Gα_i3_/cAMP/PKA/CREB/A20 axis as molecular components preferentially regulate GPSM1-induced pro-inflammatory pathway in BMDMs.

### Macrophage GPSM1-driven inflammatory signal regulates adipocyte insulin action

**Fig. 8:**
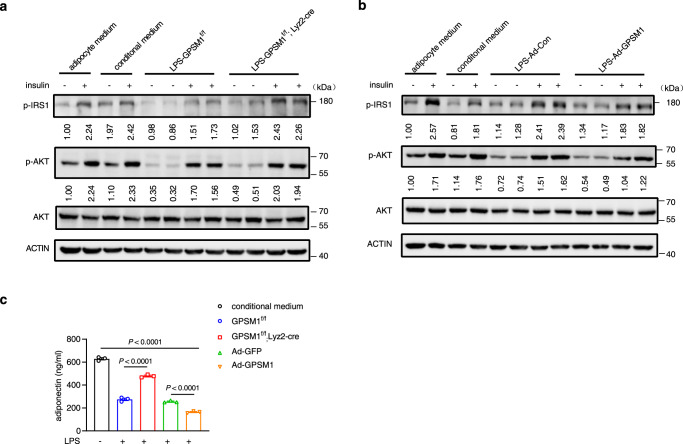
Macrophage GPSM1-driven inflammatory signal regulates co-cultured primary adipocytes insulin action and sensitivity. **a** Primary adipocytes were treated with conditioned media (CM) from LPS-primed *GPSM1*^f/f^ or *GPSM1*^f/f^; *Lyz2*-Cre BMDMs, vehicle-treated BMDMs, and control adipocyte medium (fresh media), for 48 h. Protein expression levels of insulin signaling cascades, p-IRS1, p-AKT, and AKT after insulin (100 nM) or vehicle stimulation for 15 min were assessed using immunoblotting. **b** Primary adipocytes were treated with conditioned media (CM) from LPS-primed *GPSM1*- or Con-overexpressing BMDMs, vehicle-treated BMDMs, and control adipocyte medium (fresh media), for 48 h. Protein expression levels of insulin signaling cascades, p-IRS1, p-AKT, and AKT after insulin (100 nM) or vehicle stimulation for 15 min were assessed using immunoblotting. **c** Adiponectin levels in culture media were determined using ELISA (*n* = 3 independent samples per condition). Independent experiments were performed three times with similar results (**a**, **b**). All data are presented as means ± SEM. *P* values are determined by one-way ANOVA with Tukey’s multiple-comparisons test (**c**).

It has been well established that cytokines produced from macrophages could affect adipocyte function^39^. Therefore, we speculated that macrophage GPSM1 may modulate insulin signaling and the sensitivity of primary adipocytes. To address this, conditioned media (CM) from LPS-primed *GPSM1*^f/f^ or *GPSM1*^f/f^; *Lyz2*-Cre BMDMs, and LPS-primed *GPSM1*- or CON-overexpressing BMDMs, were collected and used to treat differentiated primary adipocytes. Compared with adipocyte medium or CM from vehicle-treated BMDMs, CM from LPS-treated *GPSM1*^f/f^ BMDMs showed the insulin-stimulated IRS1 and phosphorylation of AKT suppression effects, whereas CM from *GPSM1*^f/f^; *Lyz2*-Cre BMDMs had more limited effects (Fig. 8a). Similarly, *GPSM1*-overexpression-CM upon LPS stimulation significantly impaired the insulin action of adipocytes, whereas the inhibitory effects from CON-overexpression CM were also alleviated (Fig. 8b). Moreover, CM from *GPSM1* loss macrophages had a less suppressive effect on adiponectin secretion; conversely, the inhibitory effects were more pronounced in the CM group from *GPSM1*-overxpressing macrophages (Fig. 8c). Overall, the results indicate that macrophage GPSM1 regulates the TLR4-induced NF-κB signaling pathway, which, in turn, influences adipocyte function.

### Identification of potential GPSM1 inhibitors

Subsequently, we identified small-molecule compounds that could counter GPSM1 function and thereby alleviate macrophage inflammation. We used a hybrid strategy combining computational and experimental approaches to screen potential GPSM1 Inhibitors. First, we speculated that there were potential allosteric sites for GPSM1 inhibition. We obtained one potential allosteric site in the AlphaFold predicted structure of GPSM1^40,41^ using the AlloSitePro method^42^. Based on the predicted site, we performed high-throughput virtual screening (HTVS) with 270,000 compounds from the SPECS library. Afterward, we selected 93 compounds on the basis of the top-ranked GPSM1–compound binding modes.

**Fig. 9:**
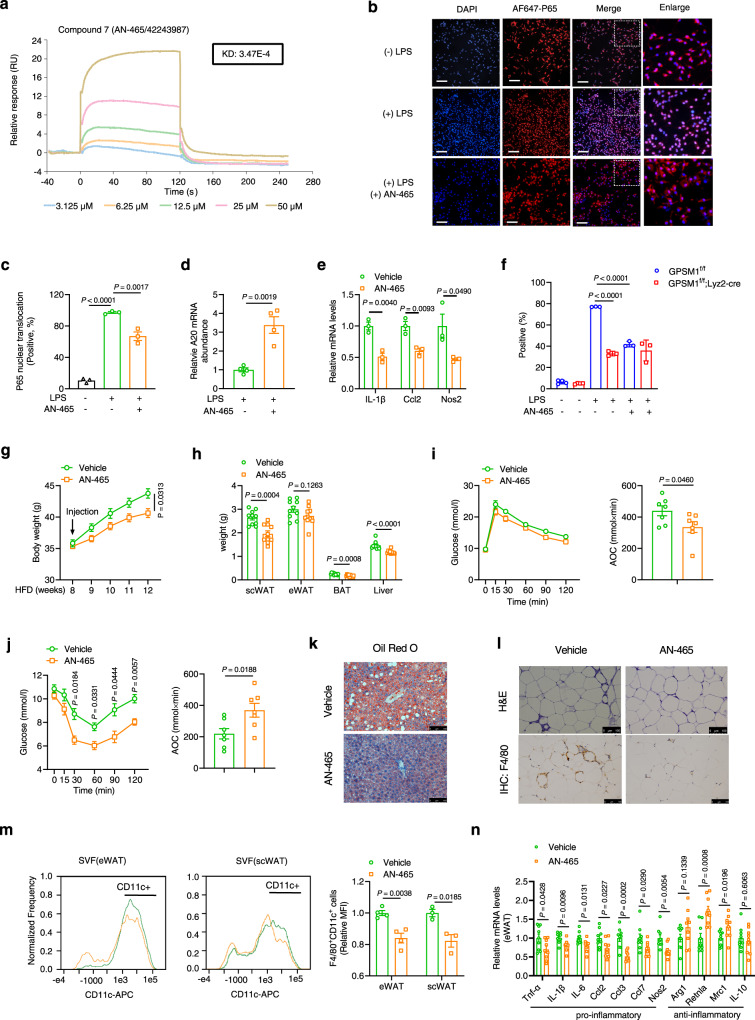
Identification of potential GPSM1 inhibitors. **a** SPR assay with Biacore diagram and saturation curve of AN-465/42243987 binding to GPSM1 protein. As AN-465/42243987 concentration increases, the chip RU continues to increases. **b**–**f** BMDMs treated with 50 μM AN-465/42243987 or vehicle control for 16 h and then stimulated with LPS or not. **b** Representative immunofluorescence images indicating P65 nuclei translocation showed by high-content screen. BMDMs were stained for P65 (red) and DAPI (blue). Scale bars, 100 μm. **c** Quantification of the proportion of P65 nuclear translocation exhibited in (**b**) (*n* = 3 independent samples per condition). **d** The mRNA abundance of A20 in BMDMs after LPS stimulation for 20 min (*n* = 4 independent samples per condition). **e** The mRNA abundance of pro-inflammatory markers in BMDMs stimulated with LPS for 3 h (*n* = 3 independent samples per condition). **f** The effect of AN-465/42243987 on P65 translocation in *GPSM1*^f/f^ and *GPSM1*^f/f^; *Lyz2*-cre BMDMs (*n* = 3 or 4 independent samples per condition). Positive% was defined as the index (P65 intensity in nuclei/cytoplasm) > 1.5. **g**–**n** In vivo effects of AN-465/42243987. DIO mice (already HFD-fed for 8 weeks) were peritoneally administered with either 0.5 mg/kg AN-465/42243987 or vehicle, twice per week for 4 weeks. **g** Body weights (*n* = 10 biologically independent mice per group). **h** Tissue weights (*n* = 10 biologically independent mice per group). **i** GTT and AOC (*n* = 7 biologically independent mice per group). **j** ITT and AOC (*n* = 7 biologically independent mice per group). **k** Oil Red O staining. Scale bars, 100 μm. **l** H&E and F4/80^+^ IHC staining of eWAT. Scale bars, 100 μm. **m** Representative flow cytometry analysis and quantification of the expression of CD11c from CD45^+^F4/80^+^ cells in SVFs from eWAT (*n* = 4 biologically independent mice per group) and scWAT (*n* = 3 biologically independent mice per group). **n** RT-PCR analysis indicating mRNA abundance in eWAT (*n* = 9 biologically independent mice per group). Independent experiments were performed three times with similar results (**b**, **k**, **l**). All data are presented as means ± SEM. *P* values are determined by unpaired two-tailed Student’s *t*-test (**d**, **e**, **h** to **j**, **m**, and **n**), or the two-way ANOVA with Sidak’s multiple-comparisons test (**g**, **i**, and **j**) or one-way ANOVA with Tukey’s multiple-comparisons test (**c**).

To evaluate the activity of the compounds on GPSM1, Biacore analysis was first performed to verify the interaction between GPSM1 protein and small-molecule compounds. Among 93 compounds, 10 representative small molecules were finally selected (Fig. 9a and Supplementary Fig. 10a, b). The docking poses of the 10 small molecules are shown (Supplementary Fig. 10c). Further high-content screening (HCS) revealed that two compounds (compound 6 and compound 7) could largely suppress the pro-inflammatory phenotype revealed by reduced P65 nuclei translocation in wild type BMDMs (Fig. 9b, c and Supplementary Fig. 11a, b); however, only compound 7 (AN-465/42243987) upregulated A20 expression (Fig. 9d and Supplementary Fig. 11c), and qPCR analysis also confirmed that AN-465/42243987 blunted LPS-induced expression of a panel of signature genes: IL-1β, Ccl2 and Nos2 (Fig. 9e). As the two bulky aromatic rings of AN-465/42243987 were favored in the hydrophobic cavity formed via Val340, Pro345, Leu378, and Val381, while its right proportion established four hydrogen bonds with Trp333, Asn337, and Asn374. Therefore, the overall structures of AN-465/42243987 could be stabilized in the allosteric site (Supplementary Fig. 10c). In addition, after depletion of *GPSM1*, AN-465/42243987 treatment could no longer inhibit P65 nuclei translocation (Fig. 9f), suggesting that AN-465/42243987 exerted its role dependent on GPSM1. All the results indicate that AN-465/42243987 may inhibit NF-κB signaling by actually countering GPSM1 function.

Further, we evaluated the potential therapeutic effects of AN-465/42243987 in treating metabolic dysfunction in vivo. DIO mice (already HFD-feeding for 8 weeks) were peritoneally administered with either AN-465/42243987 or vehicle, twice a week for 4 weeks. Compared with the vehicle controls, DIO mice treated with AN-465/42243987 gained less weight (Fig. 9g). The tissue weights, including scWAT, BAT, and liver all obviously decreased after AN-465/42243987 treatment (Fig. 9h). DIO mice treated with AN-465/42243987 had both improved systemic insulin sensitivity and glucose tolerance, as demonstrated by insulin and glucose tolerance tests (Fig. 9i, j). Similarly, Oil red O staining showed that AN-465/42243987 could alleviate HFD-induced hepatic steatosis (Fig. 9k and Supplementary Fig. 11d). Notably, as AN-465/42243987 counters GPSM1 pro-inflammatory function, we observed that AN-465/42243987 treatment reduced the F4/80^+^ crown-like structures (Fig. 9l and Supplementary Fig. 11e), as well as the proportion of F4/80^+^CD11c^+^ pro-inflammatory macrophages (Fig. 9m), compared with the vehicle treatment. Similarly, the expression of genes involved in promoting adipose tissue inflammation were all decreased in the mice injected with AN-465/42243987 (Fig. 9n). In addition, AN-465/42243987 did not cause cytotoxicity or cell apoptosis in vitro (Supplementary Fig. 11f), and serum alanine transaminase and aspartate transaminase concentrations exhibited lower in vivo (Supplementary Fig. 11g), indicating the biosafety of AN-465/42243987. Overall, the results suggest that the small-molecule compound we identified, AN-465/42243987, may have potential therapeutic effects on metabolic disorders in murine models.

### Human adipose tissue GPSM1 is correlated with metabolic quantitative traits

**Fig. 10:**
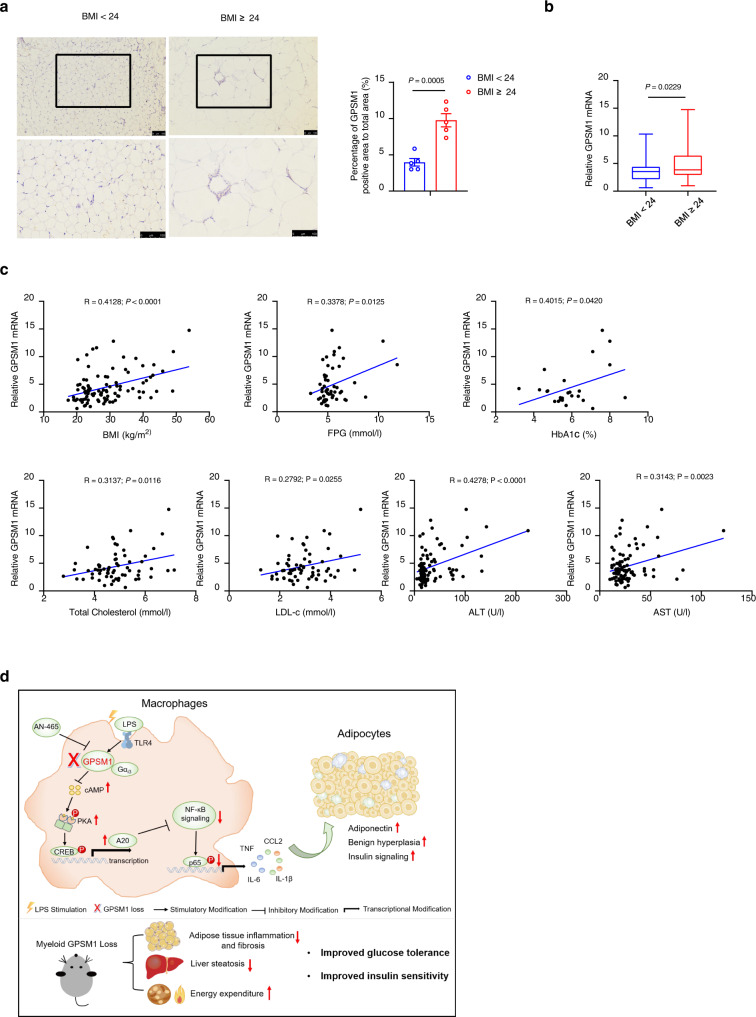
Human adipose tissue GPSM1 is correlated with obesity and metabolic quantitative traits. **a** IHC staining of GPSM1 in visceral fat from individuals without/with overweight or obesity (*n* = 5 biologically independent individuals per group). Scale bars, 100 μm (top) and 50 μm (bottom). Quantification is shown (right). **b** qPCR analysis indicating expression of GPSM1 in visceral fat from individuals without/with overweight or obesity (BMI < 24, *n* = 36 biologically independent individuals; BMI ≥ 24, *n* = 61 biologically independent individuals). *GPSM1* mRNA level was normalized to *RPLP0* mRNA. **c** Correlation between *GPSM1* mRNA expression in visceral fat with clinical metabolic traits, including BMI (*n* = 97 biologically independent individuals), Fasting Plasma Glucose (FPG, *n* = 54 biologically independent individuals), HbA1c (*n* = 26 biologically independent individuals), Total cholesterol (*n* = 64 biologically independent individuals), LDL-c (*n* = 64 biologically independent individuals), ALT (*n* = 92 biologically independent individuals), and AST (*n* = 92 biologically independent individuals) from human subjects. **d** Model of how macrophage GPSM1 controls TLR-induced NF-κB signaling and pro-inflammatory cytokine secretion and thus influence adipocyte insulin sensitivity and function. Mice with myeloid-specific *GPSM1* ablation displayed ameliorated adipose inflammation, liver steatosis, and improved systemic glucose tolerance, insulin sensitivity, and energy expenditure upon HFD challenge. Mechanically, upon LPS stimulation, macrophage *GPSM1* loss could upregulate *TNFAIP3* transcription via the Gα_i3_/cAMP/PKA/CREB axis, thereby inhibiting NF-κB signaling. One small-molecule compound, AN-465, targeting GPSM1, would be a candidate for metabolic therapy. Data are presented as means ± SEM (**a**), or boxplots represent the median value, with lower and upper hinges corresponding to the 25th and 75th percentiles, and lower and upper whiskers extending from the hinge to the smallest and largest value (**b**). *P* values are determined by unpaired two-tailed Student’s *t*-test (**a**, **b**), Pearson’s correlation analysis showing *R* values, and two-tailed *P* values (**c**).

To further investigate whether GPSM1 expression is also increased in human obesity, we analyzed its expression in visceral fat biopsies of a cohort of individuals with a wide body mass index (BMI) range (Supplementary Table 1). IHC staining and qPCR analysis revealed that expression of GPSM1 was substantially higher in visceral fat from the subjects with overweight or obesity (BMI ≥ 24) than that from the subjects with normal BMI (BMI < 24, Fig. 10a, b). Notably, we observed a positive correlation of expression of *GPSM1* in visceral fat with clinical quantitative traits indicating obesity and glucose metabolism, such as BMI, Fasting plasma glucose (FPG), HbA1c, as well as biochemical indicators, including TC, LDL-c, ALT, and AST (Fig. 10c). Overall, together with the observations that GPSM1-deficient mice were protected from developing diet-induced obesity and glucose and lipid dysregulation, our results strongly suggest that increased GPSM1 expression in human visceral fat may be causally linked to the pathogenesis of obesity, T2D, and metabolic disturbances.

## Discussion

Herein, we elucidate a completely function of T2D susceptible gene *GPSM1*, and the specific molecular mechanism mediated by GPSM1 in the pro-inflammatory pathway in macrophages. During over-nutrition, obesogenic factors, such as LPS and free fatty acids, activate macrophage GPSM1 to trigger the IKK-NF-κB signaling cascade and produce pro-inflammatory cytokines. The deleterious changes in adipose tissue microenvironments further impair insulin action to exacerbate systemic insulin resistance, ultimately leading to metabolic dysfunction. Mechanistically, GPSM1 governs *TNFAIP3* transcription to regulate the TLR-induced NF-κB pathway via the Gα_i3_/cAMP/PKA/CREB axis. Of clinical significance, *GPSM1* level is increased in the visceral fat of individuals with overweight or obesity, so that it is a potential therapeutic target for metabolic disorders.

Chronic low-grade adipose tissue inflammation is well characterized by CCR2-dependent pro-inflammatory macrophage recruitment and resident ATMs reprogrammed from a homeostatic into a pro-inflammatory sate. Macrophages are considered the most essential cells for pro-inflammatory cytokine production. Sustained inflammatory environments within adipose tissues cause adipocyte death and eventual metabolic deterioration^11,26^. In the present study, we demonstrate that mice with genetic ablation of *GPSM1* in myeloid cells exhibit alleviated local eWAT inflammation and fibrosis in response to caloric excess. The systemic metabolic phenotypes in our *GPSM1*^f/f^; *Lyz2*-Cre mice are consistent with the view that the health and functionality of WAT influences systemic metabolic homeostasis^43^, ensuring safe storage of energy in the WAT and preventing lipotoxicity in peripheral tissues^44,45^. Although the notion that shifting adipose tissue expansion from hypertrophy to hyperplasia may prevent pathological remodeling and adipose dysfunction has largely been derived from studies of subcutaneous adipose tissue^43,46^, while our data suggest that the health of visceral adipocytes also has an effect, which is consistent with the recent findings reported by Senol et al. and Shao et al.^47,48^. In addition, the phenotype of *GPSM1*^f/f^; *Lyz2*-Cre mice is consistent, but somewhat less prominent, when compared with that of the global *GPSM1*^−/−^ mice, in which there are significant body weight and fat mass reductions, as well as energy expenditure enhancement, under chow diet conditions^49^. The findings suggest that other sources of GPSM1 may also contribute to the development of metabolic dysregulation. GPSM1 is reportedly highly expressed in the hypothalamus and brain^50,51^. The roles of other tissue sources of GPSM1 in obesity warrant further investigation.

Previous studies have reported that GPSM1 is involved in chemokine-induced migration and motility of B and T lymphocytes and bone marrow-derived dendritic cells^8^. As recruitment of monocyte-derived macrophage may account for adipose tissue inflammation, here, we also identify that myeloid *GPSM1* loss could impair the responsiveness of monocytes to chemoattractants both in in vivo and in vitro experiments (Supplementary Fig. 12). Nevertheless, notably, in the present study, GPSM1 is a prominent intrinsic pro-inflammatory gatekeeper of macrophages. We demonstrate that myeloid *GPSM1* loss has a pronounced inhibitory effect on TLR-induced NF-κB signaling by upregulating *TNFAIP3* transcription through the Gα_i3_/cAMP/PKA/CREB axis in macrophages. As impaired macrophage inflammatory signaling could further reduce the transfer of blood monocytes into adipose tissue, we propose that myeloid *GPSM1* deficiency inhibits metabolic inflammation via two coordinated mechanisms. The coordinated cellular events highlight what often happens to other immune molecules such MVP^52^.

Notably, we propose that the regulation of *TNFAIP3* expression is the primary molecular mechanism of the physiological function of macrophage GPSM1 in inflammation. TNFAIP3 plays a central role in TLR signaling and serves as a cytoplasmic ubiquitin-modifying enzyme that deubiquitinates K63 of TRAF6 to terminate NF-κB-driven inflammatory signals^53,54^. Our study demonstrated that knockdown of myeloid *TNFAIP3* reversed the beneficial effects of GPSM1 abrogation on inflammation both in vitro and in vivo. As GPSM1 is a cytoplasmic protein, it is expected that no direct transcriptional interaction exists between GPSM1 and TNFAIP3. Considering GPSM1 is an accessory protein that could activate G-protein and may influence its downstream cAMP/PKA pathway, in addition to the hypothesis that the cAMP/PKA pathway may be involved in inflammatory signaling^55,56^, we speculated that the cAMP/PKA/CREB pathway may mediate the regulatory effects of GPSM1 on TNFAIP3. As expected, macrophage GPSM1 loss activates the cAMP/PKA/CREB pathway by less binding to Gα_i3_; subsequently, phosphorylation of CREB promotes *TNFAIP3* transcription. As a consequence, the increase of TNFAIP3 represses NF-κB signaling. Although the role of the cAMP/PKA/CREB pathway has been well identified in adipocyte thermogenesis^57^, here, we demonstrate that p-CREB could upregulate *TNFAIP3* transcription in macrophages, especially under inflammatory states.

Anti-inflammation is a key strategy for the treatment of metabolic diseases^58,59^. ATMs have been considered therapeutic targets for their crucial role in metabolic inflammation and related dysfunctions. In the present study, we characterize a small-molecule compound, AN-465/42243987, which specifically binds to the allosteric site and inhibits macrophage GPSM1 function, and suppresses local and systemic inflammation to ameliorate metabolic deterioration in DIO mice. Peritoneal administration enables the direct physical delivery of doses to visceral adipose tissue, and the fewer blood vessels and slower flow in adipose tissue are expected to facilitate longer stay of the administered dose in the visceral adipose tissue^60^. The compound has promising clinical applications.

One limitation of this study is that the overall effects of myeloid-restricted *GPSM1* deficiency seem modest. As GPSM1 is constitutively manipulated in macrophages, Kupffer and microglia cells are also impacted. It suggests that these sources of GPSM1 may have diverse effects and also contribute to the development of metabolic dysregulation. The underlying mechanisms is needed to investigate in future studies. Besides, the improvement of metabolic phenotype of *GPSM1*^f/f^; Lyz2-cre females compared with the counterparts, is weaker than males. As *GPSM1* knockdown had downregulated trend of *ESRRA* mRNA expression in our RNA-Seq data (NODE: OEP003692), and the estrogen receptor signaling is known to confer systemic protective metabolic effects in the context of obesity^61,62^, thus, we speculate that estrogen signaling in females may in part underlie the observed sex difference. More molecular mechanisms need further investigation.

In summary, the present study identified GPSM1 as a node linking inflammatory responses of macrophages and metabolic dysregulation via the Gα_i3_/cAMP/PKA/CREB/A20 axis. Our findings enhance our understanding of immunometabolism and identify possible targets for therapy against T2D, obesity, and associated metabolic disturbances.

## Methods

### Human tissue samples

All individuals gave informed consent and the study was approved by the Human Research Ethics Committee of Shanghai Sixth People’s Hospital affiliated to Shanghai Jiao Tong University School of Medicine. Height and weight were measured using a standard scale, with BMI (kg/m^2^) calculated from two averaged measurements. Donors were classified as normal weight (BMI < 24) or overweight and obesity (BMI ≥ 24) based on BMI according to the Working Group on Obesity in China criteria. Visceral fat was obtained from age and gender-matched human donors undergoing scheduled routine surgery. Anthropometric and biochemical traits are presented in Supplementary Table 1 (Table S1). Tissue biopsies were stored at −80 °C until further processing. To examine GPSM1 expression, paraffin sections were stained with an anti-GPSM1 antibody (11483-1-AP, Proteintech, 1:50).

### Mice, diets, and treatments

C57BL/6J mice were purchased from GemPharmatech Co. Ltd. Lyz2-cre mice, which express the Cre recombinase transgene under the control of the lysozyme 2 gene promoter/enhancer elements, were purchased from the Jackson Laboratory (Stock No. 004781). *GPSM1*^f/f^ mice were generated under the C57BL/6J genetic background by GemPharmatech Co. Ltd, in which exon 2–11 of the GPSM1 allele was flanked by *lox*P sites. Myeloid cell-specific *GPSM1* knockout mice (*GPSM1*^f/f^; *Lyz2*-Cre) were subsequently produced via intercrossing *GPSM1*^f/f^ mice with heterozygous *Lyz2*-Cre mice. Myeloid cell-specific *TNFAIP3* heterozygous KO (*TNFAIP3*^f/+^; *Lyz2*-cre) mice were generated by mating *TNFAIP3*^flox/+^ mice with heterozygous *Lyz2*-cre mice. Myeloid-cell specific *GPSM1* KO *TNFAIP3* heterozygous KO mice (*GPSM1*^f/f^*TNFAIP3*^f/+^; *Lyz2*-cre) mice were generated by mating *GPSM1*^f/f^; *TNFAIP3*^f/+^mice with heterozygous *Lyz2*-cre mice. *TNFAIP3*^flox/+^ mice were kindly provide by Dr. Yang Xiao and Dr. Shanshan Liu (The Second Xiangya Hospital of Central South University). Mice were genotyped by PCR using DNA isolated from tails. The primers are showed in Supplementary Table 2 (Table S2).

*GPSM1*^f/f^ and *GPSM1*^f/f^ *Lyz2*-cre mice already HFD-feeding for 5 weeks were injected intraperitoneally for 5 weeks with CSF1R (clone AFS98, BioXcell) blocking antibody or IgG isotype at 10 mg/kg in 100 μl phosphate -buffered saline (PBS). Antibodies were administrated twice per week. DIO mice (already HFD-feeding for 8 weeks) were administered intraperitoneally for 4 weeks with either small-molecule compound (AN-465/42243987) or vehicle at 0.5 mg/kg, twice per week.

All animal studies were supervised and approved by the Animal Care Committee of Shanghai Sixth People’s Hospital affiliated to Shanghai Jiao Tong University School of Medicine, and the animal welfare ethics acceptance number is No: 2018-0133. Mice were housed in laboratory cages at 22–24 °C, 50–60% humidity under controlled conditions (12 h light/dark cycle) with free access to food and water. The animals were housed with 3–5 mice per cage. Mice were maintained on a NCD (P1200F, Shanghai Puluteng Co. Ltd) or a HFD (60% kcal fat; D12492, Research Diets). For diet-induced obesity, eight-week-old mice were fed for 6 to 12 weeks (for male) and 14 weeks (for female). To safeguard animal welfare, mice were monitored on a daily basis and weighted every week. If there were changes in physical condition such as abnormal coat condition or posture, lameness, loss of body weight, and excessive licking or scratching of any mice, we will give more frequent observations. Humane endpoint was set includes excessive reduced locomotor activity (inability to access food and water), dehydration, and excess weight loss (>20% of body weight) within a few days. Since our DIO model is to treat animal with high fat diet which induces body weight increase, we did not observe any mice reached the humane endpoint. At the end of the experiment (for DIO mice: HFD for 6–14 weeks; for NCD mice: feeding for 22 weeks), mice were euthanized via CO_2_-dependent asphyxiation and tissues were harvested.

### Metabolic phenotyping

Whole-body fat and lean mass was measured by MRI (MAG-MED AccuFat-1050). For the glucose and insulin tolerance tests, the mice were intraperitoneally injected with 1.0–1.5 g per kg body weight (g/kg) glucose and 1.0–1.5 U/kg insulin after 6 h of fasting, respectively. Blood was collected from the tail and glucose was measured using a hand-held glucometer (Accu-Chek glucose reader; Roche) before injection (0 min) or at 15, 30, 60, 90, or 120 min after injection. To measure insulin levels in response to glucose, blood was collected from tail punctures at 0, 15, 30, 60, 90, or 120 min after injection, and insulin was measured using Mouse Adipokine Magnetic Bead Panel (MADKMAG-71K, MILLIPLEX). Serum leptin, IL-6, Ccl2, and Tnf-α concentrations were also detected with Mouse Adipokine Magnetic Bead Panel. Serum IL-1β was measured using an ELISA kit (MLB00C, R&D). Plasma triglyceride (TG, 290-63701) level was measured enzymatically using kits from WAKO chemicals. Total cholesterol (TCH, 10376501), ALT (03036926), and AST (03039631) were measured using kits from Siemens Healthcare Diagnostics Inc on an ADVIA 2400 Chemistry System. Plasma NEFA levels were determined using the enzymatic endpoint method (NEFA FS; 157819910930, DiaSys Diagnostic Systems). To analyze hepatic TGs, briefly, 40–50 mg of liver tissue was homogenized in PBS and mixed with CHCl_3_/CH_3_OH (2:1 [vol/vol]). The organic phase was transferred, air-dried overnight, and resuspended in 1% Triton X-100 in absolute ethanol, according to the manufacturers’ instructions^63^. The concentrations of TGs were similarly determined using the serum triglyceride determination kit (TG, 290-63701, WAKO). Total protein amounts of samples were assayed using a BCA protein assay kit (P00125, Beyotime Biotechnology) and Hepatic TGs were normalized by total protein content. To analyze energy expenditure, animals were placed in an OxyMax Comprehensive Laboratory Animal Monitoring System (CLAMs, Columbus Instruments) for analysis of oxygen consumption (VO_2_), physical activity, and food intake at 22 °C for 24 h. Airflow rate was 380 ml/min; O_2_ and CO_2_ concentrations in room air were measured every 15 min. VO_2_ was calculated after normalization to body weight.

### Cell culture and stimulation

To isolate primary mouse BMDMs, 6 to 11-week-old mice were euthanized. In brief, their femurs and tibias were collected in sterile conditions, and all the tissues were removed from the bones. Each end of bone was cut off and the bone marrow was flushed out using a 1 ml syringe filled with Dulbecco’s Modified Eagle’s Medium (DMEM, 11995, GIBCO). After centrifuging at 300 × *g* for 5 min, the pellets were collected and dissociated in lysis buffer to lyse red blood cells. A single cell suspension was prepared by passing the cells through a 40 μm nylon cell strainer (Falcon). Bone marrow cells were cultured and differentiated for 7 days in DMEM containing 10% FBS (10099, GIBCO) and 1% penicillin/streptomycin (P/S, 15140-122, GIBCO) supplemented with murine recombinant M-CSF (315-02-500, Peprotech) at 37 °C with 5% CO_2_. Differentiation was confirmed by F4/80 expression using flow cytometry. For macrophage M1 polarization, cells were treated with lipopolysaccharide (LPS, 200 ng/ml, tlrl-eblps, InvivoGen) for 24 h. To assess the TLR-induced NF-κB pathway, murine BMDMs were stimulated with LPS (200 ng/ml) or palmitic acid (PA, 250 μM, Sigma-Aldrich) for different time periods. DMSO or BSA was used as a vehicle control. Pro-inflammatory cytokines in the cell supernatants were assessed with Elisa kit, including Tnf-α (MTA00B, R&D), IL-6 (M6000B, R&D), and Ccl2 (MJE00B, R&D). The level of cAMP was determined using Elisa kit (K019-H1, ARBOR ASSAYS). For stimulation of IL-1β P17 and Casp-1 p20, ATP (tlrl-atp1, Invivogen) and Nigeicin (tlrl-nig, Invivogen) were used. For cell-signaling studies, BMDMs were pre-incubated with a PKA inhibitor (H-89; 50 µM, 130964-39-5, Cayman Chemical) for 30 min and subsequently exposed to LPS for the indicated time points.

For mouse primary pre-adipocytes and HEK293T cells (GNHu17, Cell Bank of Type Culture Collection of the Chinese Academy of Sciences, Shanghai, China) culture, cells were maintained in DMEM (11995, GIBCO) containing 10% FBS (10099, GIBCO) and 1% P/S (15140-122, GIBCO) and kept at 37 °C and 5% CO_2_. To induce pre-adipocyte differentiation, at 48 h post-confluence (day 0), the cells were cultured in differentiation medium containing 10% FBS, 0.5 mM 3-isobutyl-1-methylxanthine (IBMX, 17018, Sigma-Aldrich), 1 μM dexamethasone (D4902, Sigma-Aldrich) and 1 μg/ml insulin (16634, Sigma-Aldrich) until day 2. The cells were then cultured with DMEM supplemented with 10% FBS and 1 μg/ml insulin for 2 days, after which they were cultured with DMEM containing 10% FBS. The medium was changed every two days. For human monocyte THP-1 cells (TCHu57, Cell Bank of Type Culture Collection of the Chinese Academy of Sciences, Shanghai, China), RPMI 1640 (11875093, GIBCO) containing 10% FBS, 1% P/S, and supplemented with HEPES, was used. For transfection, HEK293T cells were transfected with indicated plasmids by Lipofectamine 2000 (116668, Invitrogen).

### Histologic analysis

Adipose tissues or livers were dissected, fixed in 4% PFA (P0099, Beyotime Biotechnology) for at least 24 h, and embedded in paraffin. Paraffin tissue sections were stained with hematoxylin and eosin (H&E) or analyzed by immunohistochemistry (IHC). Frozen sections were prepared for oil red staining or immunofluorescence. For IHC, after the paraffin was removed and antigen was unmasked, sections were incubated with primary antibodies against F4/80 (14-4801-82, eBioscience, 1:50) and GPSM1 (11483-1-AP, Proteintech, 1:50), followed by incubation with the secondary antibodies conjugated with horseradish peroxidase. The sections were then treated with the DAB Staining Kit (PV6000, ZSGB-BIO) according to the manufacturer’s instruction. For immunofluorescence analysis, the following antibodies were used: F4/80 (eBioscience, 1:50), GPSM1 (Proteintech, 1:50), DAPI (C1002, Beyotime Biotechnology, 1:5000), p-P65 (3033, CST, 1:50).The secondary antibodies were Alexa Fluor 488 donkey anti-mouse IgG (R37114, 1:1000), Alexa Fluor 647 donkey anti-rabbit IgG (A-31573, 1:1000), and Alexa Fluor 546 donkey anti-rabbit IgG (A10040, 1:1000) purchased from Thermo Fisher Scientific. Images were then captured using a fluorescence microscope (Leica) and analyzed by ImageJ (National Institutes of Health) version 1.52.

### Isolation of stromal vascular fractions and macrophages from WAT

Mouse eWAT or scWAT were isolated and cleaned with PBS (D8537, Sigma-Aldrich) to remove impurities and blood. The tissues were cut and minced into small pieces approximately 1.0 mm^3^ and subsequently digested with digestion buffer containing PBS, 1% HEPES (H0887, Sigma-Aldrich), and 0.2% collagenase type II (C6885, Sigma-Aldrich) at 37 °C with shaking at 150 rpm for 20 min. An equal volume of complete culture medium was added and the solution mixed to halt the digestion. The digested solution was filtered through a 100 μM nylon (Falcon) mesh and centrifuged at 1200 × *g* for 10 min. The SVF pellets were collected and resuspended in lysing buffer (555899, BD Pharmingen) to lyse red blood cells before further analysis. Anti-F4/80 MicroBeads (130-110-443, MiltenyiBiotec) was used for sorting F4/80^+^ macrophages from SVFs.

### Isolation of peritoneal macrophages

For analysis of GPSM1 ablation in macrophages, peritoneal macrophages were isolated from mice at 4 d after intraperitoneal injection of thioglycollate broth (70157, Sigma-Aldrich). Peritoneal cells were obtained by washing the peritoneal cavity with ice-cold PBS and erythrocytes were removed by incubating with red blood cell lysis buffer. Macrophages were purified by adherence to appropriate culture plates using DMEM containing 20% FBS and 1% P/S. Non-adherent cells were removed by washing twice with DMEM. Peritoneal macrophages were cultured for 7 days in DMEM containing 10% FBS and 1% P/S for further biochemical analysis.

### White blood cell counts

Leukocytes and differential blood cell counts were quantified from whole blood using a hematology cell counter (ProCyte Dx, IDEXX).

### Flow cytometry analysis

For SVFs isolated from mice scWAT and eWAT, cells were resuspended in Stain Buffer (554656, BD Pharmingen) and stained with indicated fluorescent-conjugated antibodies for 30 min at 4 °C in the dark. The antibodies used for macrophage analysis included PE-CY7-anti-CD45 (552848, BD Pharmingen, 0.06 μg/test), FITC-anti-F4/80 (11-4801-82, eBioscience, 0.5 μg/test), APC-anti-F4/80 (17-4801-82, eBioscience, 1 μg/test), APC-anti-CD11b (553312, BD Pharmingen, 0.6 μg/test), APC-anti-CD11c (550261, BD Pharmingen, 0.6 μg/test), AF647-CD206 (565250, BD Pharmingen, 0.6 μg/test), and AF488-TIM-4 (53-5866-82, eBioscience, 1 μg/test). The antibodies used for neutrophil sorting included FITC-anti-Gr-1 (11-5931-82, eBioscience, 0.25 μg/test) and APC-anti-CD11b (553312, BD Pharmingen, 0.6 μg/test). For bone marrow cells collected from leg bones, cells were lysed to remove RBCs and filtered before use. Fc receptors were blocked using anti-FcγRII/III antibody 2.4G2 (553141, BD Pharmingen, 1:140) prior to adding the fluorescent-tagged antibodies. Myeloid progenitor cells (MPCs) were identified as lineage^−^Sca1^−^cKit^+^. The following antibodies were used: FITC-anti-mouse hematopoietic lineage (22-7770-72, eBioscience, 20 μl/test), APC-anti-SCA1 (17-5981-82, eBioscience, 0.2 μg/test), and eFluor450-anti-CD117 (48-1171-82, eBioscience, 0.25 μg/test). For peripheral blood monocyte analysis, 100 μl of blood was collected into EDTA tubes before RBC lysis, filtration, and staining, for 30 min on ice. Fc receptors were blocked using anti-FcγRII/III antibody 2.4G2. Monocytes were identified as CD11b^+^CD115^+^ and further identified into Ly6C^hi^ and Ly6C^low^ subsets. The antibodies used were as follows: APC-anti-CD115 (17-1152-82, eBioscience, 0.2 μg/test), FITC-anti-CD11b (557396, BD Pharmingen, 1.5 μg/test), and PE-CY7-anti-LY6C (25-5932-82, eBioscience, 0.125 μg/test). Dead cells were excluded from all samples using DAPI, fixable viability stain 780 (565388, BD Pharmingen, 1:1000), and fixable viability stain 510 (564406, BD Pharmingen, 1:1000). The cells marked with the antibodies were then washed three times with Stain Buffer. Samples were then subjected to flow cytometry analysis with ImageSteam Mark II (Luminex). Data were analyzed with IDEAS software (Version 6.2).

### In vivo matrigel chemotaxis assay

Each mouse received two Matrigel plugs three days prior to euthanasia as described^64,65^. Briefly, growth factor-reduced Matrigels (354230, BioCoat, R&D Systems) were subcutaneously injected into the right and left flank of each mouse, one plug containing MCP-1 (500 ng/ml) and the other plug containing vehicle. After euthanasia, plugs were surgically removed, cleaned, and digested with dispase (Invitrogen). Cells were stained with Calcein/AM (Invitrogen) and counted using an automated fluorescent cell counter (Countess II FL, ThermoFisher).

### Generation of *GPSM1* KO human monocyte-like cell lines (THP-1)

To stably delete the expression of *GPSM1* in THP-1 cells, CRISPR-Cas9 gene-editing technology mediated by electroporation (Neon, MPK5000) was used (Cyagen, China). Briefly, vectors loaded with Cas9 gene and sgRNA targeting *GPSM1* were transfected into THP-1 cells, before single cell clones were isolated by serial dilutions. Sequence for sgRNA used was as follows: CTGCCTAGAGCTGGCGCTGG. The loss of *GPSM1* in THP-1 cells was verified by PCR and DNA sequencing. The PCR primers used were as follows: Forward (5′CTGGGAGCCTCAGTTGTTCTT3′) and Reverse (5′CCTCCTCAAACTGTAACTGCTGA3′).

### Monocyte Chemotaxis Assay

THP-1 monocytes (10^6^ cells/ml) were stained with Calcein/AM (Invitrogen) for 30 min and then loaded into the upper wells of a 24-well transwell plate (CLS3421, Corning). The lower wells contained either vehicle or MCP-1 (R&D Systems, 150 ng/ml). The cells were incubated for 3 h at 37 °C and 5% CO_2_. Transmigrated cells were observed and counted in five separate fields at 10 × magnification under a fluorescence microscope.

### Virus-mediated RNAi knockdown or overexpression

Mouse BMDMs (1 × 10^6^ to 2 × 10^6^ cells) were prepared and infected at a multiplicity of infection (MOI) of 30–50 with lentivirus-shRNA, Adenovirus-shRNA, and Adenovirus-overexpression. Lentivirus-shRNAs (sh*GPSM1* and sh*A20*), Adenovirus-shRNAs, (sh*Gα*_*i3*_) and Adenovirus-GPSM1were prepared by Shanghai GeneChem. BMDMs were infected with lentivirus or adenovirus for 72 h or 48 h, separately, before further treatments.

### RNA sequencing (RNA-seq) and bioinformatics analysis

Total cellular RNAs of BMDMs (Seq1: *GPSM1*^f/f^ versus *GPSM1*^f/f^; *Lyz2*-Cre BMDMs after LPS stimulation for 1 h; Seq2: BMDMs infected with Lv-shNC versus Lv-shGPSM1 after LPS stimulation for 3 h) were extracted using TRIzol reagent (15596018, Invitrogen). Quantity and quality of each mRNA sample were examined using a Nanodrop (ThermoFisher) and through electrophoresis, respectively. RNA libraries were constructed using VAHTS® Universal V6 RNA-seq Library Prep Kit for IlluminaVazyme (Cat.N401-02), according to the manufacturer’s protocol, and paired-end reads were obtained on the Novaseq 6000 platform. Quality of RNA-seq data was estimated using RSeQC (version 2.6.4). Differentially expressed genes were identified using the RankProd49 package in R based on *P* value < 0.01.

### ChIP assay

Chromatin immunoprecipitation (ChIP) assays were performed using a SimpleChIP Enzymatic Chromatin IP Kit (9003, Cell Signaling Technologies). BMDMs from WT mice treated with 200 ng/ml LPS for 1 h were cross linked with 37% formaldehyde at a final concentration of 1% at room temperature for 10 min and glycine solution was added to stop the cross-linking reaction. Fragmented chromatin was treated with nuclease and subjected to sonication. Chromatin immunoprecipitation was performed overnight at 4 °C with rabbit anti- Phospho-CREB (Ser133) antibody (1:50, 9198, Cell Signaling Technologies) and normal rabbit IgG (1:250, 2729, Cell Signaling Technologies) as a negative control. Protein G Magnetic Beads were added for another 2 h at 4 °C. The chromatins were then washed and eluted from the protein G magnetic beads using buffers supplied with the kit and DNA was analyzed by quantitative PCR. The primers are listed in Supplemental Table 3 (Table S3).

### Luciferase reporter assays

JASPAR (http://jaspardev.genereg.net/) was used to analyze the potential transcription factor binding sites (TFBS) in the promoter of *Tnfaip3*(A20). The wild type A20 promoters were amplified from mouse genomic DNA and cloned into the pGL4.17-basic luciferase reporter vector (E6721, Promega). The mutant A20-luciferase reporter with a deletion at the putative CREB1 binding site (TGACGTGAC) was generated using ClonExpress Ultra One Step Cloning Kit (C115-01, Vazyme) (Supplementary Table 3). The CREB expression plasmid (pcDNA3.1-flag-CREB1) was purchased from Public Protein/Plasmid Library (BC010636). For luciferase reporter assays, 100 ng wild type or mutant A20 reporter vector, 200 ng CREB1 expression plasmid, and 10 ng pRL-TK (luciferase control reporter vector) were co-transfected into HEK-293T cells in duplicate 24-well plates using Lipofectamine 2000 (116668, Invitrogen), according to the manufacturer’s instructions. Cells were harvested to analyze the relative luciferase activity using dual-luciferase reporter assay system (Promega).

### Immunoblot analysis and immunoprecipitation

Lysates of cells or tissues were prepared with RIPA buffer (Beyotime Biotechnology) containing complete Protease Inhibitor Cocktail (Roche), and protein concentration was determined with the BCA Protein Assay Kit. Equal amounts of total protein separated by SDS–PAGE gels were transferred to nitrocellulose filter(NC) membrane (Millipore) and blocked. After incubation with the desired antibodies, blots were washed in TBST (Tris-buffered saline containing 0.1% Tween 20) three times and developed with Immunobilon Western Chemiluminescent HRP substrate (WBKLS0500, Millipore).The images were captured by ChemiDoc Imaging System (BioRad). Image Lab software version 6.0 for acquisition of images from Western blot. ImageJ (National Institutes of Health) version 1.52 for Western blot images densitometry analysis.

For immunoprecipitation, cells were treated as indicated and lysed in IP buffer (P0013, Beyotime Biotechnology) containing protease inhibitors (4693116001, Roche) followed by centrifugation at 12,000 × *g* for 10 min at 4 °C. Part of the supernatant was mixed with 5× loading buffer as the input sample. The remaining cell lysates were incubated with the Agarose Conjugated Anti-DYKDDDDK Affinity Beads (Smart-Lifesciences) at 4 °C overnight. On the following day, after three washes in IP buffer, beads were boiled with 2× loading buffer for 10 min and then subjected to Western blot analysis using the indicated antibodies. The following antibodies were used: β-Actin (1:2500, 4970), HSP90 (1:2500, 4877), Lamin A/C (1:1000, 4777), Phospho-NF-κB p65 (1:1000, 3033), NF-κB p65 (1:2000, 8242), Phospho-IκBα (1:1000, 9246), IκBα (1:1000, 9242), Phospho-IKKα/β (1:1000, 2697), IKKβ (1:1000, 2678), Phospho-AKT (1:1000, 4058), AKT (1:1000, 9272), IL-1β (1:1000, 12242), Cleaved-IL-1β (1:1000, 63124), Caspase-1 (1:1000, 24232), A20/TNFAIP3 (1:1000, 5630), Phospho-(Ser/Thr) PKA Substrate (1:1000, 9621), Phospho-CREB (1:1000, 9198), and CREB(1:1000, 9197) were from Cell Signaling. GAPDH (1:2500, sc-32233) was obtained from Santa Cruz Biotechnology. UCP1 (1:1000, ab10983) was from Abcam. GPSM1 (1:1000, 11483-1-AP) was from Proteintech. Cleaved-Caspase-1 (1:500, AG-20B-0042) was from AdipoGen. The capillary electrophoresis was performed using Jess System (ProteinSimple).

### Quantitative RT–PCR

Total RNA was extracted from tissues using QIAzol reagent (RNeasy Plus Universal Mini Kit, 1023537, QIAGEN). Total RNA was extracted from cells using TRIzol reagent (15596018, Invitrogen). cDNA was generated from 1 μg RNA using RT reagent Kit (RR047A, TaKaRa), and real-time PCR analyses were performed with SYBR Green Master Mix (01000432, Applied Biosystems) using a QuantStudio Real-Time PCR System (Applied Biosystems) according to the manufacturer’s instructions. Primers are listed in Supplementary Table 4 (Table S4).

### Allosteric pocket prediction and structure-based virtual screening

We first downloaded the predicted structure of GPSM1 from the AlphaFold Protein Structure Database (https://alphafold.ebi.ac.uk/entry/Q86YR5). We deleted the regions with low confidence score (Residue indexes 26–386 were preserved). Next, based on the predicted structure, one potential allosteric pocket (mainly consist of residues Gly336, Asn337, Val340, Gly343, Arg344, Pro345, Ala348, Ala352, Asn374, Gln377, Leu378, and Val381) was detected with the help of the AlloSite v2.0 web server (http://mdl.shsmu.edu.cn/AST/). Thereafter, the allosteric pocket of GPSM1 was used as a target for HTVS of the small molecules of interest from the SPECS library (https://www.specs.net/index.php) via docking methods^66^. Firstly, we refined the protein structure using default parameters of the Protein Preparation Wizard module of Schrodinger Software Suite (Schrödinger, LLC, New York, NY). Secondly, we defined the grid center of the pocket as coordinates (−34.85, −4.07, 11.04). Thirdly, the SPECS database was submitted to the Glide program encoded in Schrodinger Software and 50,000 compounds with the highest score were first selected after standard precision (SP) docking screening with default parameters to run the second round screening in extra precision (XP). The 1000 molecules with the top-ranked XP scores were further selected manually to evaluate molecular diversity, shape complementarity, interaction mode, and their capacity to form hydrogen bonds in the binding pocket. Finally, 93 potential compounds were purchased from the Specs Company for further experimental verification.

### Affinity measurement

The Surface Plasmon Resonance (SPR) method uses a BIAcore 8K (GE Healthcare) to measure binding affinities. GPSM1 Recombinant Protein was diluted in a sodium acetate solution (pH 4.0) with a final concentration of 20 μg/mL. Recombinant GPSM1 protein (H00026086-P02, abnova) was immobilized on a CM5 sensor chip (GE Healthcare) by amine coupling to reach target densities of 10,000 resonance units (RU). Immobilized GPSM1 was used to capture the chemical compound. The running buffer contained PBS-T and 5% DMSO. The small-molecular compounds (3.125, 6.25, 12.5, 25 µM, and 50 µM) were injected at a rate of 30 μL/min for 120 s in single-cycle mode. In the dissociation phase, the running buffer was injected at a rate of 30 μL/min for 120 s. Data were recorded at 25 °C. The binding profiles are shown with time (s) on the x-axis and response units (RU) on the y-axis. The data were analyzed and processed using BIAevaluation Software (BiaCore).

### High-content screening

BMDMs were incubated with 10 small-molecule compounds at indicated concentrations for 16 h and subsequently exposed to LPS for 30 min in CellCarrier 96-well black-walled microplates (PerkinElmer). Formalin-fixed triton-permeabilized cells were stained with P65 antibody (1:75, 8242, Cell Signaling Technologies) overnight at 4 °C followed by incubation with the secondary antibody Alexa Fluor 647-labeled Goat Anti-Rabbit IgG (1:500, A0468, Beyotime). Nuclei were counter-stained using DAPI dihydrochloride (1:2000, C1002, Beyotime). Images were then captured using an Operetta® CLS™ high-content analysis system with a 20× water objective NA 1.0 under non-confocal mode. Cell morphology was analyzed and nuclei and cytoplasm intensities were calculated based on the Harmony software (Perkin Elmer) version 4.9.

### Quantification and statistical analysis

All data in this study are presented as means with standard deviations (SDs) or standard errors (SEs), median (interquartile range), *n* (%), and odds ratios (ORs) with 95% confidence intervals (CIs). Quantitative traits with a skewed distribution were logarithmically transformed to approximate univariate normality. All experiments were replicated at least 3 times. Statistical analysis was performed with unpaired two-tailed Student’s *t* test, or one-way analysis of variance (ANOVA) followed by Tukey’s post-hoc test, or two-way analysis of variance (ANOVA) with Sidak’s multiple-comparisons test, in GraphPad Prism (version 8.3.0; GraphPad Software Inc.) or SAS software (version 8.0; SAS Institute, Cary, NC, USA).

### Reporting summary

Further information on research design is available in the Nature Portfolio Reporting Summary linked to this article.

## Supplementary information

Supplementary information

Reporting Summary

## Data Availability

All data supporting the findings of this study are available within the main manuscript and the supplementary files. RNA-Seq data can be viewed in National Omics Data Encyclopedia (NODE) under the accession code OEP003692 or through the URL. Source data are provided with this paper. A reporting summary for this article is available as a Supplementary File. Source data are provided with this paper.

## Source data

Source Data
