# Supplementary material for: GPSM1 impairs metabolic homeostasis by controlling a pro-inflammatory pathway in macrophages
Source: Nat Commun. 2022 Nov 25;13:7260. doi: 10.1038/s41467-022-34998-9 (PMC9700814; doi:10.1038/s41467-022-34998-9)

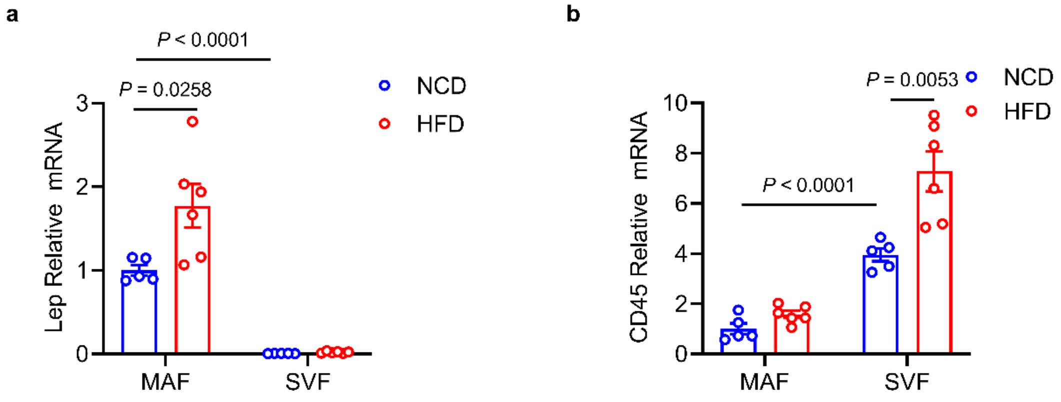

Supplementary Fig.1 Fractionation studies of eWAT.

a, RT-qPCR of *Leptin* expression in SVF and MAF isolated from eWAT from NCD (n= 5 biologically independent sample) and HFD (n= 6 biologically independent sample) mice. **b**, RT-qPCR of *CD45* expression in SVF and MAF isolated from eWAT from NCD (n= 5 biologically independent sample) and HFD (n= 6 biologically independent sample) mice. Throughout, data are presented as means ± SEM. *P* values are determined by unpaired two-tailed Student's t-test.

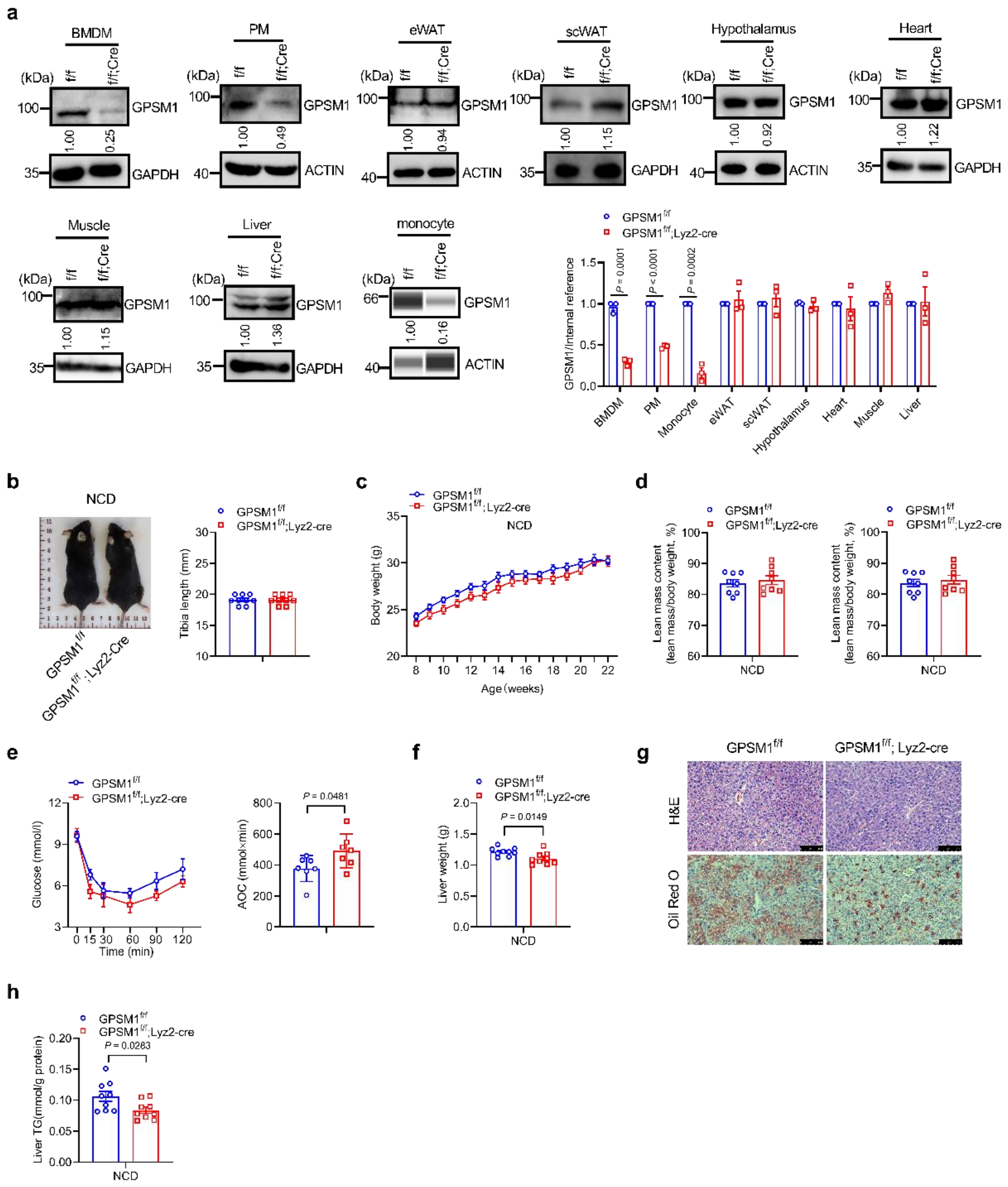

Supplementary Fig.2 Metabolic characterization of male mice fed a NCD.

a, Representative immunoblot analysis and quantification of GPSM1 expression in BMDMs, Peritoneal macrophages (PMs), monocytes and the indicated tissues from *GPSM1*$^{f/f}$; *Lyz2*-cre mice and *GPSM1*$^{f/f}$ mice (n = 3 biologically independent mice per group). GAPDH and actin were used as loading controls. **b-g**, Male *GPSM1*$^{f/f}$; *Lyz2*-cre mice and age-matched *GPSM1*$^{f/f}$ littermates were fed a NCD for 22 weeks. **b**, Representative image of mice (left) and quantification of tibia length (right) of the indicated genotype (n = 9 biologically independent mice per group). **c**, Body weight (n = 10 biologically independent mice per

group). **d**, Percent of fat (left), lean body mass (right). n = 8 biologically independent mice per group. **e**, ITT and AOC (n = 7 biologically independent mice per group). **f**, Liver weight (n = 9 biologically independent mice per group). **g**, Representative images of H&E staining (top) and Oil Red O (bottom) staining of liver sections. Scale bars, 100 μm. **h**, Quantification of hepatic TG (right, n = 9 biologically independent mice per group). Independent experiments were repeated three times with similar results (**g**). All data are shown as means ± SEM (**a-f**, **h**). *P* values are determined by unpaired two-tailed Student's *t*-test (**a**, **b**, **d**, **f** and **h**) or two-way ANOVA with Sidak's multiple-comparisons test (**c**, **e**).

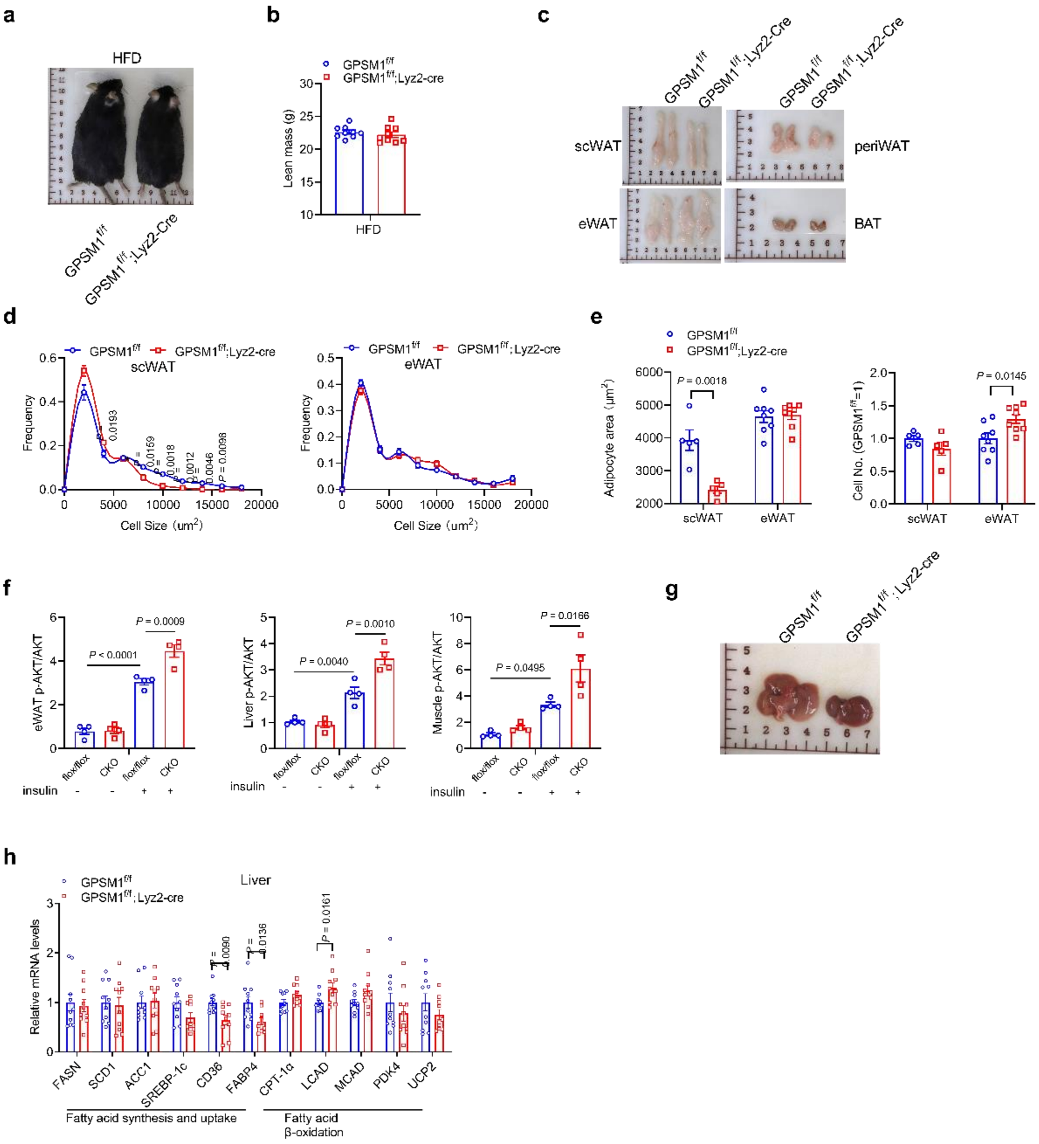

Supplementary Fig.3 Metabolic characterization of male mice fed a HFD.

a, Representative images of 7-week-old mice subjected to a HFD for 12 weeks. **b**, Lean mass was determined for mice fed a HFD (n = 9 biologically independent mice per group). **c**, Representative images of the indicated WAT from *GPSM1*$^{f/f}$; *Lyz2*-cre and *GPSM1*$^{f/f}$ mice. **d**, Frequency distribution of adipocyte size of scWAT and eWAT (n = 5 biologically independent mice per group for scWAT and n = 8 biologically independent mice per group for eWAT). **e**, Quantification of adipocyte area (left) and cell number (right) of scWAT and eWAT. **f**, Quantification of pAKT/AKT in eWAT, liver and muscle (n = 4 biologically independent mice per group). **g**, Representative images of liver section. **h**, RT- qPCR analysis showing mRNA abundance of fatty acid synthesis and uptake genes and fatty acid

β-oxidation genes in liver (n = 10 biologically independent mice per group). All data are shown as means ± SEM. *P* values are determined by unpaired two-tailed Student's *t*-test (**b**, **d**, **e**, and **h**) or one-way ANOVA with Tukey's correction for multiple group comparison (**f**).

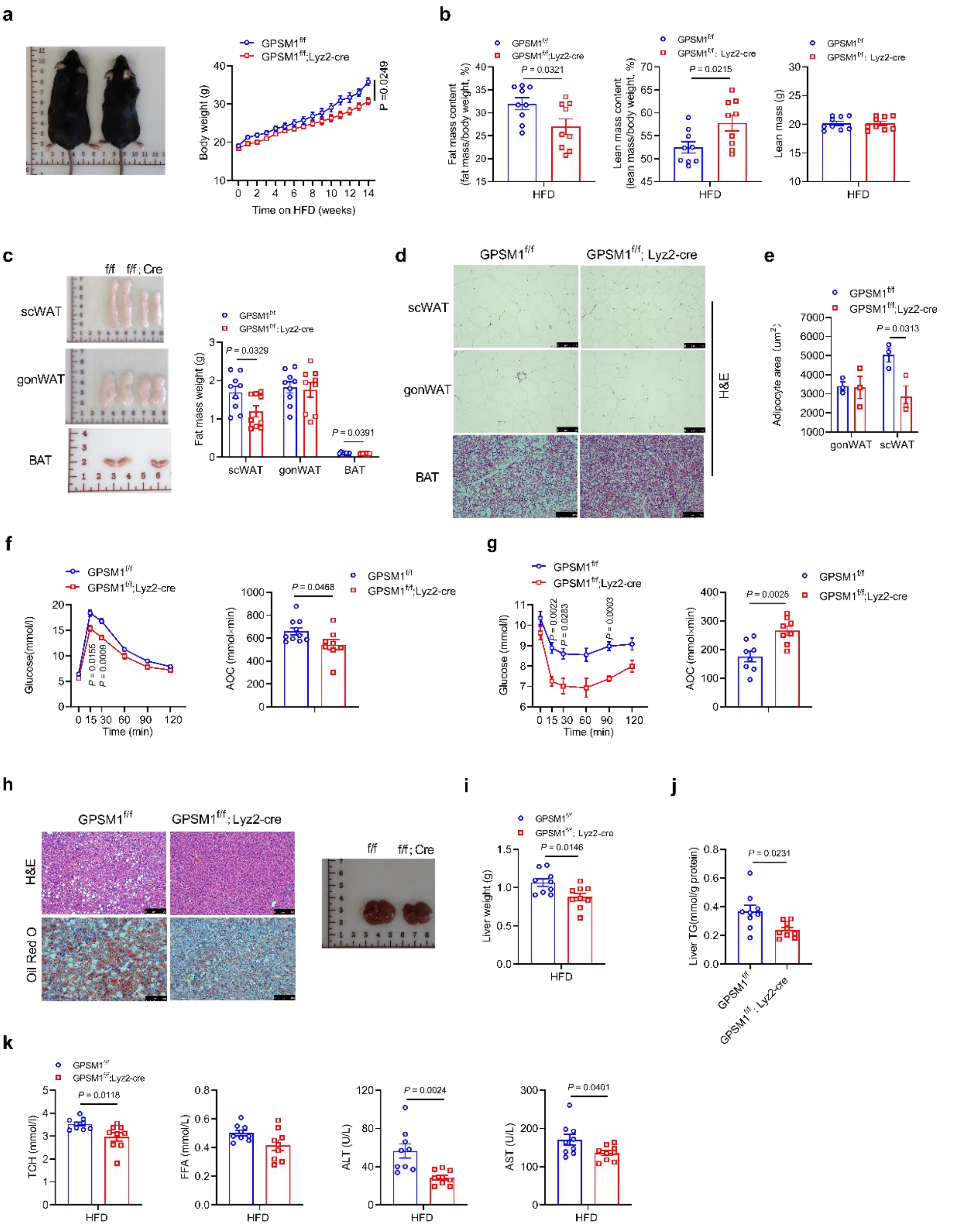

Supplementary Fig.4 GPSM1 deficiency alleviates HFD-induced metabolic disorders in female mice.

Female *GPSM1*$^{f/f}$; *Lyz2*-cre mice and age-matched *GPSM1*$^{f/f}$ littermates were fed a HFD for 14 weeks. HFD feeding started at 7 weeks of age. **a**, Representative images of mice (left) and body weight (right, n = 10 biologically independent mice per group). **b**, Percent of fat (left), lean body mass (middle), and lean mass weight (right). n = 9 biologically

independent mice per group. **c**, Representative images (left) and weight (right) of the indicated WAT from *GPSM1*$^{f/f}$; *Lyz2*-cre and *GPSM1*$^{f/f}$ mice. n = 9 biologically independent mice per group. **d**, Representative H&E staining of scWAT, gonWAT and BAT sections. Scale bars, 100 μm. **e**, Adipocyte area of scWAT and gonWAT (n = 3 biologically independent mice per group). **f**, GTT and AOC (n = 10 biologically independent mice for *GPSM1*$^{f/f}$ and n = 8 biologically independent mice for *GPSM1*$^{f/f}$; *Lyz2*-cre). **g**, ITT and AOC (n = 8 biologically independent mice per group). **h**, Representative images of H&E staining (top) and Oil Red O (bottom) staining of liver sections (left). Scale bars, 100 μm. Representative images of liver section (right). **i**, Liver weight (n = 9 biologically independent mice per group). **j**, Quantification of hepatic TG (n = 9 biologically independent mice per group). **k**, Serum levels of TCH, NEFA, ALT, and AST (n = 9 biologically independent mice per group). Independent experiments were repeated three times with similar results (**d**, **h**). All data are shown as means ± SEM. *P* values are determined by unpaired two-tailed Student's *t*-test (**b**, **c**, **e** to **g**, **i** to **k**) or two-way ANOVA with Sidak's multiple-comparisons test (**a**, **f**, and **g**).

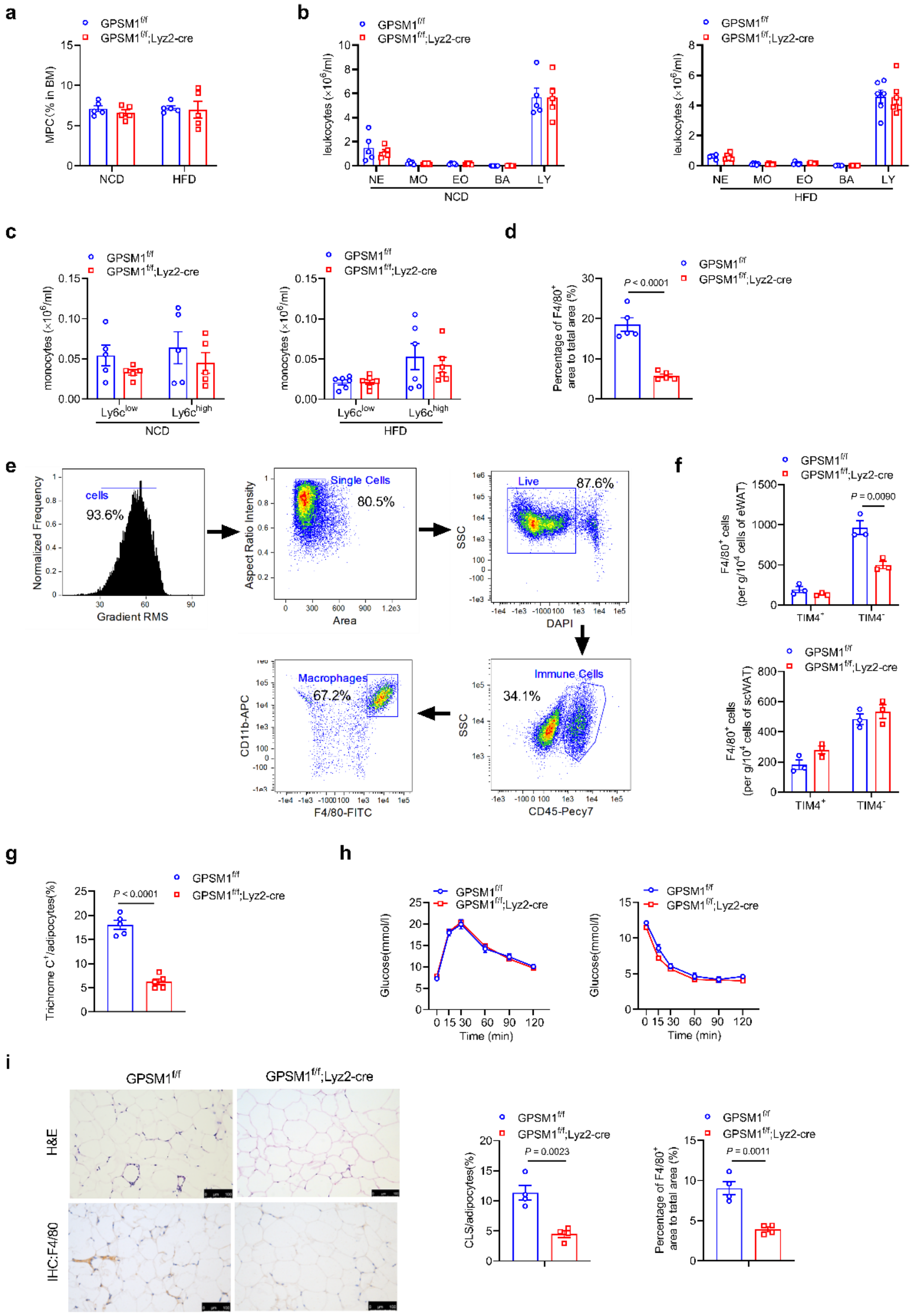

Supplementary Fig.5 Analysis of metabolic inflammation in HFD-fed mice.

a, Flow cytometry quantification of the Lin$^-$Sca1$^-$cKit$^+$ myeloid precursors (MPCs) in the bone marrow of *GPSM1*$^{f/f}$ and *GPSM1*$^{f/f}$; *Lyz2*-cre mice both under NCD (12-week old) and HFD for 8 weeks (n = 5 biologically independent mice per condition). **b**, White blood cell counts both under the NCD (n = 5 biologically independent mice per group) and HFD

settings (n = 6 biologically independent mice per group). NE, neutrophils; MO, monocytes; EO, eosinophils; BA, basophils; LY, lymphocytes. **c**, The numbers of Ly-6C^{low} and Ly-6C^{high} monocytes of *GPSM1*$^{f/f}$ and *GPSM1*$^{f/f}$; *Lyz2*-cre mice both under the NCD (n = 5 biologically independent mice per group) and HFD (n = 6 biologically independent mice per group) conditions. **d** to **g** Male *GPSM1*$^{f/f}$; *Lyz2*-cre mice and age-matched *GPSM1*$^{f/f}$ littermates were fed a HFD for 12 weeks. HFD feeding started at 7 weeks of age. **d**, Quantification of the proportion of crown-like structure (n = 5 biologically independent mice per group). **e**, Gating strategy for analysis of macrophages in eWAT and scWAT. **f**, The numbers of TIM4^{+} and TIM4^{-} macrophages of eWAT and scWAT (n = 3 biologically independent mice per group). **g**, Quantification of the proportion of Trichrome C^{+} area (n = 5 biologically independent mice per group). **h** and **i** Male *GPSM1*$^{f/f}$; *Lyz2*-cre mice and age-matched *GPSM1*$^{f/f}$ littermates were fed a HFD for 5 weeks. HFD feeding started at 7 weeks of age. **h**, GTT and ITT (n = 9 biologically independent mice for *GPSM1*$^{f/f}$ and n = 10 biologically independent mice for *GPSM1*$^{f/f}$; *Lyz2*-cre). **i**, Representative H&E and F4/80^{+} staining of eWAT sections and quantification (n = 4 biologically independent mice per group). Scale bars, 100 μm. All data are presented as means ± SEM. *P* values are determined by unpaired two-tailed Student's *t*-test (**a** to **d, f**, **g**, **i**) or two-way ANOVA with Sidak's multiple-comparisons test (**h**).

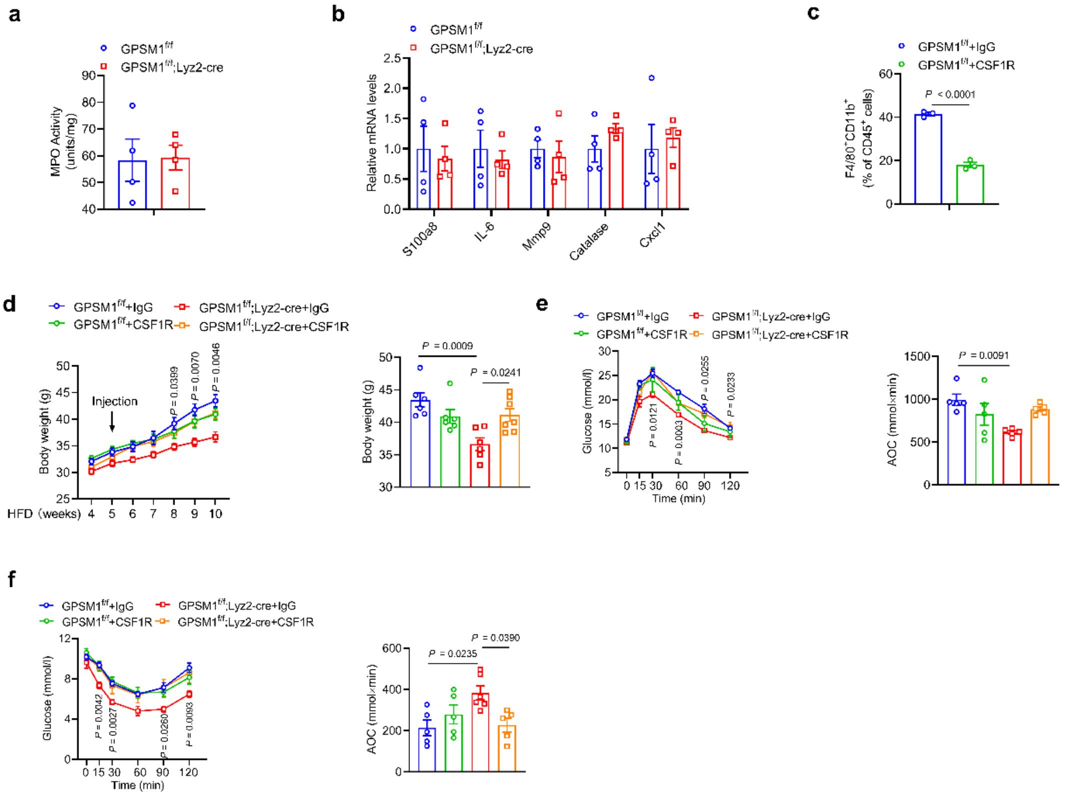

Supplementary Fig.6 GPSM1 depletion does not affect inflammatory properties of neutrophils and characterization of CSF1R-adminstered mice fed a HFD.

a and **b** Male *GPSM1*$^{f/f}$; *Lyz2*-cre mice and age-matched *GPSM1*$^{f/f}$ littermates were fed a HFD for 12 weeks. **a**, The myeloperoxidase (MPO) activity in eWAT (n = 4 biologically independent sample per group). **b**, RT-qPCR analysis indicating mRNA abundance of neutrophil inflammatory markers from sorted CD11b^{+}Ly6G^{+} neutrophils from the eWAT by flow cytometry (n = 4 biologically independent sample per group). **c** to **f** *GPSM1*$^{f/f}$ and *GPSM1*$^{f/f}$; *Lyz2*-cre mice, which had been already HFD-fed for 5 weeks, injected intraperitoneally CSF1R antibody or isotype IgG (10 mg/kg), twice a week, for 5 weeks. **c**, Flow cytometry quantification of total macrophages of eWAT (n = 3 biologically independent mice per group). **d**, Body weight curve (left) and the body weight at 10-week HFD (right). n = 6 biologically independent mice for *GPSM1*$^{f/f}$ injected with IgG or CSF1R and *GPSM1*$^{f/f}$; *Lyz2*-cre injected with IgG; n = 7 biologically independent mice for *GPSM1*$^{f/f}$; *Lyz2*-cre injected with CSF1R. **e**, GTT and AOC (n = 5 biologically independent mice per group). **f**, ITT and AOC (n = 5 biologically independent mice for *GPSM1*$^{f/f}$ injected with IgG or CSF1R and *GPSM1*$^{f/f}$; *Lyz2*-cre injected with CSF1R; n = 6 biologically independent mice for

GPSM1$^{f/f}$; *Lyz2*-cre injected with IgG). All data are shown as means ± SEM. *P* values are determined by unpaired two-tailed Student's *t*-test (**a** to **c**), two-way ANOVA with Tukey's multiple-comparisons test (**d** to **f**) or one-way ANOVA with Tukey's multiple-comparisons test (**d** to **f**).

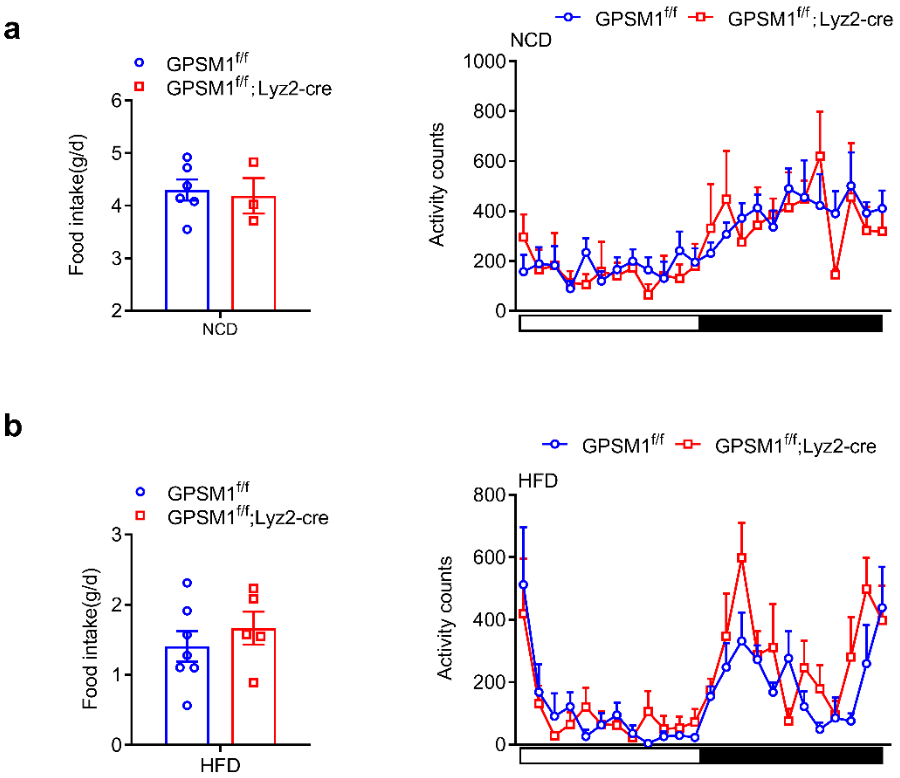

Supplementary Fig.7 Metabolic cage studies of NCD- and HFD-fed mice.

a Food intake and Activity counts of NCD-fed mice (n = 6 biologically independent mice for *GPSM1*$^{f/f}$ and n = 3 biologically independent mice for *GPSM1*$^{f/f}$; *Lyz2*-cre) were monitored for a 24 h period. **b** Food intake and Activity counts of HFD-fed mice (n = 7 biologically independent mice for *GPSM1*$^{f/f}$ and n = 5 biologically independent mice for *GPSM1*$^{f/f}$; *Lyz2-*cre) were monitored for a 24 h period. Throughout, data are presented as means ± SEM. *P* values are determined by unpaired two-tailed Student's *t*-test and two-way ANOVA with Sidak's multiple-comparisons test.

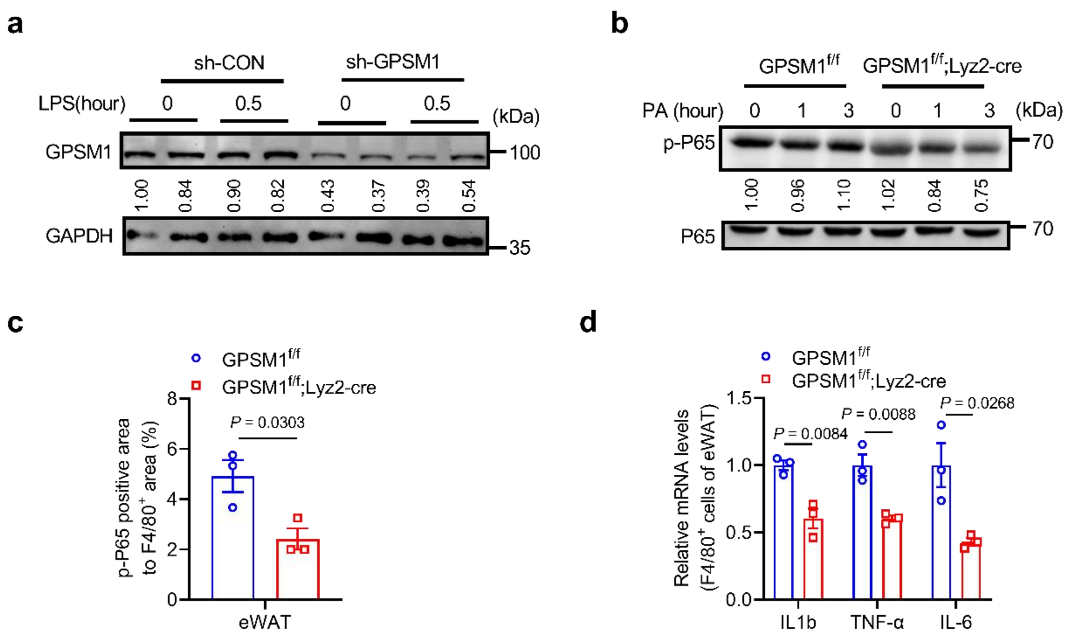

Supplementary Fig.8 Macrophage GPSM1 regulates TLR4-induced NF-κB inflammatory signaling.

a, BMDMs were infected with Lv-shCON or Lv-shGPSM1 for 72 h and treated with LPS for additional indicated times. Immunoblot analysis of GPSM1 is shown (n= 2 independent samples per condition). **b**, BMDMs were treated with 250 μM Palmic acid or vehicle control for indicated times. Immunoblot analysis of p-P65 and P65 is shown. **c**, Quantification of p-P65 positive area to F4/80^{+} area of eWAT from *GPSM1*$^{f/f}$ and *GPSM1*$^{f/f}$; *Lyz2*-cre mice subjected to HFD for 12 weeks (n = 3 biologically independent mice per group). **d**, RT-qPCR analysis of indicated genes from sorted F4/80^{+} macrophages of eWAT (n = 3 biologically independent mice per group). Independent experiments were repeated three times with similar results (**a**, **b**). Throughout, data are presented as means ± SEM. *P* values are determined by unpaired two-tailed Student's *t*-test (**c**, **d**).

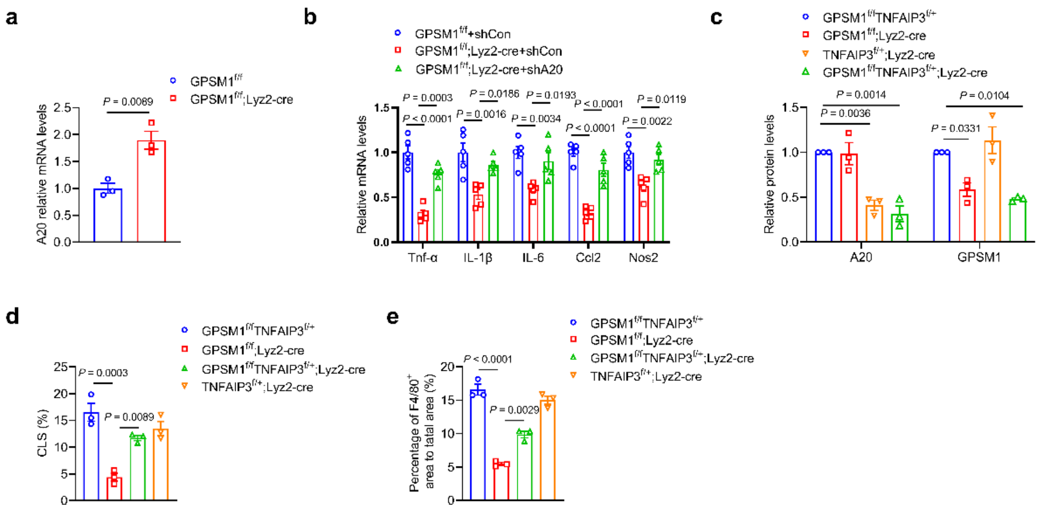

Supplementary Fig.9 TNFAIP3 functions as a GPSM1 target for mediating the NF-κB pathway in macrophages.

a, A20 relative mRNA levels of eWAT from HFD-fed *GPSM1*$^{f/f}$ and *GPSM1*$^{f/f}$;*Lyz2*-cre mice (n = 3 biologically independent sample per group, each sample was obtained from a pool of three mice). **b**, RT-PCR indicating pro-inflammatory markers in *GPSM1*$^{f/f}$ BMDMs infected with Lv-shCON and *GPSM1*$^{f/f}$;*Lyz2*-cre BMDMs infected with Lv-shCON or Lv-shA20 for 72 h and treated with LPS for 3 h (n= 5 independent samples per condition). **c**, Quantification of A20 and GPSM1 protein levels in BMDMs from the mice of four genotypes (n = 3 biologically independent mice per group). **d**, Quantification of the proportion of crown-like structure (n = 3 biologically independent mice per group). **e**, Quantification of the proportion of F4/80^{+} area (n = 3 biologically independent mice per group). All data are presented as means ± SEM. *P* values are determined by unpaired two-tailed Student's *t*-test (**a**) or one-way ANOVA with Tukey's multiple-comparisons test (**b** to **e**).

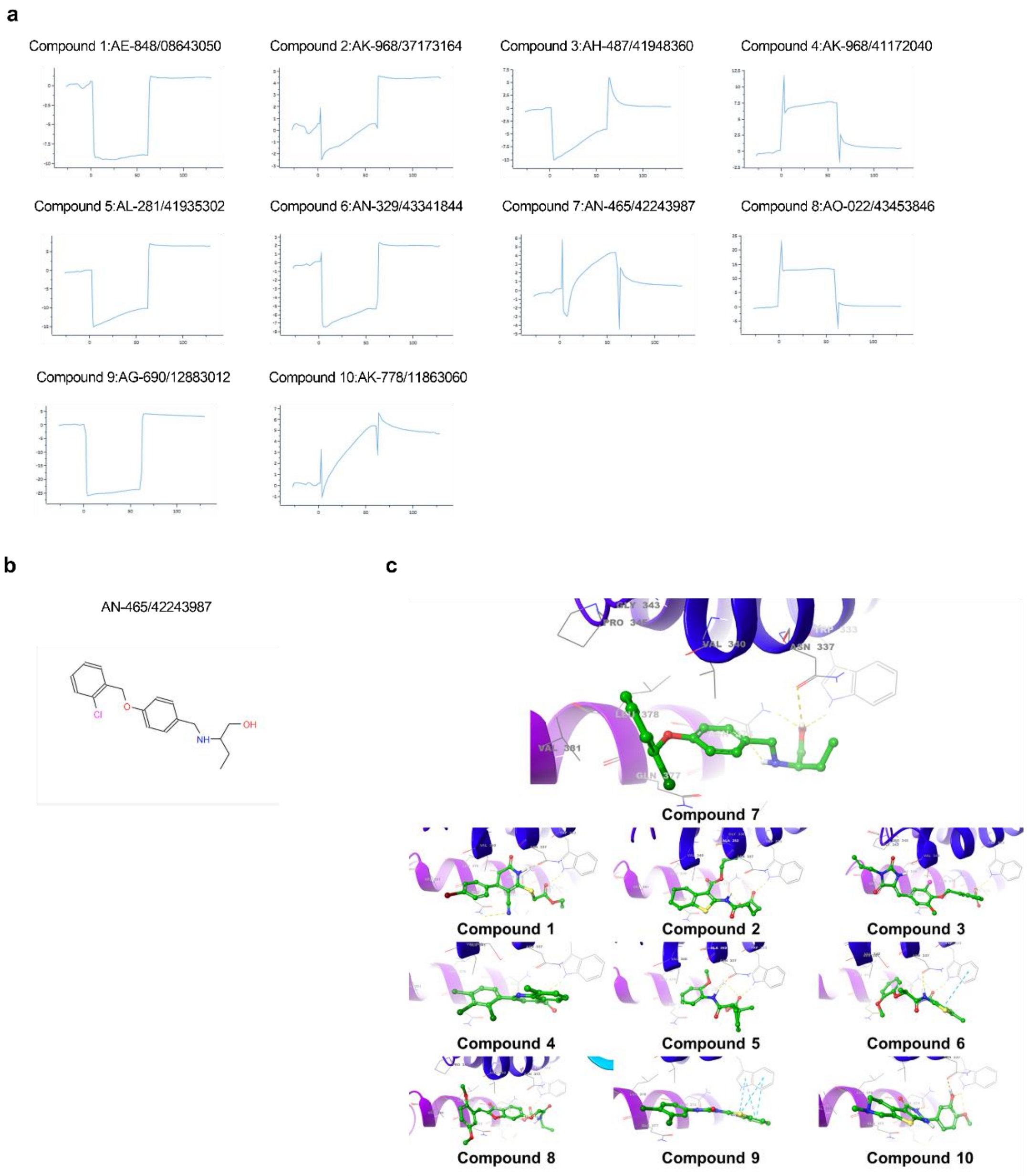

Supplementary Fig.10 Screen potential GPSM1 inhibitors.

a, SPR assay with Biacore to verify the affinity between GPSM1 protein and small-molecular compounds. The compounds were tested for binding with concentration of 50 μmol/L. **b**, The chemical structure of compound 7 (AN-465/42243987). **c**, Docking poses for the top 10 molecules. Ligands (green) and interacted residues (gray) in the receptor are represented as stick. The yellow dash line stands for hydrogen bond and π-π interaction is exhibited as the cyan dash line.

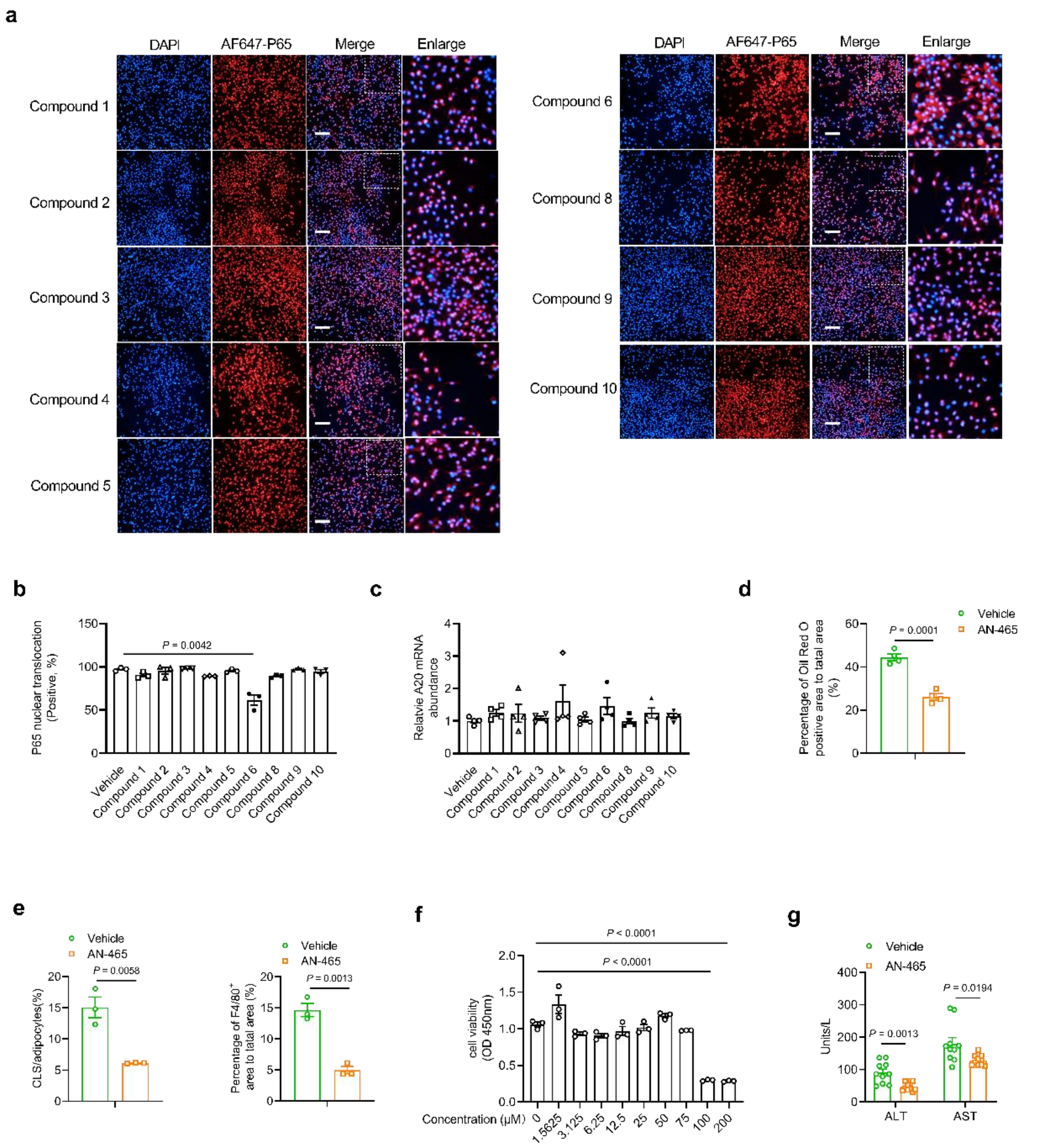

Supplementary Fig.11 The effects of potential GPSM1 inhibitors in *in vitro* and *in vivo* experiments.

a, Representative immunofluorescence images indicating P65 nuclei translocation of BMDMs treated with 50μM other 9 small-molecular compounds for 16 hours and then stimulated with LPS for 1 hour, showed by high-content screen. BMDMs were stained for P65 (red) and DAPI (blue). **b**, Quantification of the proportion of P65 nuclear translocation exhibited in **a** (n= 3 independent samples per group). **c**, RT-PCR indicating A20 mRNA levels of BMDMs treated with 50μM compounds or vehicle control for 16 hours and then stimulated with LPS for 20 min (n= 4 independent samples per group). **d**, Percentage of Oil Red O^{+} area of liver from DIO mice treated with either AN-465 or vehicle (n = 4

biologically independent mice per group). **e**, Quantification of the proportion of crown-like structure and F4/80$^+$ area of eWAT (n = 4 biologically independent mice per group). **f**, CCK8 assays were performed in BMDMs after cells were treated with increasing doses of AN-465/42243987 (n= 3 independent samples per group). **g**, Serum ALT and AST levels of DIO mice treated with either AN-465 or vehicle (n = 10 biologically independent mice per group). Independent experiments were repeated three times with similar results (**a**). All data are presented as means ± SEM. *P* values are determined by two-tailed Student's *t*-test (**b** to **g**).

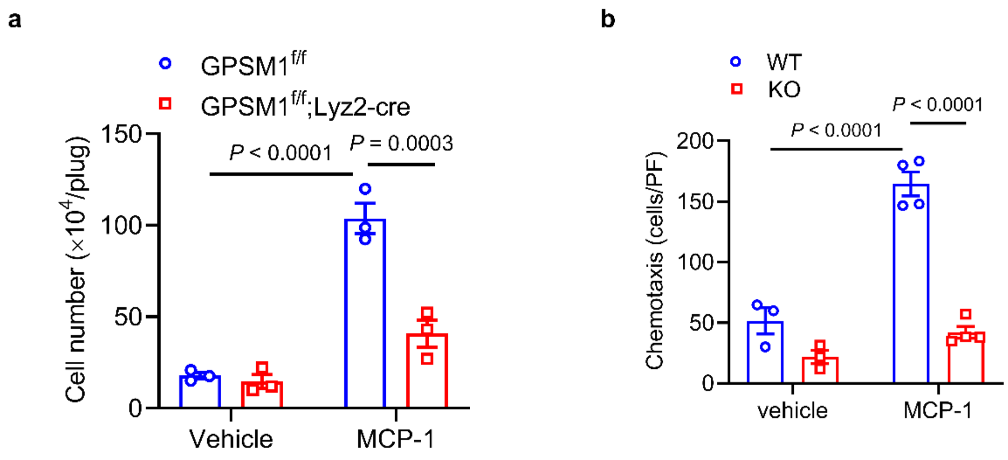

Supplementary Fig.12 Assessment of monocyte chemotactic activity *in vivo* and *in vitro*.

a, Recruitment monocyte-derived macrophages into implanted Matrigel plugs loaded with. MCP-1 of *GPSM1*$^{f/f}$ and *GPSM1*$^{f/f}$; *Lyz2*-cre mice (n = 3 biologically independent mice per group). **b**, Chemotaxis of WT and *GPSM1* KO THP-1 cells (n = 3 or 4 independent samples per condition). All data are presented as means ± SEM. *P* values are determined by one-way ANOVA with Tukey's multiple-comparisons test.

Supplementary Table 1. Clinical characteristics of subjects

Variable	Normal weight (n = 36)	Overweight/Obesity (n = 61)	*P* value
Male/ female (n)	6/30	13/48	0.5823
Age (years)	44.33±10.66	41.03±10.60	0.1427
BMI (kg/m^2)	21.85±1.56	33.01±7.06	4.5082×10^{-15}
SBP (mmHg)	120.86±12.83	129.08±17.05	0.0152
DBP (mmHg)	80.14±9.47	82.50±13.53	0.3662
Fasting plasma glucose (mmol/l)	4.83 (4.51, 5.61)	5.11 (4.75, 5.70)	0.2742
HbA1c (%)	5.50 (5.40, 6.40)	5.80 (5.10, 6.60)	0.7140
Triglyceride (mmol/l)	0.94 (0.71, 1.07)	1.57 (1.00, 2.31)	0.0018
Total cholesterol (mmol/l)	4.56 (3.98, 5.31)	4.83 (4.48, 5.30)	0.2255
ALT(U/L)	16.00 (13.00, 20.00)	26.00 (17.50, 52.00)	0.0049
AST(U/L)	22.00 (18.00, 26.00)	22.00 (18.00, 33.50)	0.1422
Low-Density Lipoprotein-c	2.59 (2.17, 3.27)	2.94 (2.43, 3.53)	0.1901
High-Density Lipoprotein-c	1.12 (0.88, 1.29)	1.06 (0.94, 1.19)	0.5238

Normal weight: BMI < 24; Overweight/Obesity: BMI ≥ 24

Data are presented as the mean ± SD or median (interquartile range) or n.

Comparisons are done using two-tailed Student's t-test.

Supplementary Table 2. Oligonucleotide primers for Loxp sites

	**Forward primer (5'-3')**	**Reverse primer (5'-3')**
GPSM1-Loxp primer1	CCCAGAAATGCCAGATTACG	CTTGGGCTGCCAGAATTTCTC
GPSM1-Loxp primer2	TTACAGTCGGCCAGGCTGAC	CTTGGGCTGCCAGAATTTCTC
A20-Loxp primer	CTATCTGTGGTGGACAAAGGCT ACTCTCGG	GAATCGCCTACCTAGGAATCAG CTGTCCAG

Supplementary Table 3. Oligonucleotide primers for ChIP and luciferase reporter assays

	**Forward primer (5'-3')**	**Reverse primer (5'-3')**
ChIP-Tnfaip3	ACCTATTGCATTTCCAGTTCCCA	GAGAAACTCCTAGGTCCCGC
ChIP-β-globin	AAGCCTGATTCCGTAGAGCCACAC	CCCACAGGCAAGAGACAGCAGC
PGL4.17-*Tnfaip3* (promoter)-WT	CTGAGCTCGCTAGCCTCGAGCCTT ACTGGCGAGAGGAGGA	ACCGGATTGCCAAGCTTTCGCAAA GTCCCAAGTCCTG
PGL4.17-*Tnfaip3* (promoter)-Mut	Primer1: CTGAGCTCGCTAGCCTCGAGCCTT ACTGGCGAGAGGAGGA<br>Primer2: ATTTCCACATGGATGTTTTTTTTCCC CAGCTTCCGAAA	Primer1: TTTCGGAAGCTGGGGAAAAAAAAC ATCCATGTGGAAAT<br>Primer2: ACCGGATTGCCAAGCTTTCGCAAA GTCCCAAGTCCTG

Supplementary Table 4. Oligonucleotide primers for quantitative RT-PCR analysis

Gene	Forward primer (5'-3')	Reverse primer (5'-3')
36b4	AAGCGCGTCCTGGCATTGTCT	CCGCAGGGGCAGCAGTGGT
Acc1	ATGGGCGGAATGGTCTCTTTC	TGGGGACCTTGTCTTCATCAT
Acta2	GTCCCAGACATCAGGGAGTAA	TCGGATACTTCAGCGTCAGGA
Actin	AGTGTGACGTTGACATCCGTA	GCCAGAGCAGTAATCTCCTTCT
Arg1	CTCCAAGCCAAAGTCCTTAGAG	GGAGCTGTCATTAGGGACATCA
Catalase	ACATGGTCTGGGACTTCTGG	CAAGTTTTTGATGCCCTGGT
Ccl2	TTAAAAACCTGGATCGGAACCAA	GCATTAGCTTCAGATTTACGGGT
Ccl3	CATGACACTCTGCAACCAAGTCTTC	GAGCAAAGGCTGCTGGTTTCA
Ccl7	CAATGCATCCACATGCTGCTA	GACCCACTTCTGATGGGCTTC
CD36	ATGGGCTGTGATCGGAACTG	GTCTTCCCAATAAGCATGTCTCC
CD45	TCCAGGTGTGTTATCCACGC	TGTGTCTGCAGGAATGGTCC
Col1a1	TGACTGGAAGAGCGGAGAGT	GTTCGGGCTGATGTACCAGT
Col3a1	GTGCTCCTGGACAGAATGGT	CACCCTTTACACCCTGAGGA
Col4a1	GCCAAGTGTGCATGAGAAGA	AGCGGGGTGTGTTAGTTACG
Col6a1	GATGAGGGTGAAGTGGGAGA	CAGCACGAAGAGGATGTCAA
Col6a2	ATGTGAGGGAGACCTGTGGA	TGTGCCTGTTTCTGACTTGG
Col6a3	CAGAACCATTGTTTCTCACT	AGGACTACACATCTTTTCAC
Cpt-1a	CTCAGTGGGAGCGACTCTTCA	GGCCTCTGTGGTACACGACAA
Cxcl1	TGCACCCAAACCGAAGTC	GTCAGAAGCCAGCGTTCACC
Fabp4	CAGCGTAAATGGGGATTTGG	CCGCCATCTAGGGTTATGAT
Fasn	GGAGGTGGTGATAGCCGGTAT	TGGGTAATCCATAGAGCCCAG
Gpsm1	CTTTCTTCGAGGCTGCTGTG	TCATCGAATCGGCCTAGGAC
IL-10	GCTATGCTGCCTGCTCTTACT	CCTGCTGATCCTCATGCCA
IL-1β	GCAACTGTTCCTGAACTCAACT	ATCTTTTGGGGTCCGTCAACT
IL-6	CCACGGCCTTCCCTACTTC	TTGGGAGTGGTATCCTCTGTGA
Lcad	GCGAAATACTGGGCATCTGAA	TCCGTGGAGTTGCACACATT
Leptin	GACACCAAAACCCTCAT	CAGTGTCTGGTCCATCT
Mcad	GACATTTGGAAAGCTGCTAGTG	TCACGAGCTATGATCAGCCTCTG
Mmp2	TAACCTGGATGCCGTCGT	TTCAGGTAATAAGCACCCTTGAA
Mmp9	CGTCGTGATCCCCACTTACT	AACACACAGGGTTTGCCTTC
Mrc1	CTCTGTTCAGCTATTGGACGC	TGGCACTCCCAAACATAATTTGA
Nos2	GTTCTCAGCCCAACAATACAAGA	GTGGACGGGTCGATGTCAC
Pdk4	TTCACACCTTCACCACATGC	AAAGGGCGGTTTTCTTGATG
Retnla	CCAATCCAGCTAACTATCCCTCC	ACCCAGTAGCAGTCATCCCA
S100A8	GGAAATCACCATGCCCTCTA	TGGCTGTCTTTGTGAGATGC
Scd1	GCTGGAGTACGTCTGGAGGAA	TCCCGAAGAGGCAGGTGTAG
Srebp-1c	GCAGCCACCATCTAGCCTG	CAGCAGTGAGTCTGCCTTGAT
Tgfb1	GTGTGGAGCAACATGTGGAACTCTA	TTGGTTCAGCCACTGCCGTA
TNF-a	GACGTGGAACTGGCAGAAGAG	ACCGCCTGGAGTTCTGGAA
Ucp2	GCTGGTGGTGGTCGGAGATA	ACTGGCCCAAGGCAGAGTT

Gel source data

Supplemental figure 3a

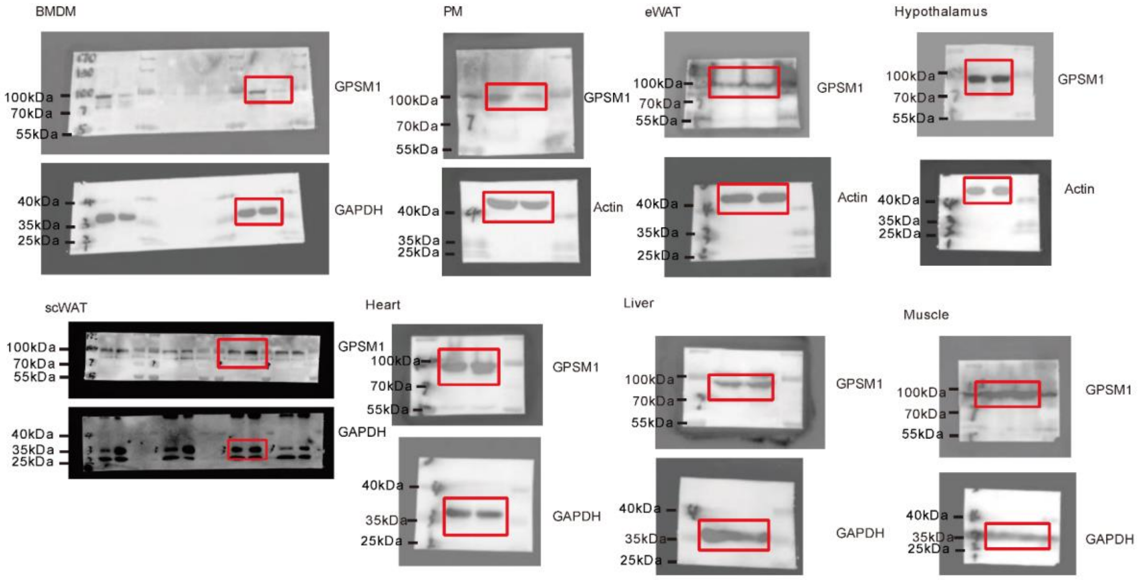

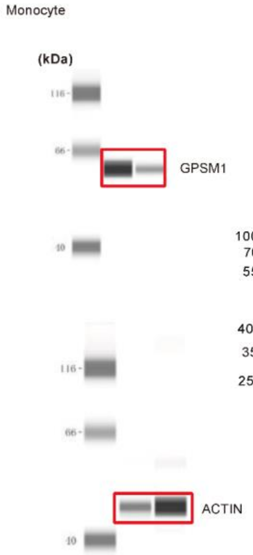

Supplemental figure 9a

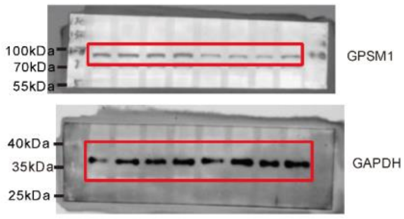

Supplemental figure 9b

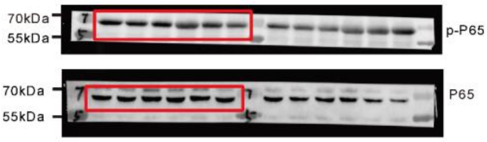

Supplement: Supplementary file 1 — Supplementary information [file 41467_2022_34998_MOESM1_ESM.pdf]
