# Supplementary material for: GPSM1 impairs metabolic homeostasis by controlling a pro-inflammatory pathway in macrophages
Source: Nat Commun. 2022 Nov 25;13:7260. doi: 10.1038/s41467-022-34998-9 (PMC9700814; doi:10.1038/s41467-022-34998-9)
Supplement: Supplementary file 2 — Reporting Summary [file 41467_2022_34998_MOESM2_ESM.pdf]

nature portfolio

Corresponding author(s): Cheng Hu

Last updated by author(s): Nov 10, 2022

Reporting Summary

Nature Portfolio wishes to improve the reproducibility of the work that we publish. This form provides structure for consistency and transparency in reporting. For further information on Nature Portfolio policies, see our Editorial Policies and the Editorial Policy Checklist.

Statistics

For all statistical analyses, confirm that the following items are present in the figure legend, table legend, main text, or Methods section.

n/a	Confirmed	
☐	☒	The exact sample size (*n*) for each experimental group/condition, given as a discrete number and unit of measurement
☐	☒	A statement on whether measurements were taken from distinct samples or whether the same sample was measured repeatedly
☐	☒	The statistical test(s) used AND whether they are one- or two-sided<br>*Only common tests should be described solely by name; describe more complex techniques in the Methods section.*
☒	☐	A description of all covariates tested
☐	☒	A description of any assumptions or corrections, such as tests of normality and adjustment for multiple comparisons
☐	☒	A full description of the statistical parameters including central tendency (e.g. means) or other basic estimates (e.g. regression coefficient) AND variation (e.g. standard deviation) or associated estimates of uncertainty (e.g. confidence intervals)
☐	☒	For null hypothesis testing, the test statistic (e.g. *F*, *t*, *r*) with confidence intervals, effect sizes, degrees of freedom and *P* value noted<br>*Give P values as exact values whenever suitable.*
☒	☐	For Bayesian analysis, information on the choice of priors and Markov chain Monte Carlo settings
☒	☐	For hierarchical and complex designs, identification of the appropriate level for tests and full reporting of outcomes
☐	☒	Estimates of effect sizes (e.g. Cohen's *d*, Pearson's *r*), indicating how they were calculated

Our web collection on statistics for biologists contains articles on many of the points above.

Software and code

Policy information about availability of computer code

Data collection	QuantStudio Real-Time PCR System (Applied Biosystmes) for qPCR reactions;<br>ChemiDoc Imaging System (Bio-Rad) for immunoblot data collection;<br>ImageSteam Mark II (Luminex) for flow cytometry data collection;<br>BIAcore 8K (GE Healthcare) for the Surface Plasmon Resonance;<br>Operetta® CLS™ high-content analysis system for small-molecule compound screening;<br>OxyMax Comprehensive Laboratory Animal Monitoring System (CLAMs, Columbus Instruments) for energy expenditure data collection;<br>Leica Microsystems CMS GmbH (Leica) for IHC and IF data collection;<br>Illumina Novaseq 6000 (Illumina) for the RNA-seq libraries;<br>Jess System (ProteinSimple) for Capillary eletrophoresis;

Data analysis	GraphPad Prism version 8.3.0 was used to generate graphs and perform statistical analysis;<br>IDEAS software version 6.2 for FACS analysis;<br>Image Lab software version 6.0 for acquisition of images from Western blot;<br>ImageJ (National Institutes of Health) version 1.52 for Western blot and IHC images densitometry analysis;<br>SAS software version 8.0 for statistical analysis;<br>RSeQC software version 2.6.4 for quality of RNA-seq data;<br>Biacore Insight Evaluation version 3.0.12 for Surface Plasmon Resonance;<br>Harmony software (Perkin Elmer) version 4.9 for High-content images analysis.<br>Compass for SW 6.1.0 version for Capillary eletrophoresis;<br>AlloSite version 2.0 web server (http://mdl.shsmu.edu.cn/AST/) for predicting potential allosteric pocket.

For manuscripts utilizing custom algorithms or software that are central to the research but not yet described in published literature, software must be made available to editors and reviewers. We strongly encourage code deposition in a community repository (e.g. GitHub). See the Nature Portfolio guidelines for submitting code & software for further information.

Data

Policy information about availability of data

All manuscripts must include a data availability statement. This statement should provide the following information, where applicable:

- Accession codes, unique identifiers, or web links for publicly available datasets
- A description of any restrictions on data availability
- For clinical datasets or third party data, please ensure that the statement adheres to our policy

All data supporting the findings of this study are available within the main manuscript and the supplementary files. RNA-seq data can be viewed in NODE under the accession code OEP003692 or through the URL: https://www.biosino.org/node/project/detail/OEP003692. Source data are provide with this paper. A reporting summary for this article is available as a supplementary file.

Human research participants

Policy information about studies involving human research participants and Sex and Gender in Research.

Reporting on sex and gender	Sex was comparable between the two groups we analyzed.
Population characteristics	97 subjects were recruited in the study. Donors were without/with overweight or obesity (BMI < 24, n = 36 biologically independent individuals; BMI ≥ 24, n = 61 biologically independent individuals) according to the Working Group on Obesity in China criteria. Sex and Age were comparable between the two groups. All Clinical characteristics are shown in the Supplementary Table 1.
Recruitment	Donors were recruited from Department of Bariatric and Metabolic Surgery, Shanghai Sixth People's Hospital affiliated to Shanghai Jiao Tong University School of Medicine. Visceral fat were obtained from age- and gender-matched human donors undergoing non-acute surgical interventions, in a scheduled routine surgery.
Ethics oversight	The human study was approved by the Human Research Ethics Committee of Shanghai Sixth People's Hospital affiliated to Shanghai Jiao Tong University School of Medicine. All individuals gave informed consent.

Note that full information on the approval of the study protocol must also be provided in the manuscript.

Field-specific reporting

Please select the one below that is the best fit for your research. If you are not sure, read the appropriate sections before making your selection.

☒ Life sciences ☐ Behavioural & social sciences ☐ Ecological, evolutionary & environmental sciences

For a reference copy of the document with all sections, see nature.com/documents/nr-reporting-summary-flat.pdf

Life sciences study design

All studies must disclose on these points even when the disclosure is negative.

Sample size	Sample size for animal studies was determined based on published literature and previous lab experience. For cellular studies, the sample size was chosen according to preliminary experiments. At least three biological replicates per group (detailed n is indicated in the figure or figure legends) were collected to perform statistical testing.
Data exclusions	Data points were excluded only for undetectable CT values of qPCR reactions.
Replication	For mouse experiments, data are representative of at least three independent experiments. For cellular experiments, data collected from at least three independent experiments. All additional replication attempts were successful.

Randomization	Animal were randomly assigned to different experimental groups and processed.
Blinding	Blinding was not done as all the analyses were performed quantitatively and not subjectively.

Reporting for specific materials, systems and methods

We require information from authors about some types of materials, experimental systems and methods used in many studies. Here, indicate whether each material, system or method listed is relevant to your study. If you are not sure if a list item applies to your research, read the appropriate section before selecting a response.

Materials & experimental systems

n/a	Involved in the study
☐	☒ Antibodies
☐	☒ Eukaryotic cell lines
☒	☐ Palaeontology and archaeology
☐	☒ Animals and other organisms
☒	☐ Clinical data
☒	☐ Dual use research of concern

Methods

n/a	Involved in the study
☒	☐ ChIP-seq
☐	☒ Flow cytometry
☒	☐ MRI-based neuroimaging

Antibodies

Antibodies used

For Western blot, the following antibodies were used:
β-Actin (1:2500, 4970), HSP90 (1:2500, 4877), Lamin A/C (1:1000, 4777), Phospho-NF-κB p65 (1:1000, 3033), NF-κB p65 (1:2000, 8242), Phospho-IκBα (1:1000, 9246), IκBα (1:1000, 9242), Phospho-IKKα/β (1:1000, 2697), IKKβ (1:1000, 2678), Phospho-AKT (1:1000, 4058), AKT (1:1000, 9272), IL-1β (1:1000, 12242), Cleaved-IL-1β (1:1000, 63124), Caspase-1 (1:1000, 24232), A20/TNFAIP3 (1:1000, 5630) , Phospho-(Ser/Thr) PKA Substrate (1:1000, 9621), Phospho-CREB (1:1000, 9198), and CREB(1:1000, 9197) were from Cell Signaling. GAPDH (1:2500, sc-32233) was obtained from Santa Cruz Biotechnology. UCP1 (1:1000, ab10983) was from Abcam. GPSM1 (1:1000, 11483-1-AP) was from Proteintech. Cleaved-Caspase-1 (1:500, AG-20B-0042) was from AdipoGen.

For immunofluorescent staining and immunohistochemical staining, the following antibodies were used:
F4/80 (eBioscience, 14-4801-82, 1:50), GPSM1 (Proteintech, 11483-1-AP, 1:50), DAPI (Beyotime Biotechnology, C1002, 1:5000), p-p65 (CST, 3033, 1:50), Alexa Fluor 488 donkey anti-mouse IgG (Beyotime, R37114, 1:1000), Alexa Fluor 647 donkey anti-rabbit IgG (Beyotime, A-31573, 1:1000), Alexa Fluor 546 donkey anti-rabbit IgG (Beyotime, A10040, 1:1000).

For FACS analysis:
For SVFs isolated from mice scWAT and eWAT, PE-CY7-anti-CD45 (552848, BD Pharmingen, 0.06 μg/test), FITC-anti-F4/80 (11-4801-82, eBioscience, 0.5 μg/test), APC-anti-F4/80 (17-4801-82, eBioscience, 1 μg/test), APC-anti-CD11b (553312, BD Pharmingen, 0.6 μg/test), APC-anti-CD11c (550261, BD Pharmingen, 0.6 μg/test), AF647-CD206 (565250, BD Pharmingen, 0.6 μg/test), and AF488-TIM-4 (53-5866-82, eBioscience, 1 μg/test) were used. The antibodies used for neutrophil sorting included FITC-anti-Gr-1 (11-5931-82, eBioscience, 0.25 μg/test) and APC-anti-CD11b (553312, BD Pharmingen, 0.6 μg/test). For bone marrow cells collected from leg bones, anti-FcγRII/III antibody 2.4G2 (553141, BD Pharmingen, 1:140), FITC-anti-mouse hematopoietic lineage (22-7770-72, eBioscience, 20 μl/test), APC-anti-SCA1 (17-5981-82, eBioscience, 0.2 μg/test), and eFluor450-anti-CD117 (48-1171-82, eBioscience, 0.25 μg/test) were used. For peripheral blood monocyte analysis, anti-FcγRII/III antibody 2.4G2, APC-anti-CD115 (17-1152-82, eBioscience, 0.2 μg/test), FITC-anti-CD11b (557396, BD Pharmingen, 1.5 μg/test), and PE-CY7-anti-LY6C (25-5932-82, eBioscience, 0.125 μg/test) were used. Dead cells were excluded from all samples using DAPI, fixable viability stain 780 (565388, BD Pharmingen, 1:1000), and fixable viability stain 510 (564406, BD Pharmingen, 1:1000).

For ChIP assay, the following antibodies were used:
rabbit anti- Phospho-CREB (Ser133) antibody (Cell Signaling Technology, 9198, 1:50), normal rabbit IgG (Cell Signaling Technology, 2729, 1:250).

For High-content screening, the following antibodies were used:
p65 (Cell Signaling Technology, 8242, 1:75), Alexa Fluor 647-labeled Goat Anti-Rabbit IgG (Beyotime, A0468, 1:500), DAPI (Beyotime, C1002, 1:2000).

Validation

Antibodies used in this study were commercially available and were selected from published literature. Primary antibodies were validated for: specificity using cell lines expressing or not the target. Fixation, permeabilization and incubation were optimized to obtain the higher signal-to-noise ratio for each antibody.

Eukaryotic cell lines

Policy information about cell lines and Sex and Gender in Research

Cell line source(s)	HEK293T cell lines and THP-1 cell lines were obtained form Bank of Type Culture Collection of the Chinese Academy of Sciences.

Authentication	All cell lines were authenticated by morphology, gene expression profile and differentiation potentiality.
Mycoplasma contamination	Cell lines were tested negative for mycoplasma.
Commonly misidentified lines (See ICLAC register)	No commonly misidentified cell lines were involved in this study.

Animals and other research organisms

Policy information about studies involving animals; ARRIVE guidelines recommended for reporting animal research, and Sex and Gender in Research

Laboratory animals	C57BL/6J mice were purchased from GemPharmatech Co. Ltd. Lyz2-cre mice, which express the Cre recombinase transgene under the control of the lysozyme 2 gene promoter/enhancer elements, were purchased from the Jackson Laboratory (Stock No. 004781). GPSM1f/f mice were generated under the C57BL/6J genetic background by GemPharmatech Co. Ltd, in which exon 2–11 of the GPSM1 allele was flanked by loxP sites. Myeloid cell-specific GPSM1 knockout mice (GPSM1f/f; Lyz2-Cre) were subsequently produced via intercrossing GPSM1f/f mice with heterozygous Lyz2-Cre mice. Myeloid cell-specific TNFAIP3 heterozygous KO (TNFAIP3f/+; Lyz2-cre) mice were generated by mating TNFAIP3flox/+ mice with heterozygous Lyz2-cre mice. Myeloid-cell specific GPSM1 KO TNFAIP3 heterozygous KO mice (GPSM1f/fTNFAIP3f/+; Lyz2-cre) mice were generated by mating GPSM1f/f; TNFAIP3f/+mice with heterozygous Lyz2-cre mice. TNFAIP3flox/+ mice were kindly provide by Dr. Yang Xiao and Dr. Shanshan Liu (The Second Xiangya Hospital of Central South University).<br>Animals were housed in laboratory cages at 22–24°C under controlled conditions (12 h light/dark cycle) with free access to food and water. The animals were housed with 3–5 mice per cage. Except for obesity, the mice were in generally good health. Mice were maintained on an NCD (P1200F, Shanghai Puluteng Co. Ltd) or a HFD (60% kcal fat; D12492, Research Diets). For diet-induced obesity, eight-week-old mice were fed for 8 or 12 weeks (for male) and 14 weeks (for female).
Wild animals	The study did not involve wild animals.
Reporting on sex	Males and Females were used for analyze metabolic phenotypes.
Field-collected samples	The study did not involve samples collected from the field.
Ethics oversight	Experiments involving mice were all in accordance with institutional guidelines for the care and use of animals. Animal protocols were approved by the Animal Care Committee of Shanghai Sixth People's Hospital affiliated to Shanghai Jiao Tong University School of Medicine, and the animal welfare ethics acceptance number is YS-2018-059. Except for obesity, the mice were in generally good health without stress or pain. The study did not have humane endpoints. Before euthanasia by cervical dislocation, animals were anesthetized with isoflurane (3% in oxygen).

Note that full information on the approval of the study protocol must also be provided in the manuscript.

Flow Cytometry

Plots

Confirm that:

☒ The axis labels state the marker and fluorochrome used (e.g. CD4-FITC).

☒ The axis scales are clearly visible. Include numbers along axes only for bottom left plot of group (a 'group' is an analysis of identical markers).

☒ All plots are contour plots with outliers or pseudocolor plots.

☒ A numerical value for number of cells or percentage (with statistics) is provided.

Methodology

Sample preparation	For SVFs isolated from mice scWAT and eWAT, SVFs were isolated from mice scWAT and eWAT using 0.2% collagenase type 2 digestion. Briefly, adipose depots were collected and minced into pieces, followed by digestion at 37°C for 30 minutes. Cell suspensions were filtered through 100μM filters and centrifuged at 1,200 ×g for 10 min. Red blood cells were lysed and SVF pellets were washed with stain buffer, and then stained with indicated fluorescent-conjugated antibodies for 30 min at 4 °C in the dark.<br>For bone marrow cells collected from leg bones, cells were lysed to remove RBCs and filtered before use. Fc receptors were blocked using anti-FcγRII/III antibody 2.4G2 prior to adding the fluorescent-tagged antibodies.<br>For peripheral blood monocyte analysis, 100 μl of blood was collected into EDTA tubes before RBC lysis, filtration, and staining, for 30 min on ice. Fc receptors were blocked using anti-FcγRII/III antibody 2.4G2.
Instrument	ImageSteam Mark II (Luminex)
Software	IDEAS software(version 6.2) (Luminex)

Cell population abundance	We stained isolated SVFs by FACS with F4/80, CD11b, CD11c, CD206 and Tim4 antibodies. Our flow cytometry analysis revealed that we could obtain ~40% F4/80+CD11b+ macrophages in eWAT and scWAT SVFs.<br>We obtain 5~10% MPC (lineage-Sca1-cKit+) in the mouse bone marrow under both NCD and HFD setting.
Gating strategy	For analysis of SVFs, we set appropriate Gradient RMS, Area and Aspect Ratio intensity gates to exclude debris and cell aggregates. Then, we used DAPI to exclude dead cells, then draw a gate of CD45+ as immune populations, further draw the F4/80+CD11b+ population as macrophages. For analysis of M1 or M2-like macrophages, we draw CD45+ F4/80+first, and then we draw a gate around the CD11c+ population identified as M1-like macrophages or the CD206+ population identified as M2-like macrophages.<br>For analysis of Myeloid progenitor cells in the bone marrow, we set appropriate Gradient RMS, Area and Aspect Ratio intensity gates to exclude debris and cell aggregates. Then, we used FVS780 to exclude dead cells. Then we draw a gate of Lin-, and defined Sca1-cKit+ as MPC.<br>For analysis of blood monocytes and its subsets, we set appropriate Gradient RMS, Area and Aspect Ratio intensity gates to exclude debris and cell aggregates. Then, we used DAPI to exclude dead cells. We draw CD115+CD11b+ as monocytes, and further divided into Ly6chigh or Ly6clow sbusets.

☒ Tick this box to confirm that a figure exemplifying the gating strategy is provided in the Supplementary Information.
