# Supplementary material for: GPSM1 impairs metabolic homeostasis by controlling a pro-inflammatory pathway in macrophages
Source: Nat Commun. 2022 Nov 25;13:7260. doi: 10.1038/s41467-022-34998-9 (PMC9700814; doi:10.1038/s41467-022-34998-9)

Gel source data-1

figure 1b

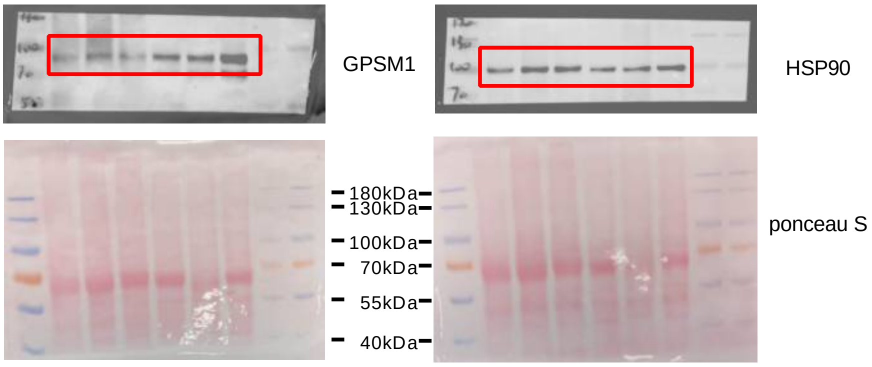

figure 1c

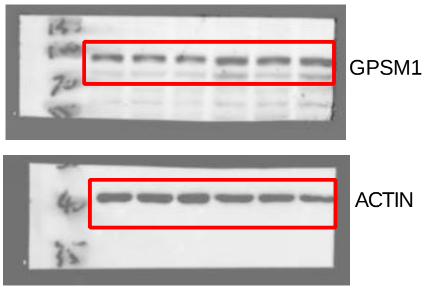

figure 1f

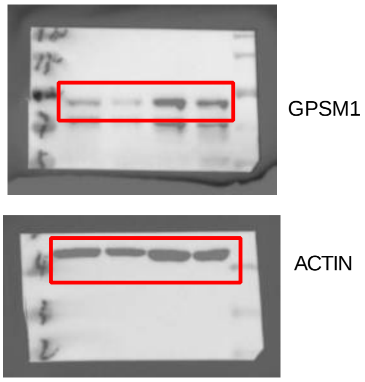

figure 1h

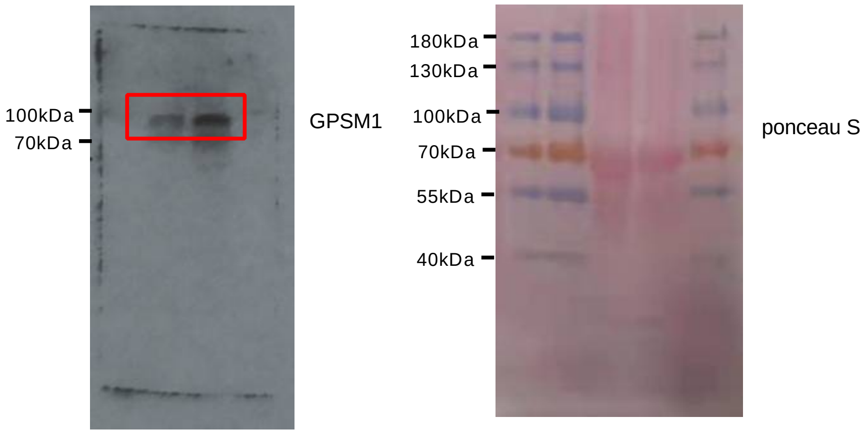

figure 2j

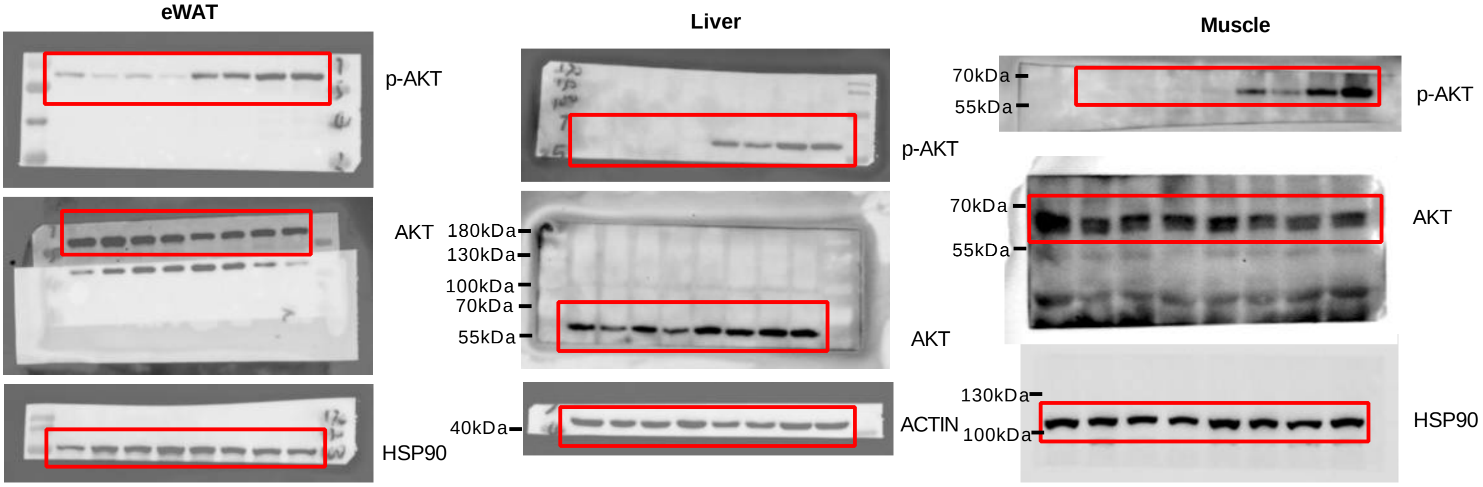

Gel source data-2

figure 4d

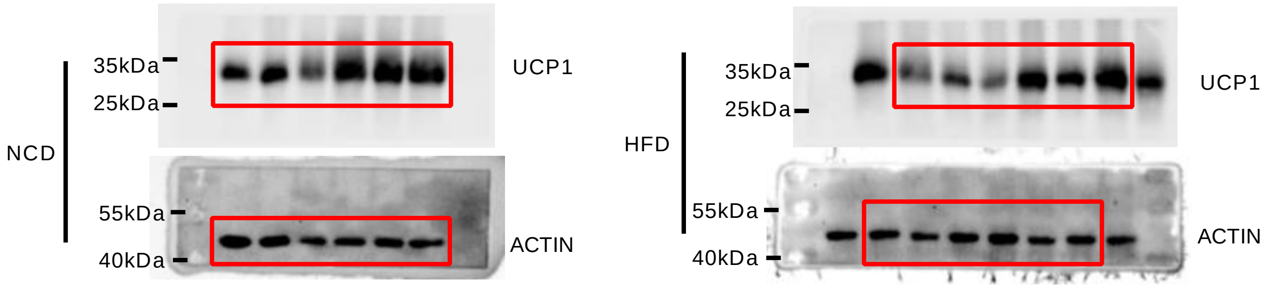

figure 5a

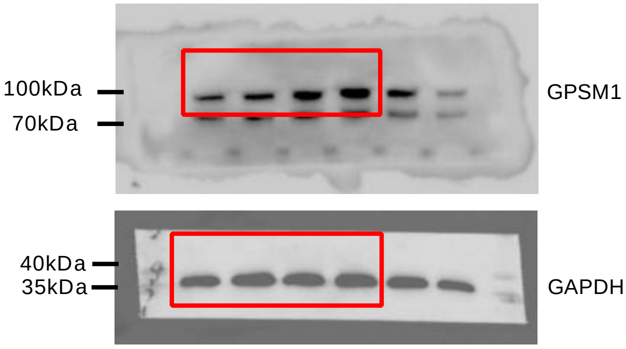

figure 5c

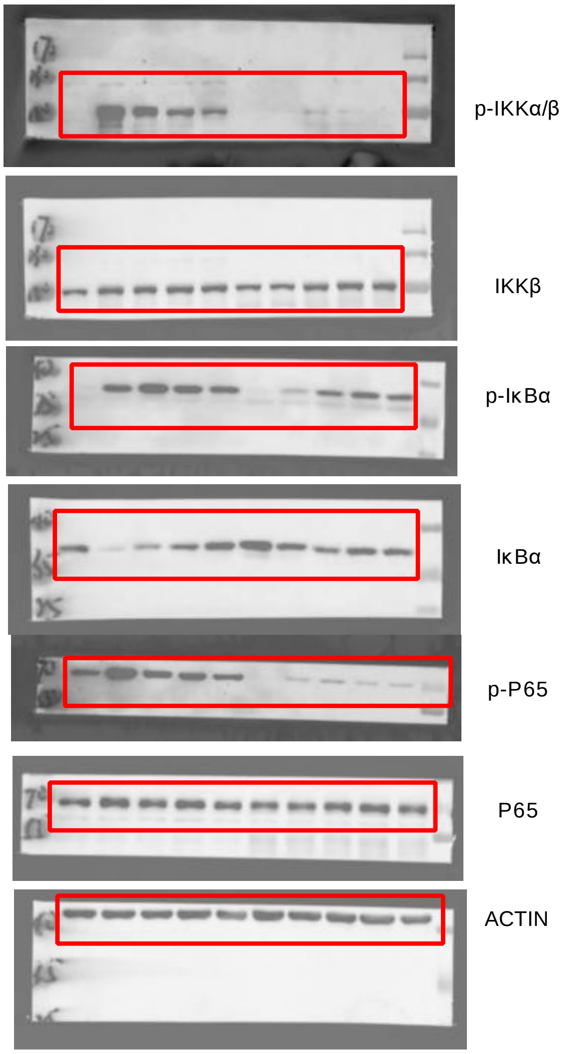

figure 5d

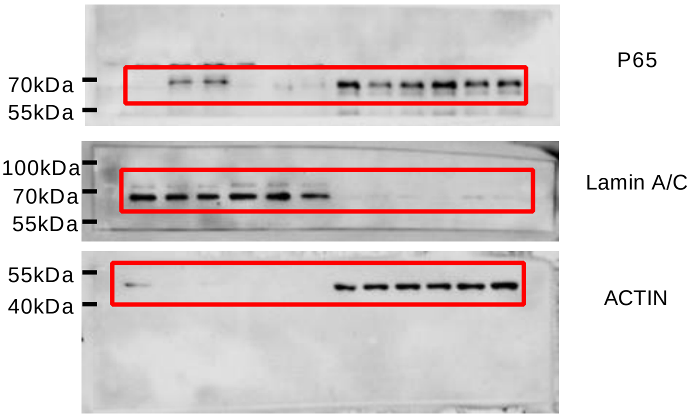

figure 5e

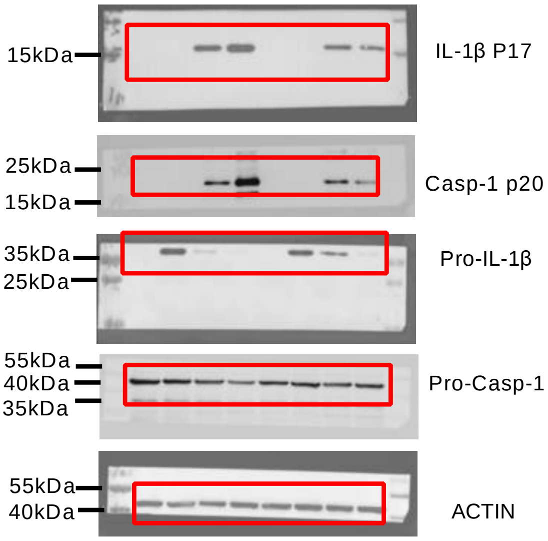

Gel source data-3

figure 5g

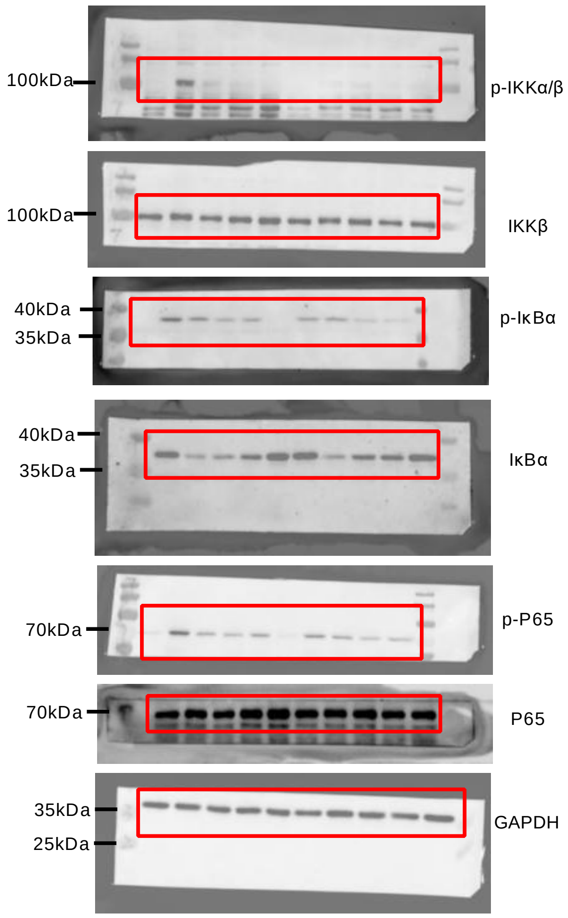

figure 5h

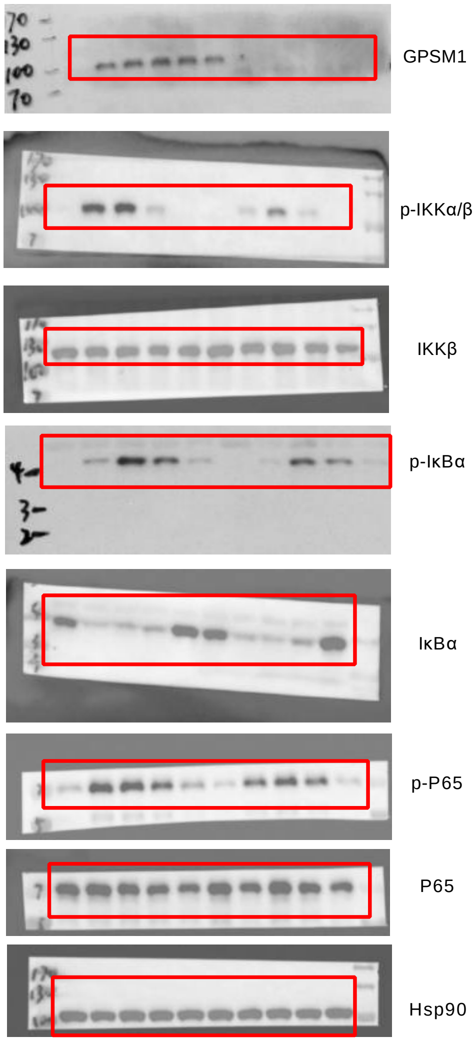

figure 5i

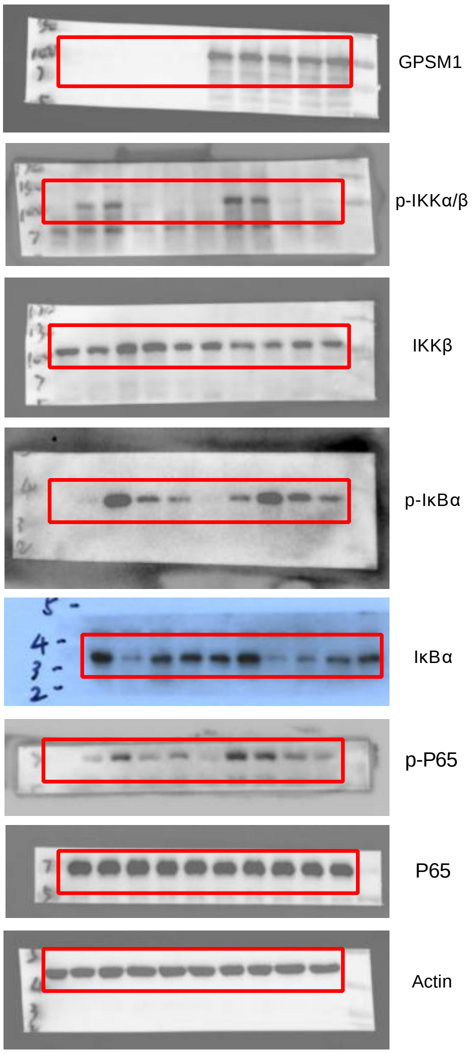

figure 6b

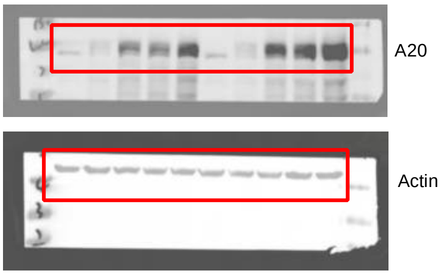

figure 6c

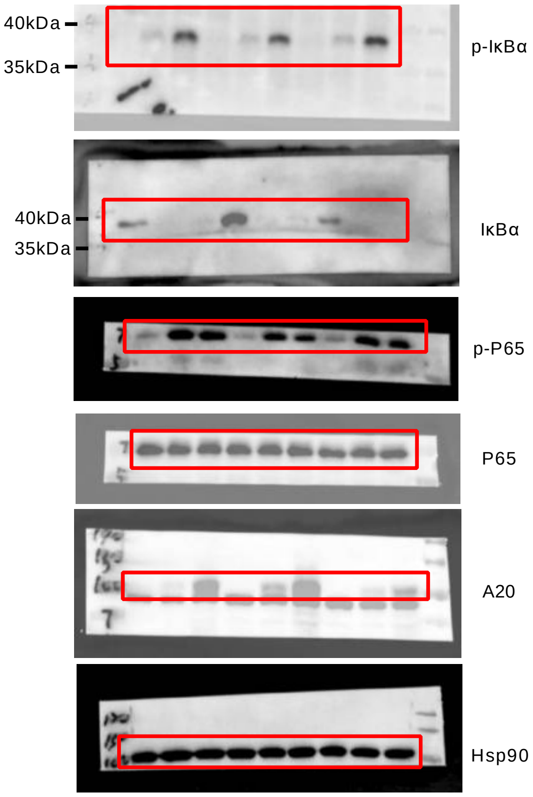

figure 6d

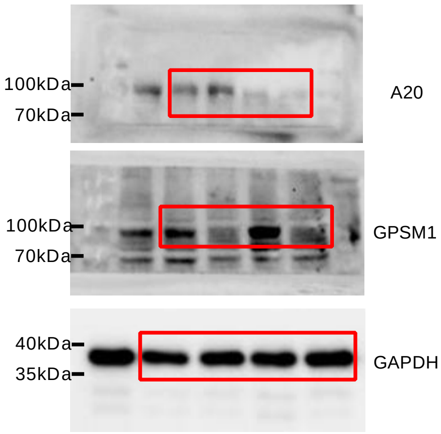

Gel source data-4

figure 7b

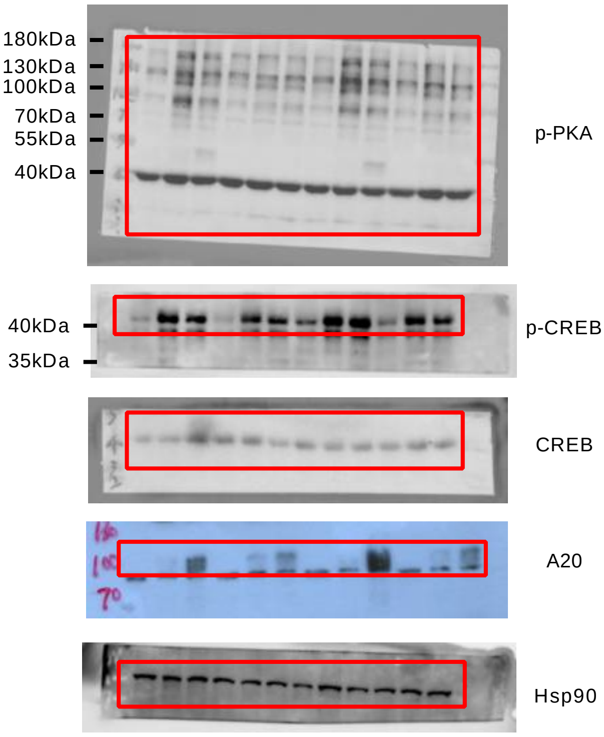

figure 7e

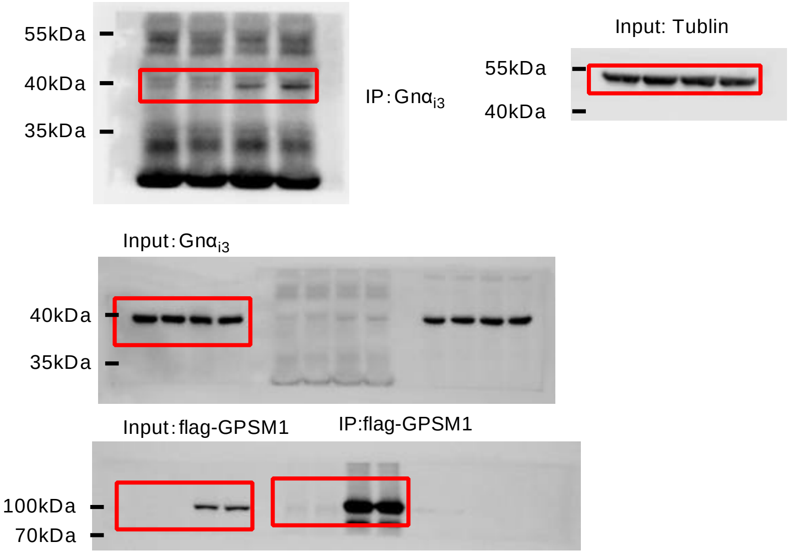

figure 7f

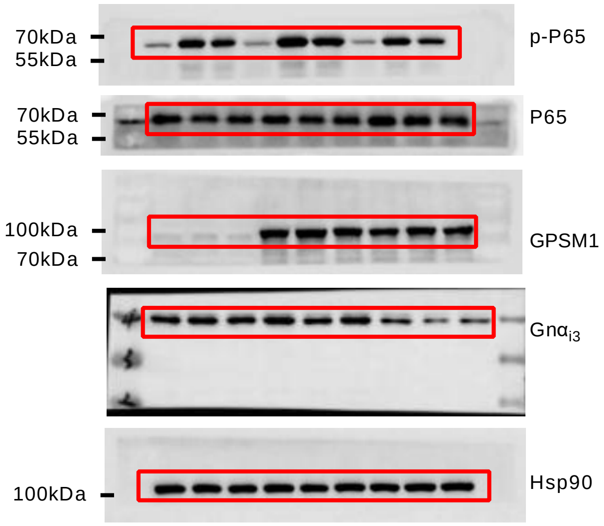

figure 8a

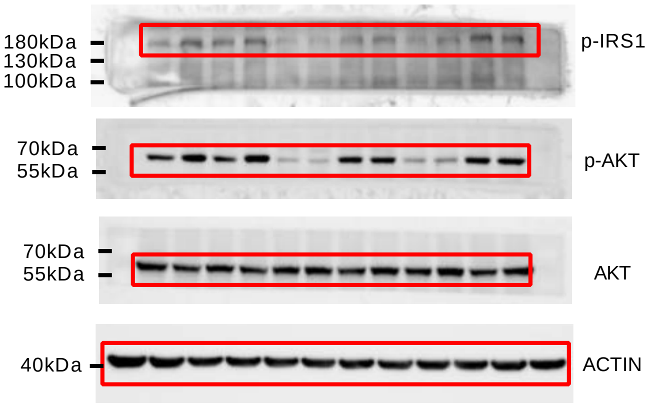

figure 8b

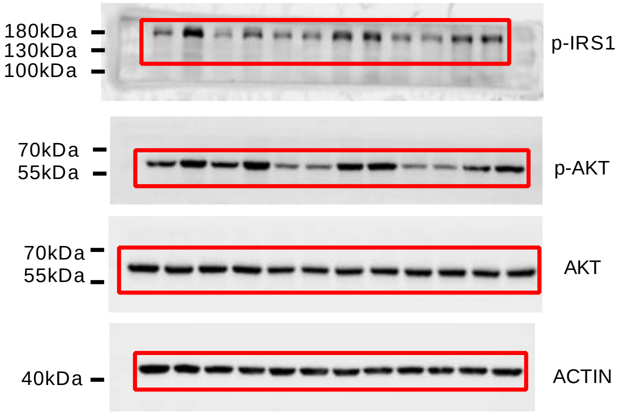

Supplement: Supplementary file 3 — Source Data [file 41467_2022_34998_MOESM3_ESM.zip › Source Data/Source Data/Gel source data.pdf]
